# Development and validation of the DIabetes Severity SCOre (DISSCO) in 139 626 individuals with type 2 diabetes: a retrospective cohort study

**DOI:** 10.1136/bmjdrc-2019-000962

**Published:** 2020-05-07

**Authors:** Salwa S Zghebi, Mamas A Mamas, Darren M Ashcroft, Chris Salisbury, Christian D Mallen, Carolyn A Chew-Graham, David Reeves, Harm Van Marwijk, Nadeem Qureshi, Stephen Weng, Tim Holt, Iain Buchan, Niels Peek, Sally Giles, Martin K Rutter, Evangelos Kontopantelis

**Affiliations:** 1 NIHR School for Primary Care Research, Centre for Primary Care and Health Services Research, Manchester Academic Health Science Centre (MAHSC), The University of Manchester, Manchester, UK; 2 Division of Population Health, Health Services Research and Primary Care, School of Health Sciences, Faculty of Biology, Medicine and Health, Manchester Academic Health Science Centre (MAHSC), The University of Manchester, Manchester, UK; 3 Keele Cardiovascular Research Group, Centre for Prognosis Research, School of Primary, Community and Social Care, Keele University, Stoke-on-Trent, UK; 4 Division of Pharmacy and Optometry, School of Health Sciences, Faculty of Biology, Medicine and Health, Manchester Academic Health Science Centre (MAHSC), The University of Manchester, Manchester, UK; 5 NIHR Greater Manchester Patient Safety Translational Research Centre, The University of Manchester, Manchester, UK; 6 NIHR Manchester Biomedical Research Centre, Manchester Academic Health Science Centre (MAHSC), Manchester, UK; 7 Centre for Academic Primary Care, Population Health Sciences, Bristol Medical School, University of Bristol, Bristol, UK; 8 School of Primary, Community and Social Care, Faculty of Medicine and Health Sciences, Keele University, Staffordshire, UK; 9 Centre for Biostatistics, School of Health Sciences, Faculty of Biology, Medicine and Health, Manchester Academic Health Science Centre (MAHSC), The University of Manchester, Manchester, UK; 10 Department of Primary Care and Public Health, Brighton and Sussex Medical School, University of Sussex, Falmer, UK; 11 Primary Care Stratified Medicine (PRISM) Research Group, Division of Primary Care, School of Medicine, University of Nottingham, Nottingham, UK; 12 Nuffield Department of Primary Care Health Sciences, University of Oxford, Oxford, UK; 13 Institute of Population Health, University of Liverpool, Liverpool, UK; 14 Division of Informatics, Imaging and Data Sciences, School of Health Sciences, Faculty of Biology, Medicine and Health, Manchester Academic Health Science Centre (MAHSC), The University of Manchester, Manchester, UK; 15 Manchester Diabetes Centre, Manchester University NHS Foundation Trust, Manchester Academic Health Science Centre (MAHSC), Manchester, UK; 16 Division of Diabetes, Endocrinology and Gastroenterology, School of Medical Sciences, Faculty of Biology, Medicine and Health, Manchester Academic Health Science Centre (MAHSC), The University of Manchester, Manchester, UK

**Keywords:** type 2 diabetes, electronic patient records, algorithms, hospitalization

## Abstract

**Objective:**

Clinically applicable diabetes severity measures are lacking, with no previous studies comparing their predictive value with glycated hemoglobin (HbA_1c_). We developed and validated a type 2 diabetes severity score (the DIabetes Severity SCOre, DISSCO) and evaluated its association with risks of hospitalization and mortality, assessing its additional risk information to sociodemographic factors and HbA_1c_.

**Research design and methods:**

We used UK primary and secondary care data for 139 626 individuals with type 2 diabetes between 2007 and 2017, aged ≥35 years, and registered in general practices in England. The study cohort was randomly divided into a training cohort (n=111 748, 80%) to develop the severity tool and a validation cohort (n=27 878). We developed baseline and longitudinal severity scores using 34 diabetes-related domains. Cox regression models (adjusted for age, gender, ethnicity, deprivation, and HbA_1c_) were used for primary (all-cause mortality) and secondary (hospitalization due to any cause, diabetes, hypoglycemia, or cardiovascular disease or procedures) outcomes. Likelihood ratio (LR) tests were fitted to assess the significance of adding DISSCO to the sociodemographics and HbA_1c_ models.

**Results:**

A total of 139 626 patients registered in 400 general practices, aged 63±12 years were included, 45% of whom were women, 83% were White, and 18% were from deprived areas. The mean baseline severity score was 1.3±2.0. Overall, 27 362 (20%) people died and 99 951 (72%) had ≥1 hospitalization. In the training cohort, a one-unit increase in baseline DISSCO was associated with higher hazard of mortality (HR: 1.14, 95% CI 1.13 to 1.15, area under the receiver operating characteristics curve (AUROC)=0.76) and cardiovascular hospitalization (HR: 1.45, 95% CI 1.43 to 1.46, AUROC=0.73). The LR tests showed that adding DISSCO to sociodemographic variables significantly improved the predictive value of survival models, outperforming the added value of HbA_1c_ for all outcomes. Findings were consistent in the validation cohort.

**Conclusions:**

Higher levels of DISSCO are associated with higher risks for hospital admissions and mortality. The new severity score had higher predictive value than the proxy used in clinical practice, HbA_1c_. This reproducible algorithm can help practitioners stratify clinical care of patients with type 2 diabetes.

**Significance of this study**

**What is already known about this subject?**

- The prevalence of type 2 diabetes is rapidly increasing worldwide, with associated burdens of morbidity and excess mortality, but validated type 2 diabetes severity measures derived from real-world health records are lacking, as are applications of such measures in clinical practice, despite the importance of assessing diabetes severity being well recognized.

**What are the new findings?**

- The new DIabetes Severity SCOre (DISSCO), developed using 34 severity domains coded in routinely collected electronic health records, had overall better predictive value than glycated hemoglobin and showed that people with higher levels of the scores were at up to 45% significantly increased risk of hospital admission and death.
- DISSCO shows that diabetes-specific severity measures using electronic health records are feasible, with many applications directly relevant to clinical practice and risk stratification in populations.
- The methodology driven by routinely collected medical data is applicable to other chronic conditions managed in primary care setting.

**How might these results change the focus of research or clinical practice?**

- The results can inform risk stratification for people with type 2 diabetes based on disease severity to support clinicians in providing better self-care and efficient diabetes management in primary care.
- The methodology is applicable to other conditions using routinely collected medical data in future research.

## Introduction

Diabetes mellitus is a global epidemic with a rapidly increasing prevalence. The WHO placed diabetes as the seventh main cause of death in 2016.^1^ The cost of diabetes is estimated to be almost one-tenth of the total national healthcare budget in the UK and the USA.^2 3^ Type 2 diabetes affects nearly 90% of all people with diabetes,^4^ and leads to higher morbidity and mortality related to complications including vascular disease, renal failure, amputations and blindness, in comparison with people without diabetes.^5 6^

Despite the clinical importance of diabetes and its wider impact on healthcare, summary measures of diabetes severity and their relationship with clinical outcomes have not been widely considered. The ‘severity’ of a clinical condition is commonly defined via interlinked concepts related to the progression of the underlying processes of the disease.^7^ Increasing disease severity and subsequent development of associated complications lead to greater treatment complexity, healthcare resources utilization and impact on patients’ welfare. We sought to develop a new type 2 diabetes severity measure with key focus on disease complications and increased risks for adverse events and death. In current clinical practice, glycated hemoglobin (HbA_1c_) is used to summarize glycemic control over the preceding 3 months and might be used as a simple proxy for disease severity, guiding interventions and management. However, HbA_1c_ is a unidimensional measure, making it a poor predictor of adverse outcomes. Other generic tools to assess severity of long-term conditions exist, for example the Duke Severity of Illness Checklist,^8^ the Charlson Comorbidity Index,^9^ and the Elixhauser Comorbidity Index.^10^ However, the applicability of these tools to type 2 diabetes is unclear. Logically, a comorbidity-aware disease severity score developed around a driving, index condition may be more predictive than a general measure of comorbidity burden.

Our recent systematic review found that a few studies have assessed or quantified disease severity among individuals with type 2 diabetes and fewer had useful longitudinal measures.^11–14^ We identified the need for a valid and reliable tool for measuring severity of type 2 diabetes and that can serve as an actionable tool for therapeutic targets, as a covariate in epidemiological research, and for stratifying populations in order to inform resource allocation and support commissioning and public health programs for people with diabetes.^7^ Our study aimed to (1) develop a type 2 diabetes severity score (the DIabetes Severity SCOre, DISSCO), (2) evaluate the association and the added clinical utility of the severity score in predicting hospitalization and mortality outcomes beyond that achieved using models incorporating sociodemographic variables and HbA_1c_, and (3) validate DISSCO using a separate cohort (validation data set).

## Research design and methods

### Data source and patient population

We used the UK Clinical Practice Research Datalink (CPRD) GOLD. CPRD is one of the world’s largest electronic health record (EHR) databases providing detailed anonymized medical data and is representative of the UK population.^15 16^ CPRD provides data linkage to additional clinical data sets and disease registries.^17 18^ We used three data linkages: Hospital Episode Statistics (HES), Office for National Statistics (ONS) mortality data, and patient-level Index of Multiple Deprivation (IMD) quintiles 2015. The planned study design has been reported previously.^7^

A prevalent cohort of patients registered in general practices eligible for data linkage with at least one diagnostic code for type 2 diabetes from 1 March 2007 to 31 March 2017 and aged ≥35 years at diagnosis were identified. Patients who also had a code for type 1 diabetes in their entire data record or an indiscriminate gender were excluded. For each patient, index date was defined as the earliest diabetes diagnosis date. Each patient was followed up from the index date (t_0_) contributing to the survival time in years (t) until the earliest date of developing an outcome of interest, leaving the general practice, study end date (31 March 2017), or death. The final cohort of eligible patients was randomly split at patient level into training (80% of the cohort) and validation (20% of the cohort) data sets.

### Aim 1: diabetes severity assessment

#### Severity domains

Key clinically relevant indicators of type 2 diabetes severity (severity domains) were identified from a systematic review of studies that quantified type 2 diabetes severity.^14^ We also sought expert clinical opinion in cardiology, diabetes and primary care, supplementing the list of severity domains. HbA_1c_ and demographic variables were excluded from the severity domains. We identified 34 severity domains (online supplementary table S1).

10.1136/bmjdrc-2019-000962.supp1 Supplementary data

#### Calculation of severity scores

Using the defined severity domains, we calculated DISSCO using three different methods to assess the baseline and longitudinal levels of type 2 diabetes severity. The methods differed in the weights assigned to each diabetes severity domain when computing the overall severity score. The first and second methods were based on the binary and hierarchical classifications reported previously.^7^

The first method was a simple count (C) of the total number of severity domains present for an individual, out of 29, by assigning equal weighting (weight of 1) to all domains in the overall score (online supplementary table S1). In this method, 29 of the 34 domains were used by merging 8 domains into 3 domains.

The second method produced severity-weighted (SW) scores by assigning a weight to each of the 34 domains according to a hierarchy of increased severity based on clinical judgment as reported previously.^7^ As provided in online supplementary table S2, for example, a hierarchical weight of 1 was assigned to a transient ischemic attack (TIA) event, 2 to an event of carotid artery interventions, and 3 to a record of stroke to indicate the increasing severity of cerebrovascular events. For each patient, the overall SW score was the sum of the total domains’ hierarchical weights.

For the simple count and SW scores, we investigated the effects of varying the length of the pre-index (before diabetes diagnosis index) look-back window, defining the time period within which events had to occur for inclusion in the severity score calculation. We examined three lengths of look-back windows: unlimited (events at any time in the patient’s entire pre-index record), up to 10 years before index date, and up to 5 years before index date.

The third method was an exploratory analysis based on weighing the severity domains recorded before index date by proximity to index date, to generate ‘proximity-weighted’ (PW) score. This approach used a 5-year pre-index window, with any events prior to this window not being counted in the score (ie, weight of 0). Events within a year before the index year were assigned a weight of 1, 2 years a weight of 0.8, 3 years a weight of 0.6, 4 years a weight of 0.4, and 5 years a weight of 0.2.

Finally, to account for longitudinal severity that may have developed after the diagnosis of type 2 diabetes (post-index), we explored models incorporating severity domains recorded after the index date. Thus, we calculated ‘moving post-index severity scores’, allowing the window within which events could occur to range from 1 to 5 years after the index date, each coupled with the three look-back windows (online supplementary figure S1). For example, the 3-year post-index window coupled with the 10-year look-back window ranged from 7 years prior to the index year to 3 years afterwards.

In summary, we calculated 37 severity scores per patient as follows:

- Seven pre-index scores for computing overall severity using the three aforementioned methods:
  - A simple count (C) of domains score using the following windows:
    1. Unlimited look-back window (CU).
    2. 10-year look-back window (C10).
    3. 5-year look-back window (C5).
  - Severity-weighted (SW) score using:
    1. Unlimited look-back window (SWU).
    2. 10-year look-back window (SW10).
    3. 5-year look-back window (SW5).
  - Proximity-weighted (PW) score using the following:
    1. 5-year look-back window (PW).
- 30 post-index scores, based on combining each of windows 1–6 with post-index windows length of 1, 2, 3, 4, and 5 years.

### Aim 2: evaluating the added predictive value and clinical utility of the severity score

We evaluated the effect of adding the severity score by comparing with survival models that contain only age, gender, ethnicity, and IMD quintiles. We also investigated whether the inclusion of HbA_1c_ improved the prediction of outcomes by adjusting for baseline HbA_1c_ levels.

### Outcomes

The primary outcome was all-cause mortality. The secondary outcomes were future hospitalizations: any cause, due to cardiovascular (CV), diabetes (hypoglycemia-related hospitalizations; aggregated diabetic microvascular complications, foot ulcers, amputation, gangrene, hyperosmolar hyperglycemic state, and diabetic ketoacidosis), clustered CV and diabetes, or CV procedures (as coded by the OPCS Classification of Interventions and Procedures (OPCS-4). Cause-specific hospitalization was identified using the tenth revision International Classification of Diseases (ICD-10) code recorded in the first hospital admission after the index date. The hospitalization and death outcomes were identified using linked hospitalization admitted patient care (HES APC) data and the ONS mortality data, respectively.

### Statistical analysis

Patient characteristics are described by the data set purpose (training and validation cohorts) and by levels of baseline 10-year DISSCO (0, 1, 2, 3, 4, 5, and ≥6) and presented as mean±SD or count (%). Descriptive statistics of the developed severity scores are reported at baseline (index date) and over time (up to 5 years after index). Social deprivation levels were modeled as IMD quintiles (quintile 1 indicating the least deprived level and quintile 5 as the most deprived level). Patients with missing deprivation level were assigned to a sixth category, ‘Unknown’.

Baseline HbA_1c_ was based on the nearest test result recorded within 1 year before and 6 months after the index date. We found HbA_1c_ recording improved over time as the number of patients with HbA_1c_ recorded at baseline increased from 82% at baseline to 90% at the study end in both cohorts (online supplementary table S3). If there was no HbA_1c_ recorded during 1 year before to 6 months after index date, then baseline HbA_1c_ was deemed missing for that patient.

The relationships between the sociodemographic and clinical covariates, with and without the new severity score (simple count (C), SW, or PW), and the outcomes were evaluated using four Cox proportional hazards models, reporting HR (95% CI):

- Model 1: age, gender, deprivation, and ethnicity.
- Model 2: model 1 and severity score.
- Model 3: model 1 and HbA_1c_.
- Model 4: model 1, HbA_1c_ and severity score.

The PW score and moving post-index scores were only modeled with the primary outcome (all-cause mortality). Online supplementary tables S4 and S5 summarize the fitted Cox regression models. The level of deprivation was modeled using the least deprived as the referent group and for ethnicity variable, White was the referent group. Baseline HbA_1c_ was modeled using the International Federation of Clinical Chemistry (IFCC) unit of measure (mmol/mol) divided by 10 for a meaningful interpretation of the results on a 10-unit increase of HbA_1c_.

The Cox models included a severity score to evaluate the association with future outcomes’ prediction window (follow-up), that is, from the index date until censoring. The predictive value of all survival models was assessed using Gönen and Heller’s K concordance statistic (C-statistic), a measure of the area under the receiver operating characteristics (AUROC) curve for censored data. C-statistic ranges between 0 and 1, where a value of 0.5 indicates no predictive discrimination and a value close to 1 indicates an accurate model with high separation of subjects with different outcomes.^19^ Likelihood ratio (LR) tests were fitted to assess the statistical significance of adding DISSCO to the demographics and HbA_1c_ models in improving the models’ fit. The severity scores’ calibration was tested using three methods: Somer’s D; comparing the survival curves for a given risk group in the training and validation data sets; and predicting population-averaged survival probabilities and comparing the observed and predicted survival probabilities in several prognostic groups derived by the severity score’s cut points. The proportional hazards assumption was assessed using Schoenfeld residuals. Data were analyzed using Stata V.15.^20^ The study is reported in accordance with the RECORD (REporting of studies Conducted using Observational Routinely collected health Data) statement.^21^

### Aim 3: validation data set

All analyses were replicated in the validation data set.

### Patient and public involvement and engagement (PPIE)

People with type 2 diabetes were invited to a patient and public involvement and engagement event to seek their views on the included severity domains and the score calculation methods. They were also asked about the readability of a lay summary of the study and for advice about dissemination approaches for reporting the study results. The participants commented on the importance and the relevance of the study and the need to raise awareness on diabetes severity and the involvement of several body organs. Perceptions of severity domains varied among participants, where some participants did not identify that some conditions were related to their diabetes and its progression (such as declined renal function and foot problems), while other participants listed additional indicators such as rapid onset of tiredness and stress. The participants were more interested in the SW score approach and commented that it could include more intermediate domains, such as more stages between laser therapy and blindness, which we explained were often lacking in electronic data. The participants advised to disseminate the study results to patients and clinical audiences including general practices, diabetes centers, and lay audiences using social media.

## Results

### Patient population

A total of 139 626 eligible patients with type 2 diabetes were were included in the analysis. The training data set included 111 748 patients and the validation data set 27 878, with a mean (±SD) follow-up of 7.6 (±4.8) years. The mean age of the training and validation cohorts was 63.0 (±12.5) years and 63.1 (±12.6) years, respectively, of which 49 686 (45%) and 12 482 (45%) were women, respectively (table 1). Nearly 12.5% of people were censored due to transfer out of the general practice during the study (of whom 73% left the practice due to unknown reasons and 14% due to death).

**Table 1:** Baseline characteristics of the identified study cohort of type 2 diabetes, by full dataset and by training and validation cohortsdatasets

Characteristics	Full dataset	Training dataset	Validation dataset
Patient count, n (%)	139 626	111 748 (80)	27 878 (20)
Age, years±SD	63.0±12.5	63.0±12.5	63.1±12.6
Gender (female), n (%)	62 168 (44.5)	49 686 (44.5)	12 482 (44.8)
Number of general practices	400	400	396
Mean baseline HbA_1c_,% (mmol/mol±SD)*	7.8 (62±22)	7.8 (62±22)	7.8 (62±22)
Cases with HbA_1c_ data, n (%)	122 294 (88)	97 858 (88)	24 436 (88)
Ethnicity, n (%)
White	116 393 (83.4)	93 157 (83.4)	23 236 (83.3)
Non-White	11 024 (7.9)	8842 (7.9)	2182 (7.8)
Unknown	12 209 (8.7)	9749 (8.7)	2460 (8.8)
IMD quintiles, n (%)			
Quintile 1 (affluent)	26 930 (19.3)	21 437 (19.2)	5493 (19.7)
Quintile 2	29 534 (21.1)	23 580 (21.1)	5954 (21.4)
Quintile 3	29 539 (21.1)	23 742 (21.2)	5797 (20.8)
Quintile 4	27 883 (20.0)	22 341 (20.0)	5542 (19.9)
Quintile 5 (deprived)	25 641 (18.4)	20 571 (18.4)	5070 (18.2)
Unknown	99 (0.1)	77 (0.1)	22 (0.1)
Mean follow-up, years±SD	7.6±4.8	7.6±4.7	7.6±4.8
Region in England, n (%)			
North East	3202 (2.3)	2560 (2.3)	642 (2.3)
North West	24 115 (17.3)	19 412 (17.4)	4703 (16.9)
Yorkshire and the Humber	6036 (4.3)	4799 (4.3)	1237 (4.4)
East Midlands	4553 (3.3)	3660 (3.3)	893 (3.2)
West Midlands	17 838 (12.8)	14 252 (12.8)	3586 (12.9)
East of England	14 540 (10.4)	11 573 (10.4)	2967 (10.6)
South West	18 658 (13.4)	14 888 (13.3)	3770 (13.5)
South Central	15 617 (11.2)	12 441 (11.1)	3176 (11.4)
London	17 250 (12.4)	13 829 (12.4)	3421 (12.3)
South East Coast	17 817 (12.8)	14 334 (12.8)	3483 (12.5)

*Most recent measure within 1 year before or 6 months after index date.

HbA_1c_, glycated hemoglobin; IMD, Index of Multiple Deprivation.

Compared with individuals with 10-year pre-index simple count score (C10) of 0 (no severity domains at baseline), individuals with a score of ≥6 were older (72.0±9.5 vs 57.8±12.3), more likely to be male (67% vs 57%), and overall similar proportions were living in more deprived areas (21% vs 18%) (online supplementary table S6). CV domains were highly prevalent in those with severity score ≥6.

### Severity score (aim 1)

In the training data set, the simple count and SW scores differed in terms of mean and range by the time of look-back window, and increased over time. The pre-index 10-year count score (C10) ranged between 0 and 12 (online supplementary figure S2A), while the corresponding SW score (SW10) score ranged between 0 and 22 (online supplementary figure S2B and online supplementary table S7). The severity scores calculated using the 10-year look-back windows were very similar to that calculated using unlimited look-back windows. The 10-year look-back captured around 80% of all domains contributing to severity relative to the unlimited window. The PW score ranged between 0 and 7.6. The distribution of simple count and SW scores at pre-index (online supplementary figure S2) and post-index (online supplementary figures S3 and S4) windows showed similar trend overall.

### Survival analyses (aim 2)

Cox regression models were fitted to assess the relationship between severity score level and all-cause mortality and six future any cause and cause-specific hospitalizations events described. The estimated HRs for severity scores derived by the three different methods are not directly comparable, being based on different scales; therefore, AUROCs were used for comparison across models. Testing for proportional hazards indicated the assumptions held true as shown by selected figures representing each outcome presented in online supplementary figures S5 and S6.

#### Mortality outcome

**Figure 1:**
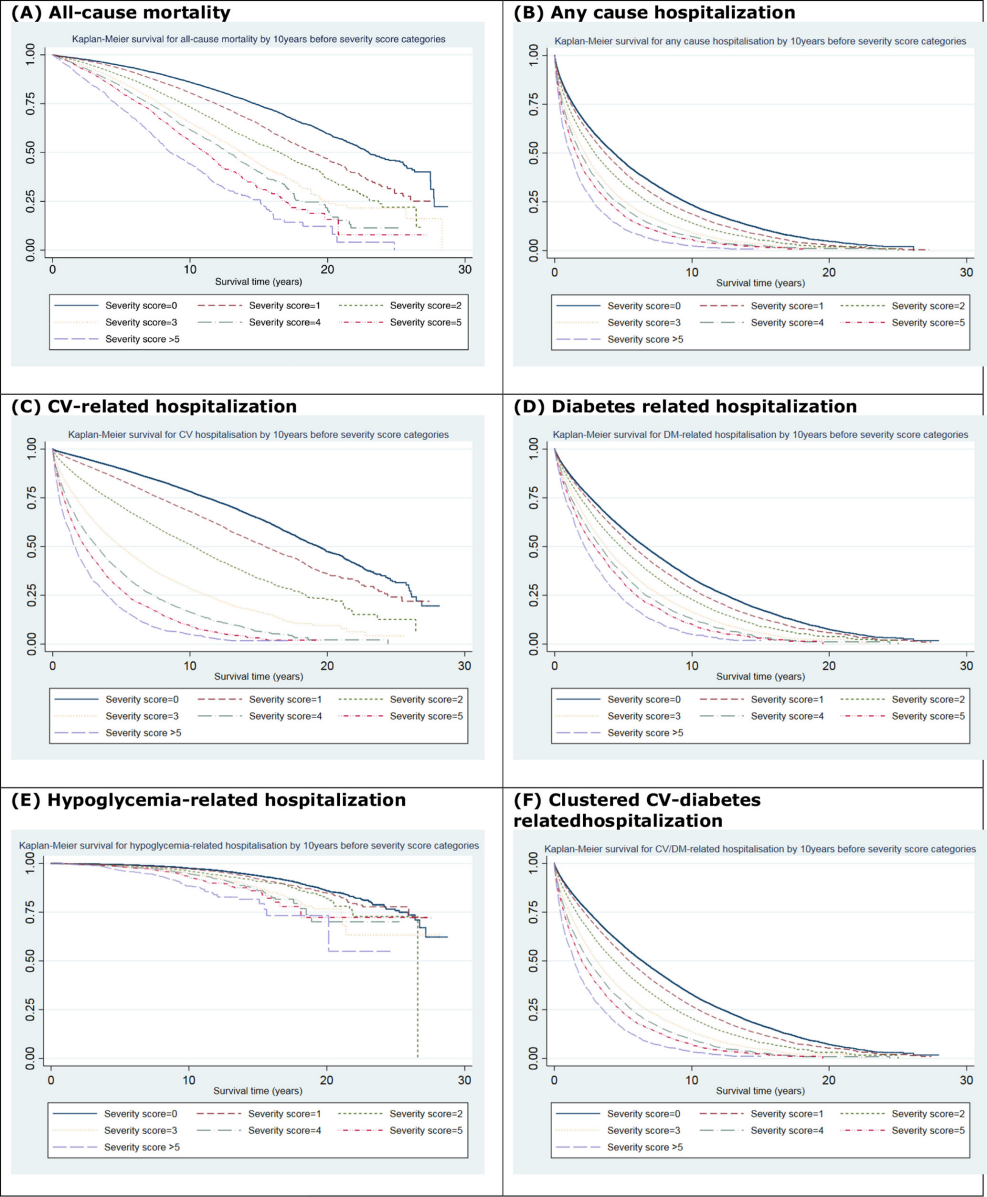
Kaplan-Meier plots for risk of adverse outcomes associated with 10-year (C10) severity score categories: (A) all-cause mortality; (B) any-cause hospitalization; (C) cardiovascular (CV)-related hospitalization; (D) diabetes-related hospitalization; (E) hypoglycemia-related hospitalization; and (F) clustered CV-diabetes related hospitalization—training data set.

In the training data set, a total of 21 969 (20% of patients) deaths occurred over 848 742 patient-years of follow-up. The results show that diabetes severity was positively associated with increasing risk for all-cause mortality (figure 1A). An increase of one-unit in the 10-year simple count (C10) score at the index year was associated with up to 14% (95% CI 13% to 15%, AUROC=0.76) higher risk for mortality when adjusted for demographics and HbA_1c_ (model 4; online supplementary table S8). Older patients and men were at a greater risk.

Simple count and SW scores measured over moving 1–5 years post-index windows (moving post-index scores) showed consistent direction of association as observed with corresponding pre-index scores, indicating the association between a one-unit increase in post-index severity and risk of death (online supplementary tables S9 and S10). The strength of association remained statistically significant at 5 years after index date and a look-back window of up to 10 years (C10) (adjusted HR: 1.16, 95% CI 1.15 to 1.17, AUROC=0.75). Similar overall trends were observed with post-index SW scores. Similar estimate was obtained using the PW score in the fully adjusted model (model 4) (HR: 1.18, 95% CI 1.16 to 1.20, AUROC=0.75).

#### Hospitalization outcomes

Over the follow-up period, a total of 79 974 (72% of patients) hospitalization events of any cause occurred over 409 406 patient-years of follow-up (training data set). These included 37 134 (33%) CV-related hospitalizations, 69 120 (62%) diabetes-related hospitalizations, 3268 (3%) hypoglycemia hospitalizations, 71 505 (64%) clustered CV and diabetes-related hospitalizations, and 5593 (5%) CV procedures. Increasing diabetes severity at index year showed a greater risk for future hospitalizations (figure 1B). A one-unit increase in C10 score was associated with 9% (95% CI 8% to 9%, AUROC=0.63) significantly higher risk for hospital admission of any cause (online supplementary table S11). Severity remained strongly associated with hospitalization outcomes after further adjusting for HbA_1c_ levels.

In the fully adjusted model (model 4), a one-unit higher 10-year simple count score was associated with a 45% (95% CI 43% to 46%) greater risk of being hospitalized for cardiovascular disease (AUROC=0.73) (online supplementary table S12 and figure 1C). The corresponding SW10 score was associated with a 24% risk (95% CI 23% to 24%, AUROC=0.72).

Individuals with a one-unit higher C10 score were also at greater risk for other cause-specific hospitalization admissions (figure 1D–F): diabetes-related hospitalizations (adjusted HR: 1.10, 95% CI 1.09 to 1.10, AUROC=0.63) (online supplementary table S13), hypoglycemia-related hospitalizations (adjusted HR: 1.15, 95% CI 1.12 to 1.18, AUROC=0.72) (online supplementary table S14), clustered CV/diabetes-related events (adjusted HR: 1.14, 95% CI 1.13 to 1.14, AUROC=0.64) (online supplementary table S15), and CV interventions (adjusted HR: 1.33, 95% CI 1.31 to 1.35, AUROC=0.69) (online supplementary table S16).

Assessing the predictive value of the developed severity score (DISSCO) showed its additive and improved predictive value. The AUROC for models with and without severity score shows that adding DISSCO improved the model discrimination for all measured outcomes, as observed in model 2 versus model 1 and in model 4 versus model 3 (table 2 and online supplementary figure S7 and S8). For CV hospitalization, model 3 had an AUROC of 0.71, which increased to an AUROC of 0.73 when C10 score was added (model 4), while modest improvements were observed for all-cause mortality and diabetes-related hospitalizations, but all were significant (p<0.001) as shown by LR tests. The performed calibration tests showed good calibration of the severity scores (online supplementary table S17; figure 1 vs online supplementary figures S9, and S10).

**Table 2:** AUROC for predicting the risk of adverse outcomes in survival models with clinical and sociodemographic variables with and without simple count and severity-weighted scores (training data set)

	All-cause mortality	Any-cause hospitalization	CV hospitalization	Diabetes-related hospitalization	Clustered CV/diabetes-related hospitalization	Hypoglycemia hospitalization	CV procedures
Model 1	0.7528	0.6231	0.7033	0.6249	0.6329	0.6997	0.6665
Model 2 CU score (SWU score)	0.7570 (0.7556)	0.6298 (0.6299)	0.7281 (0.7169)	0.6324 (0.6323)	0.6445 (0.6433)	0.7052 (0.7056)	0.6858 (0.6778)
Model 2 C10 score (SW10 score)	0.7563 (0.7553)	0.6288 (0.6291)	0.7267 (0.7162)	0.6312 (0.6315)	0.6427 (0.6422)	0.7042 (0.7047)	0.6850 (0.6773)
Model 2 C5 score (SW5 score)	0.7553 (0.7548)	0.6274 (0.6280)	0.7226 (0.7138)	0.6297 (0.6302)	0.6404 (0.6404)	0.7026 (0.7036)	0.6833 (0.6759)
Model 3	0.7529	0.6230	0.7057	0.6254	0.6329	0.7104	0.6723
Model 4 CU score (SWU score)	0.7570 (0.7554)	0.6293 (0.6293)	0.7298 (0.7185)	0.6327 (0.6325)	0.6442 (0.6431)	0.7162 (0.7163)	0.6903 (0.6825)
Model 4 C10 score (SW10 score)	0.7563 (0.7552)	0.6283 (0.6286)	0.7288 (0.7179)	0.6316 (0.6317)	0.6425 (0.6420)	0.7151 (0.7155)	0.6901 (0.6823)
Model 4 C5 score (SW5 score)	0.7553 (0.7547)	0.6270 (0.6276)	0.7247 (0.7156)	0.6302 (0.6306)	0.6404 (0.6403)	0.7136 (0.7144)	0.6885 (0.6809)

Model 1: age, gender, deprivation, and ethnicity.

Model 2: model 1+ severity score.

Model 3: age, gender, deprivation, ethnicity, and HbA_1c_ (glycated hemoglobin).

Model 4: model 3 + severity score.

Simple count (C) score measured using unlimited (CU), 10-year (C10), and 5-year (C5) look-back windows.

Severity-weighted (SW) score measured using unlimited (SWU), 10-year (SW10), and 5-year (SW5) look-back windows.

AUROC, area under the receiver operating characteristics curve; CV, cardiovascular.

### Validation data set (aim 3)

The validation data set included 27 878 patients (table 1). The regression analyses conducted in the validation data set (online supplementary tables S18–S22, figure S5) resulted in consistent findings with those reported with the training data set. A summary of the findings in the training and validation data sets is presented in table 3.

**Table 3:** HR (95% CI) for risk of primary and secondary outcomes associated with baseline severity score using training and validation data sets

	Training data set HR (95% CI)	Validation data set HR (95% CI)
All-cause mortality	1.14 (1.13 to 1.15)	1.16 (1.14 to 1.18)
	AUROC=0.7563	AUROC=0.7617
All-cause hospitalization	1.09 (1.08 to 1.09)	1.09 (1.08 to 1.11)
	AUROC=0.6283	AUROC=0.6320
CV-related hospitalization	1.45 (1.43 to 1.46)	1.44 (1.42 to 1.46)
	AUROC=0.7288	AUROC=0.7326
Diabetes-related hospitalization	1.10 (1.09 to 1.10)	1.10 (1.08 to 1.11)
	AUROC=0.6316	AUROC=0.6311
Clustered CV-related or diabetes-related hospitalization	1.14 (1.13 to 1.14)	1.14 (1.13 to 1.15)
	AUROC=0.6425	AUROC=0.6439
Hypoglycemia-related hospitalization	1.15 (1.12 to 1.18)	1.18 (1.12 to 1.24)
	AUROC=0.7151	AUROC=0.7225

Data based on model 4 (adjusted for baseline C10 score, age at index, gender, HbA_1c_, deprivation, and ethnicity) (restricted to individuals with non-missing baseline HbA_1c_; training data set n=97 858 and validation data set n=25 099).

AUROC, area under the receiver operating characteristics curve; CV, cardiovascular; HbA_1c_, glycated hemoglobin.

## Discussion

### Main findings

We present a contemporary scoring system using EHRs to grade type 2 diabetes severity in a large primary care cohort. Our findings prove the concept that longitudinal EHRs and linked administrative data can be used to develop type 2 diabetes severity score that is useful for predicting key outcomes, with a methodology applicable to other chronic conditions. The developed baseline and longitudinal severity scores (DISSCO) provided important prognostic information for hospitalization and mortality events. DISSCO improved the predictive value of models for all measured outcomes when added to basic sociodemographic variables and also performed better than adding HbA_1c_ levels. This indicates the value of the included severity domains by mapping to the measured outcomes. The predictive value of pre-index and post-index simple count scores was slightly higher than the SW score. Results were very similar for the 10-year and unlimited window models. In actual practice systems, patient records can stretch back many decades. We therefore recommend using a window of 10 years to reduce confounding with varying record lengths and less reliable data.

### Comparison with other studies

Our recent systematic review showed some development of diabetes-specific severity scores, mainly using EHRs, in several countries but not in the UK.^14^ Prior studies used either continuous or categorical grading systems based on diabetes-related complications and glycemic indicators, mainly HbA_1c_. In one study, the severity of type 2 diabetes in 300 individuals was categorized into four levels using an automated algorithm based on two domains: insulin use and the presence of diabetes complications.^12^ Another US study assessed severity using two methods involving a number of severity indicators, including diabetes complications and laboratory data. The latter study reported greater risks of hospitalization and death (over a total of 14 166 patients-years) with increased type 2 diabetes severity, consistent with our findings.^13^

Despite the knowledge added by these previous models, some studies were limited by design (such as small sample sizes ranging from 65 to 4229) or the type of included severity indicators. Importantly, none of the studies compared the severity measures with HbA_1c_, and only a few examined the prospective application of the severity measure to serve as an actionable clinical tool.^22 23^ In comparison, we included a much larger type 2 diabetes cohort (n=139 626) over a longer follow-up (up to 848 742 patient-years) using multiple approaches to assess severity and investigated its association with cause-specific hospitalization. Importantly, table 2 shows the superiority of the added predictive value of DISSCO (model 2) over HbA_1c_ (model 3) when added to model 1.

### Implications and clinical relevance

Assessing disease severity for a highly prevalent chronic condition such as type 2 diabetes can have clinical validity beyond that achieved using more traditional approaches (demographic and clinical variables). Well-defined disease severity measurement based on readily collected data has important implications on targeted patient care, utilization of healthcare services and service planning. The focus of this study was on type 2 diabetes; however, future studies are needed to assess the severity of type 1 diabetes, but separately from other forms due to its distinctive phenotype, and epidemiological and pathophysiological differences compared with type 2 diabetes. Our study adds to current knowledge by offering a contemporary and validated type 2 diabetes severity measure driven by data collected in primary care. Given that diabetes is mainly managed in primary care settings,^24–28^ a primary care-derived measure has broad applicability especially when it predicts key outcomes well, as DISSCO does. The proposed algorithm can be initially introduced as a paper-based version (online supplementary table S23) to enable clinicians to calculate baseline and longitudinal severity scores and discuss them with the patient, that is, as a risk stratification tool to identify patients with type 2 diabetes at higher risk for adverse outcomes, which informs advanced decisions with patients and their carers. Future implementation studies are needed to develop DISSCO into an accessible software that can be incorporated in EHRs, such as QRISK and FRAX scores,^29 30^ as a practical actionable tool to stratify patients with diabetes by severity of their condition for better self-care and efficient diabetes management in primary care. Additionally, our approach is applicable to most long-term conditions. While acknowledging the different coding systems and other operational/technical differences, some international primary care data sets and diabetes registries, such as the Swedish National Diabetes Register,^31^ the Canadian Primary Care Sentinel Surveillance Network,^32^ and the Diabetes Collaborative Registry,^33^ include the majority of our severity domains, indicating the score’s reproducibility and potential generalizability in non-UK health systems.

### Strengths and limitations of the study

Our study has several strengths. First, to the best of our knowledge, it is the first large study to measure the severity of diabetes using UK (England) data. Second, we used high-quality clinical primary and secondary care data, and we developed a contemporary severity measure using data collected in routine general practice visits to indicate the potential of our measure as a practical tool in primary care, with a methodology applicable to type 2 diabetes and also to other long-term conditions. Third, we developed and validated the new severity tool comparing three approaches in large well-defined study cohorts of people with type 2 diabetes. Fourth, we triangulated expert opinion, evidence from literature and database analysis, which resulted in us including more type 2 diabetes-related severity domains compared with previous studies. Fifth, we found an added predictive value of our severity score versus HbA_1c_, which is the mainstay of current clinical decision-making around type 2 diabetes management and monitoring.

There are some limitations to our study. First, we may have missed other severity indicators not recorded in the primary care data. However, using routinely coded data would facilitate automation of disease severity assessment and make our model widely applicable. Second, one of the drawbacks of using comorbidities for assessing severity is their broad definition, which may include conditions not related to diabetes—we used multidisciplinary expert knowledge to maximize the relevance of clinical concepts and codes of the domains included. Third, some of the severity indicators are related to increasing age and deprivation (and possible other covariates), and therefore adjusting for these covariates may have attenuated the risk of outcomes associated with disease severity. Fourth, the lower discrimination observed with the clustered outcome is likely due to an artifact of the way secondary care data are recorded, maybe resulting in the cause of diabetes hospitalization not being added to the record or not added as the main cause for that admission. Fifth, our method might be limited being it health system-specific and more relevant to countries with automated and comprehensive EHRs. However, DISSCO has potential generalizability in non-UK countries as it includes coded clinical data widely found in EHRs adopted for primary care mainly for people with type 2 diabetes, including in countries lacking established EHRs. Finally, external validation of DISSCO in an independent population and a clinical decision support system is needed before reporting its full clinical utility and implications. This can be addressed in a future study.

## Conclusion

The prevalence of type 2 diabetes has increased steeply worldwide. This study provides a new type 2 diabetes severity score, DISSCO, which considerably improves the accuracy of predicting hospitalization and death using EHRs routinely collected on type 2 diabetes in primary care. DISSCO has higher predictive value than HbA_1c_ levels on all examined clinical outcomes. More generally, we show an overall similar predictive value of incorporating hierarchies of comorbidity severity in models or counts of complications. Disease-specific severity measures using EHRs are feasible, and this is directly relevant to clinical practice and risk stratification, indicating the applicability of big data in the direction of precision medicine. Such disease-specific severity measures can serve as an actionable tool for providing therapeutic targets, have applications in medical research, and have wider implications on individual patient and population healthcare.
