# Supplementary material for: Development and validation of the DIabetes Severity SCOre (DISSCO) in 139 626 individuals with type 2 diabetes: a retrospective cohort study
Source: BMJ Open Diabetes Res Care. 2020 May 7;8(1):e000962. doi: 10.1136/bmjdrc-2019-000962 (PMC7228474; doi:10.1136/bmjdrc-2019-000962)

Supplementary data to "Development and validation of the DIabetes Severity SCOre (DISSCO)". Zghebi et al.

Supplementary data

Zghebi SS, *et al. BMJ Open Diab Res Care* 2020; 8:e000962. doi: 10.1136/bmjdrc-2019-000962

Supplementary data to "Development and validation of the DIabetes Severity SCOre (DISSCO)". Zghebi et al.

Table S1 List of 34 severity domains

	Severity domain	Hierarchical domain	Binary domain
Diabetes-related complications and renal disease domains	1. **Diabetic neuropathy**	√	Combined with 2, 5, 8
	2. **Foot ulcers and Charcot foot**	√	Combined with 1, 5, 8
	3. **Gangrene**	√	√
	4. **Amputation**	√	√
	5. **Diabetic retinopathy**	√	Combined with 1, 2, 8
	6. **Laser therapy (including laser photocoagulation)**	√	√
	7. **Low vision, blindness**	√	√
	8. **Diabetic nephropathy**	√	Combined with 1,2, 5
	9. **Albuminuria, proteinuria**	√	√
	10. **ESRD (end stage renal disease): kidney transplant and dialysis**	√	√
	11. **Hypoglycaemia**	√	√
	12. **Diabetic ketoacidosis (DKA)**	√	√
	13. **Hyperosmolar hyperglycaemic state (HHS)**	√	√
Cerebrovascular and cardiovascular disease, risk factors and interventions domains	14. **Hypertension**	√	√
	15. **Hyperlipidaemia**	√	√
	16. **Stable angina**	√	√
	17. **Myocardial infarction (MI), acute coronary syndrome (ACS), unstable angina**	√	√
	18. **Heart valve disease**	√	√
	19. **Heart valve interventions**	√	√
	20. **Endocarditis**	√	√
	21. **Coronary artery interventions (percutaneous coronary intervention (PCI)/ percutaneous transluminal coronary angioplasty (PTCA), coronary artery bypass graft (CABG)**	√	Combined with 33
	22. **Cardiac arrest (ventricular tachycardia/fibrillation (VT/VF), Asystole)**	√	√
	23. **Atrial fibrillation (AF), supraventricular tachycardia (SVT)**	√	√
	24. **Pacemaker**	√	√
	25. **Heart failure (HF)**	√	√
	26. **Defibrillator, implantable cardioverter defibrillator (ICD), Cardiac resynchronization therapy (CRT) use**	√	√
	27. **Peripheral vascular disease (PVD)**	√	√
	28. **Cardiomyopathy**	√	√
	29. **Pericardial disease**	√	√
	30. **Myocarditis**	√	√
	31. **Coronary heart disease (CHD)**	√	√
	32. **Transient ischemic attack (TIA)**	√	Combined with 34
	33. **Carotid artery events, stenting and bypass interventions**	√	Combined with 21
	34. **Stroke**	√	Combined with 32

Zghebi SS, *et al. BMJ Open Diab Res Care* 2020; 8:e000962. doi: 10.1136/bmjdrc-2019-000962

Supplementary data to "Development and validation of the DIabetes Severity SCOre (DISSCO)". Zghebi et al.

Table S2 Assigned hierarchical weights

	Severity domain	Hierarchical weight
Diabetes-related complications and renal disease domains	1. **Diabetic neuropathy**	1
	2. **Foot ulcers and Charcot foot**	1
	3. **Gangrene**	2
	4. **Amputation**	3
	5. **Diabetic retinopathy**	1
	6. **Laser therapy (including laser photocoagulation)**	2
	7. **Low vision, blindness**	2
	8. **Diabetic nephropathy**	1
	9. **Albuminuria, proteinuria**	2
	10. **ESRD (end stage renal disease): kidney transplant and dialysis**	3
	11. **Hypoglycaemia**	1
	12. **Diabetic ketoacidosis (DKA)**	2
	13. **Hyperosmolar hyperglycaemic state (HHS)**	3
Cerebrovascular and cardiovascular disease, risk factors and interventions domains	14. **Hypertension**	0.5
	15. **Hyperlipidaemia**	0.5
	16. **Stable angina**	1
	17. **Myocardial infarction (MI), acute coronary syndrome (ACS), unstable angina**	2
	18. **Heart valve disease**	1
	19. **Heart valve interventions**	2
	20. **Endocarditis**	2
	21. **Coronary artery interventions (percutaneous coronary intervention (PCI)/ percutaneous transluminal coronary angioplasty (PTCA), coronary artery bypass graft (CABG))**	2
	22. **Cardiac arrest (ventricular tachycardia/fibrillation (VT/VF), Asystole)**	3
	23. **Atrial fibrillation (AF), supraventricular tachycardia (SVT)**	1
	24. **Pacemaker**	2
	25. **Heart failure (HF)**	3
	26. **Use of defibrillator, implantable cardioverter defibrillator (ICD), Cardiac resynchronization therapy (CRT)**	4
	27. **Peripheral vascular disease (PVD)**	1
	28. **Cardiomyopathy**	1
	29. **Pericardial disease**	1
	30. **Myocarditis**	1
	31. **Coronary heart disease (CHD)**	1
	32. **Transient ischemic attack (TIA)**	2
	33. **Carotid artery events, stenting and bypass interventions**	3
	34. **Stroke**	4

Supplementary data to "Development and validation of the DIabetes Severity SCOre (DISSCO)". Zghebi et al.

Table S3 Number of type 2 diabetes cases (%) by annual bins with baseline HbA_{1c} recorded within one year before and six months post-index (training and validation datasets)

	Up to 03/2008	Up to 03/2009	Up to 03/2010	Up to 03/2011	Up to 03/2012	Up to 03/2013	Up to 03/2014	Up to 03/2015	Up to 03/2016	Up to 03/2017
**Training dataset**										
**Total number of cases**	56,101	60,459	65,141	69,259	72,740	76,391	79,395	80,433	80,485	79,649
**Number (%) of cases with recorded HbA_{1c}**	45,940 (82%)	50,445 (83%)	55,231 (85%)	59,485 (86%)	63,085 (87%)	66,989 (88%)	70,198 (88%)	71,554 (89%)	72,068 (90%)	71,551 (90%)
**Validation dataset**										
**Total number of cases**	14,021	15,064	16,270	17,287	18,172	19,213	19,893	20,075	20,110	19,917
**Number (%) of cases with recorded HbA_{1c}**	11,489 (82%)	12,586 (84%)	13,801 (85%)	14,845 (86%)	15,759 (87%)	16,834 (88%)	17,590 (88%)	17,832 (89%)	17,973 (89%)	17,870 (90%)

Rationale for the chosen window to measure baseline HbA_{1c}: The one year before and six months after index date window was decided after examining the longitudinal patterns of recorded HbA_{1c} data and the number of cases with missing HbA_{1c} data in different windows before and after index date. Given the relevance of HbA_{1c} to our developed severity measure, we refrained from assigning individuals with missing HbA_{1c} to an "Unknown" category and preferred to supplement missing HbA_{1c} data by extending to measurements recorded within 6 months of post-index window i.e. using the closest measure recorded within a limited interval (one year before and six months after index date) instead. This approach can be justified by available data indicating that HbA_{1c} is fairly stable in people with type 2 diabetes, and that some cases were not first diagnosed with type 2 diabetes as a result of an HbA_{1c} testing (especially for early year cases and before HbA_{1c} test was approved for diabetes diagnosis in 2011 (BMJ 2012;345:e729), cases diagnosed due to opportunistic screening or while managing another health condition. But we would expect HbA_{1c} tests to be recorded afterwards, ideally, within the first 3-6 months of diabetes diagnosis.

Zghebi SS, *et al. BMJ Open Diab Res Care* 2020; 8:e000962. doi: 10.1136/bmjdrc-2019-000962

Supplementary data to "Development and validation of the DIabetes Severity SCOre (DISSCO)". Zghebi et al.

Table S4 Cox models using pre-index scores. A total of 100 models fitted using pre-index windows for primary and secondary outcomes.

		All-cause death	Any cause hospitalisation	CV hospitalisation	Diabetes-related hospitalisation	Clustered CV- or Diabetes-related hospitalisation	Hypoglycaemia-related hospitalisation	CV procedu-re
	Model 1 (Age, gender, ethnicity, and IMD)	√	√	√	√	√	√	√
Model 2	CU score +model 1	√	√	√	√	√	√	√
	C10 score +model 1	√	√	√	√	√	√	√
	C5 score +model 1	√	√	√	√	√	√	√
	SWU score +model 1	√	√	√	√	√	√	√
	SW10 score +model 1	√	√	√	√	√	√	√
	SW5 score +model 1	√	√	√	√	√	√	√
	PW score +model 1	√	-	-	-	-	-	-
	Model 3 (Age, gender, ethnicity, and IMD, HbA_{1c})	√	√	√	√	√	√	√
Model 4	CU score +model 3	√	√	√	√	√	√	√
	C10 score +model 3	√	√	√	√	√	√	√
	C5 score +model 3	√	√	√	√	√	√	√
	SWU score +model 3	√	√	√	√	√	√	√
	SW10 score +model 3	√	√	√	√	√	√	√
	SW5 score +model 3	√	√	√	√	√	√	√
	PW score +model 3	√	-	-	-	-	-	-

Zghebi SS, *et al. BMJ Open Diab Res Care* 2020; 8:e000962. doi: 10.1136/bmjdrc-2019-000962

Supplementary data to "Development and validation of the DIabetes Severity SCOre (DISSCO)". Zghebi et al.

Table S5 Cox models using post-index scores. A total of 70 Cox models fitted using 1-5 year moving post-index windows for all-cause mortality. *models were limited to patients contributing to each post-index window i.e. not censored.

		Index+1 year	Index+2 years	Index+3 years	Index+4 years	Index+5 years
	Model 1 (Age, gender, ethnicity, and IMD)*	√	√	√	√	√
Model 2	+CU +model 1	√	√	√	√	√
	+C10+model 1	√	√	√	√	√
	+C5+model 1	√	√	√	√	√
	+SWU+model 1	√	√	√	√	√
	+SW10+model 1	√	√	√	√	√
	+SW5+model 1	√	√	√	√	√
	Model 3 (Age, gender, ethnicity, IMD, and HbA_{1c})*	√	√	√	√	√
Model 4	+CU +model 3	√	√	√	√	√
	+C10+model 3	√	√	√	√	√
	+C5+model 3	√	√	√	√	√
	+SWU+model 3	√	√	√	√	√
	+SW10+model 3	√	√	√	√	√
	+SW5+model 3	√	√	√	√	√

Zghebi SS, *et al. BMJ Open Diab Res Care* 2020; 8:e000962. doi: 10.1136/bmjdrc-2019-000962

Supplementary data to "Development and validation of the DIabetes Severity SCOre (DISSCO)". Zghebi et al.

Table S6 Baseline characteristics of the identified type 2 diabetes study cohort by categories of the 10-year simple count severity score (C10) - training dataset

	Severity score= 0	Severity score= 1	Severity score= 2	Severity score= 3	Severity score= 4	Severity score= 5	Severity score ≥6
Patient count N(%)	38,152 (34.1)	35,845 (32.1)	19,075 (17.1)	9,405 (8.4)	5,175 (4.6)	2,532 (2.3)	1,564 (1.4)
Age,(years)±SD	57.8 ±12.3	63.2 ±11.9	66.5 ±11.4	69.1±10.9	70.8 ±10.6	70.8±10.0	72.0 ±9.5
Gender (Female) N(%)	16,498 (43.2)	16,955 (47.3)	8,935 (46.8)	3,887 (41.3)	1,972 (38.1)	921 (36.4)	518 (34.1)
Number of general practices	397	393	398	383	369	354	329
Mean baseline HbA_{1c} (mmol/mol)[&] ±SD<br>N(%) with HbA_{1c} data	68 ±25<br>32,612 (86%)	61 ±21<br>31,573 (88%)	58 ± 20<br>17,056 (89%)	58 ±19<br>8,312 (88%)	57± 18<br>4,625 (89%)	57 ±18<br>2,270 (90%)	58 ±18<br>1,410 (90%)
Ethnicity, N(%)							
• White	30,077 (78.8)	29,526 (82.4)	16,386 (85.9)	8,518 (90.6)	4,783 (92.4)	2,391 (94.4)	1,476 (94.4)
• Non-White	3,790 (10)	2,826 (7.9)	1,265 (6.6)	532 (5.7)	257 (5.0)	98 (3.9)	74 (4.7)
• Unknown	4,285 (11.2)	3,493 (9.7)	1,424 (7.5)	355 (3.8)	135 (2.6)	43 (1.7)	14 (0.9)
IMD quintiles, N(%)							
• Q1 (Affluent)	7,400 (19.4)	7,115 (19.9)	3,635 (19.1)	1,678 (17.8)	930 (18.0)	444 (17.5)	235 (15.0)
• Q2	7,889 (20.7)	7,646 (21.3)	4,139 (21.7)	1,998 (21.2)	1,043 (20.1)	525 (20.7)	340 (21.7)
• Q3	8,039 (21.0)	7,736 (21.6)	4,010 (21.0)	2,022 (21.5)	1,043 (20.1)	549 (21.7)	343 (21.9)
• Q4	7,813 (20.5)	6,929 (19.3)	3,807 (20.0)	1,902 (20.2)	1,066 (20.6)	505 (19.9)	319 (20.4)
• Q5 (Deprived)	6,986 (18.3)	6,403 (17.9)	3,463 (18.1)	1,800 (19.1)	1,088 (21.0)	509 (20.1)	327 (20.9)
• Unknown	25 (0.1)	16 (0.0)	21 (0.10)	5 (0.1)	5 (0.1)	**	**
Mean follow-up (yrs)±SD	8.05±5.1	7.6±4.6	7.3±4.5	7.2±4.4	7.1±4.4	6.9±4.2	6.2±4.1
Severity domains, N(%)<br>1. Hyperlipidaemia	0.0 (0)	4,607 (12.9)	7,521 (39.4)	3,381 (35.9)	2,528 (48.9)	1,338 (52.8)	989 (63.2)
2. Hypertension	0.0 (0)	25,574 (71.3)	14,715 (77)	6,674 (71.0)	4,225 (81.6)	1,970 (77.8)	1,272 (81.3)
3. Stable angina	0.0 (0)	0.0 (0)	979 (5.1)	2,466 (26.2)	2,351 (45.4)	1,499 (59.2)	1,075 (68.7)
4. Cardiac arrest	0.0 (0)	<5	15 (0.08)	30 (0.3)	51 (1.0)	37 (1.5)	119 (7.6)
5. AF/SVT	0.0 (0)	844 (2.4)	1,654 (8.7)	1,522 (16.2)	1,117 (21.6)	714 (28.2)	689 (44.1)
6. PVD	0.0 (0)	303 (0.9)	566 (3.0)	572 (6.1)	504 (9.7)	351 (13.9)	409 (26.2)
7. MI/ACS/unstable angina	0.0 (0)	0 (0.00)	712 (3.7)	1,429 (15.2)	1,539 (29.7)	1,169 (46.2)	907 (58.0)
8. CHD	0.0 (0)	1,792 (5.0)	6,908 (36.2)	6,887 (73.2)	4,596 (88.8)	2,424 (95.7)	1,529 (97.8)
9. Heart valve disease	0.0 (0)	5 (0.01)	14 (0.07)	20 (0.2)	31 (0.6)	16 (0.63)	24 (1.5)
10. Endocarditis	0.0 (0)	5 (0.01)	8 (0.04)	9 (0.1)	19 (0.4)	19 (0.75)	27 (1.7)
11. Myocarditis	0.0 (0)	<5	<5	<5	<5	<5	<5
12. Cardiomyopathy	0.0 (0)	19 (0.05)	45 (0.24)	87 (0.9)	85 (1.6)	63 (2.49)	53 (3.4)
13. Pericardial disease	0.0 (0)	22 (0.06)	22 (0.12)	30 (0.3)	22 (0.4)	17 (0.67)	19 (1.2)
14. Ventricular tachycardia/fibrillation	0.0 (0)	<5	10 (0.05)	15 (0.2)	30 (0.58)	23 (0.91)	88 (5.6)
15. CHF	0.0 (0)	265 (0.7)	662 (3.5)	893 (9.5)	876 (16.9)	699 (27.6)	734 (46.9)

Zghebi SS, *et al. BMJ Open Diab Res Care* 2020; 8:e000962. doi: 10.1136/bmjdrc-2019-000962

Supplementary data to "Development and validation of the DIabetes Severity SCOre (DISSCO)". Zghebi et al.

	**Severity score= 0**	**Severity score= 1**	**Severity score= 2**	**Severity score= 3**	**Severity score= 4**	**Severity score= 5**	**Severity score ≥6**
16. CV procedures	0.0 (0)	9 (0.03)	260 (1.4)	968 (10.3)	1,239 (23.9)	1,043 (41.2)	807 (51.6)
17. TIA/Stroke	0.0 (0)	851 (2.4)	1,993 (10.4)	1,470 (15.6)	922 (17.8)	519 (20.5)	470 (30.1)
18. Pacemaker	0.0 (0)	79 (0.2)	162 (0.8)	173 (1.8)	167 (3.2)	164 (6.5)	221 (14.1)
19. Defibrillator use	0.0 (0)	0 (0.00)	<5	7 (0.07)	10 (0.2)	19 (0.8)	56 (3.6)
20. Proteinuria, albuminuria	0.0 (0)	244 (0.7)	598 (3.1)	453 (4.8)	264 (5.1)	164 (6.5)	178 (11.4)
21. ESRD	0.0 (0)	18 (0.05)	56 (0.3)	41 (0.4)	23 (0.4)	14 (0.6)	16 (1.0)
22. Microvascular complications	0.0 (0)	680 (1.9)	881 (4.6)	611 (6.5)	339 (6.6)	209 (8.3)	228 (14.6)
23. Blindness, low vision	0.0 (0)	302 (0.8)	405 (2.1)	310 (3.3)	210 (4.1)	118 (4.7)	124 (7.9)
24. Laser therapy	0.0 (0)	13 (0.04)	28 (0.2)	31 (0.3)	22 (0.4)	6 (0.2)	13 (0.8)
25. Gangrene	0.0 (0)	5 (0.01)	5 (0.03)	<5	7 (0.1)	<5	6 (0.4)
26. Amputation	0.0 (0)	82 (0.2)	67 (0.4)	52 (0.6)	50 (1.0)	25 (1.0)	56 (3.6)
27. Hypoglycemia	0.0 (0)	74 (0.2)	86 (0.5)	65 (0.7)	46 (0.89)	31 (1.2)	49 (3.1)
28. Hyperosmolar hyperglycaemic state	0.0 (0)	17 (0.05)	18 (0.1)	6 (0.06)	7 (0.1)	<5	0.0 (0)
29. Diabetic ketoacidosis	0.0 (0)	26 (0.07)	21 (0.1)	8 (0.09)	<5	<5	<5

C10 severity score categories (0 - ≥6) are based on the simple count of severity domains using 10-year look-back window. Cell counts of less than five individuals can not be disclosed and are presented as <5 throughout the table. [&]most recent HbA_{1c} measure within 1year before or 6months after index date. **counts are merged with the Q5 band as they were less than 5 individuals.

ACS: acute coronary syndrome; **AF**: atrial fibrillation; **CHD**: coronary heart disease; **CHF**: congestive heart failure; **ESRD**: end stage renal disease; **HbA_{1c}**: glycated haemoglobin. **IMD**: index of multiple deprivation; **MI**: myocardial infarction; **PVD**: peripheral vascular disease; **SD**: standard deviation; **SVT**: supraventricular tachycardia; **TIA**: transient ischemic attack.

Supplementary data to "Development and validation of the DIabetes Severity SCOre (DISSCO)". Zghebi et al.

Table S7 Descriptive statistics of the measured severity scores at baseline (index date) and 1-5 years after diabetes diagnosis [Mean±SD (Range)] - Training dataset

	**Simple count (C) score**						**Severity-weighted (SW) score**						**Proximity-weighted score**
	Index date	Index+1 year	Index+2 years	Index+3 years	Index+4 years	Index+5 years	Index date	Index+1 year	Index+2 years	Index+3 years	Index+4 years	Index+5 years	Index date
**CU/ SWU**	1.5±1.6 (0-15)	1.7±1.6 (0-17)	1.8±1.7 (0-17)	2.0±1.7 (0-17)	2.1±1.8 (0-14)	2.2±1.8 (0-14)	1.6±2.3 (0-27.5)	1.8±2.4 (0-28)	2.0±2.6 (0-28)	2.1±2.6 (0-28)	2.3±2.7 (0-26)	2.4±2.8 (0-28)	NA
**C10/ SW10**	1.3±1.4 (0-12)	1.4±1.4 (0-14)	1.6±1.5 (0-14)	1.7±1.5 (0-13)	1.8±1.5 (0-13)	1.9±1.6 (0-13)	1.3±2.0 (0-22)	1.5±2.1 (0-24)	1.7±2.2 (0-24)	1.8±2.3 (0-26)	1.9±2.3 (0-22)	2.0±2.4 (0-26.5)	NA
**C5/ SW5/ PW**	1.0±1.2 (0-11)	1.1±1.2 (0-13)	1.3±1.3 (0-13)	1.3±1.3 (0-12)	1.4±1.3 (0-11)	1.4±1.3 (0-12)	1.1±1.7 (0-21.5)	1.2±1.8 (0-23.5)	1.3±1.8 (0-23.5)	1.4±1.9 (0-21.5)	1.5±1.9 (0-19.5)	1.5±2.0 (0-22.5)	0.60±0.77 (0-7.6)

Simple count (C) score measured using unlimited (CU), 10-year (C10), and 5-year (C5) look-back windows. Severity-weighted (SW) score measured using unlimited (SWU), 10-year (SW10), and 5-year (SW5) windows

Zghebi SS, *et al. BMJ Open Diab Res Care* 2020; 8:e000962. doi: 10.1136/bmjdrc-2019-000962

Supplementary data to "Development and validation of the DIabetes Severity SCOre (DISSCO)". Zghebi et al.

Table S8 Hazard ratios (95% CI) for risk of all-cause mortality associated with simple count and severity-weighted scores estimated at three baseline windows

Model	Predictors	Simple count (C) score		Severity-weighted (SW) score	
		HR (95% CI)	AUROC	HR (95% CI)	AUROC
Model 1	Age<br>Gender (F)<br>Deprivation (Q5 vs. Q1)<br>Ethnicity (vs. White)<br>• Indian<br>• Unknown	1.10 (1.10 to 1.10)<br>0.78 (0.76 to 0.80)<br>1.52 (1.45 to 1.58)<br><br>0.70 (0.61 to 0.80)<br>0.92 (0.87 to 0.98)	0.7528	-	
Model 2 (unlimited)	Severity score (CU / SWU)<br>Age<br>Gender (F)<br>Deprivation (Q5 vs. Q1)<br>Ethnicity (vs. White)<br>• Indian<br>• Unknown	1.14 (1.13 to 1.15)<br>1.09 (1.09 to 1.09)<br>0.81 (0.79 to 0.83)<br>1.47 (1.40 to 1.53)<br><br>0.70 (0.60 to 0.80)<br>0.99 (0.93 to 1.06)	0.7570	1.10 (1.10 to 1.11)<br>1.09 (1.09 to 1.09)<br>0.83 (0.81 to 0.85)<br>1.46 (1.39 to 1.52)<br><br>0.70 (0.60 to 0.80)<br>1.01 (0.95 to 1.07)	0.7556
Model 2 (10-year)	Severity score(C10/SW10)<br>Age<br>Gender (F)<br>Deprivation (Q5 vs. Q1)<br>Ethnicity (vs. White)<br>• Indian<br>• Unknown	1.14 (1.13 to 1.15)<br>1.09 (1.09 to 1.09)<br>0.80 (0.79 to 0.82)<br>1.47 (1.41 to 1.53)<br><br>0.70 (0.60 to 0.80)<br>0.98 (0.92 to 1.04)	0.7563	1.11 (1.10 to 1.11)<br>1.09 (1.09 to 1.09)<br>0.82 (0.79 to 0.84)<br>1.46 (1.40 to 1.52)<br><br>0.70 (0.60 to 0.80)<br>1.00 (0.94 to 1.06)	0.7553
Model 2 (5-year)	Severity score(C10/SW10)<br>Age<br>Gender (F)<br>Deprivation (Q5 vs. Q1)<br>Ethnicity (vs. White)<br>• Indian<br>• Unknown	1.13 (1.12 to 1.14)<br>1.09 (1.09 to 1.10)<br>0.79 (0.77 to 0.81)<br>1.48 (1.42 to 1.54)<br><br>0.70 (0.60 to 0.80)<br>0.96 (0.91 to 1.03)	0.7553	1.11 (1.11 to 1.12)<br>1.09 (1.09 to 1.09)<br>0.81 (0.78 to 0.83)<br>1.47 (1.41 to 1.54)<br><br>0.70 (0.61 to 0.80)<br>0.98 (0.92 to 1.04)	0.7548
Model 3	Age<br>Gender (F)<br>Deprivation (Q5 vs. Q1)<br>Ethnicity (vs. White)<br>• Indian<br>• Unknown<br>Baseline HbA_{1c}	1.10 (1.10 to 1.10)<br>0.77 (0.75 to 0.79)<br>1.52 (1.45 to 1.60)<br><br>0.70 (0.60 to 0.82)<br>0.89 (0.83 to 0.95)<br>1.04 (1.03 to 1.04)	0.7529	-	
Model 4 (unlimited)	Severity score (CU / SWU)<br>Age<br>Gender (F)<br>Deprivation (Q5 vs. Q1)<br>Ethnicity (vs. White)<br>• Indian<br>• Unknown<br>Baseline HbA_{1c}	1.15 (1.14 to 1.16)<br>1.09 (1.09 to 1.09)<br>0.81 (0.78 to 0.83)<br>1.46 (1.39 to 1.53)<br><br>0.70 (0.59 to 0.81)<br>0.96 (0.90 to 1.03)<br>1.05 (1.04 to 1.06)	0.7570	1.10 (1.10 to 1.11)<br>1.09 (1.09 to 1.09)<br>0.83 (0.80 to 0.85)<br>1.45 (1.38 to 1.52)<br><br>0.70 (0.59 to 0.81)<br>0.97 (0.91 to 1.04)<br>1.05 (1.04 to 1.05)	0.7554
Model 4 (10-year)	Severity score(C10/SW10)<br>Age<br>Gender (F)<br>Deprivation (Q5 vs. Q1)<br>Ethnicity (vs. White)<br>• Indian<br>• Unknown<br>Baseline HbA_{1c}	1.14 (1.13 to 1.15)<br>1.09 (1.09 to 1.10)<br>0.79 (0.77 to 0.82)<br>1.46 (1.39 to 1.53)<br><br>0.70 (0.59 to 0.81)<br>0.95 (0.88 to 1.01)<br>1.05 (1.04 to 1.05)	0.7563	1.11 (1.10 to 1.11)<br>1.09 (1.09 to 1.09)<br>0.81 (0.79 to 0.84)<br>1.45 (1.38 to 1.52)<br><br>0.70 (0.59 to 0.82)<br>0.96 (0.90 to 1.03)<br>1.04 (1.04 to 1.05)	0.7552
Model 4 (5-year)	Severity score(C5/SW5)<br>Age<br>Gender (F)<br>Deprivation (Q5 vs. Q1)<br>Ethnicity (vs. White)<br>• Indian<br>• Unknown<br>Baseline HbA_{1c}	1.13 (1.12 to 1.15)<br>1.10 (1.09 to 1.10)<br>0.79 (0.76 to 0.81)<br>1.47 (1.40 to 1.55)<br><br>0.70 (0.60 to 0.82)<br>0.93 (0.87 to 1.00)<br>1.04 (1.04 to 1.05)	0.7553	1.11 (1.11 to 1.12)<br>1.09 (1.09 to 1.10)<br>0.80 (0.78 to 0.82)<br>1.47 (1.40 to 1.54)<br><br>0.70 (0.60 to 0.82)<br>0.95 (0.89 to 1.02)<br>1.04 (1.04 to 1.05)	0.7547

AUROC: area under the Receiver Operating Characteristics curve; **HbA_{1c}**: glycated haemoglobin. Simple count (C) or severity-weighted (SW) score measured using unlimited (CU/SWU), 10-year (C10/ SW10), and 5-year (C5/SW5) look-back windows, respectively.

Supplementary data to "Development and validation of the DIabetes Severity SCOre (DISSCO)". Zghebi et al.

Table S9 Adjusted hazard ratios (95% CI) for all-cause mortality associated with simple count estimated at baseline and 1-5 years moving post-index windows

	Predictor(s)	Index date	Index+1 year	Index+2 years	Index+3 years	Index+4 years	Index+5 years
Model 1	Age*	1.10 (1.10 to 1.10)	1.10 (1.10 to 1.10)	1.10 (1.10 to 1.10)	1.10 (1.10 to 1.10)	1.10 (1.10 to 1.10)	1.10 (1.10 to 1.10)
	Gender (F)	0.78 (0.76 to 0.80)	0.78 (0.76 to 0.80)	0.78 (0.76 to 0.80)	0.77 (0.75 to 0.80)	0.77 (0.75 to 0.80)	0.77 (0.75 to 0.80)
	Deprivation (Q5 vs. Q1)	1.52 (1.45 to 1.58)	1.52 (1.45 to 1.58)	1.52 (1.45 to 1.59)	1.52 (1.45 to 1.59)	1.53 (1.46 to 1.61)	1.53 (1.46 to 1.61)
	Ethnicity (Indian vs. White)	0.70 (0.61 to 0.80)	0.70 (0.61 to 0.80)	0.71 (0.61 to 0.81)	0.69 (0.60 to 0.80)	0.70 (0.60 to 0.82)	0.63 (0.53 to 0.75)
	AUROC	**0.7528**	**0.7528**	**0.7523**	**0.7518**	**0.7500**	**0.7484**
Model 2 (unlimited)	CU score	1.14 (1.14 to 1.15)	1.14 (1.14 to 1.15)	1.15 (1.14 to 1.16)	1.15 (1.15 to 1.16)	1.16 (1.15 to 1.17)	1.16 (1.15 to 1.17)
	Age*	1.09 (1.09 to 1.09)	1.09 (1.09 to 1.09)	1.09 (1.09 to 1.09)	1.09 (1.09 to 1.09)	1.09 (1.09 to 1.09)	1.09 (1.09 to 1.09)
	Gender (F)	0.81 (0.79 to 0.83)	0.81 (0.79 to 0.83)	0.81 (0.79 to 0.84)	0.81 (0.79 to 0.84)	0.82 (0.79 to 0.84)	0.82 (0.80 to 0.85)
	Deprivation (Q5 vs. Q1)	1.47 (1.40 to 1.53)	1.46 (1.40 to 1.53)	1.45 (1.39 to 1.52)	1.45 (1.38 to 1.52)	1.46 (1.39 to 1.53)	1.46 (1.38 to 1.53)
	Ethnicity (Indian vs. White)	0.70 (0.60 to 0.80)	0.70 (0.61 to 0.80)	0.71 (0.61 to 0.81)	0.69 (0.59 to 0.80)	0.71 (0.60 to 0.82)	0.64 (0.54 to 0.75)
	AUROC	**0.7570**	**0.7573**	**0.7572**	**0.7572**	**0.7557**	**0.7546**
Model 2 (10-year)	C10 score	1.14 (1.13 to 1.15)	1.14 (1.13 to 1.15)	1.15 (1.14 to 1.16)	1.15 (1.14 to 1.16)	1.16 (1.15 to 1.17)	1.16 (1.15 to 1.17)
	Age*	1.09 (1.09 to 1.09)	1.09 (1.09 to 1.09)	1.09 (1.09 to 1.09)	1.09 (1.09 to 1.10)	1.09 (1.09 to 1.10)	1.09 (1.09 to 1.10)
	Gender (F)	0.80 (0.79 to 0.82)	0.80 (0.78 to 0.82)	0.80 (0.78 to 0.83)	0.80 (0.78 to 0.82)	0.80 (0.78 to 0.83)	0.80 (0.78 to 0.83)
	Deprivation (Q5 vs. Q1)	1.47 (1.41 to 1.53)	1.47 (1.40 to 1.53)	1.46 (1.40 to 1.53)	1.45 (1.39 to 1.52)	1.46 (1.40 to 1.54)	1.46 (1.39 to 1.54)
	Ethnicity (Indian vs. White)	0.70 (0.61 to 0.80)	0.70 (0.61 to 0.80)	0.71 (0.61 to 0.81)	0.69 (0.59 to 0.80)	0.70 (0.60 to 0.82)	0.64 (0.54 to 0.75)
	AUROC	**0.7563**	**0.7565**	**0.7563**	**0.7561**	**0.7546**	**0.7532**
Model 2 (5-year)	C5 score	1.13 (1.12 to 1.14)	1.14 (1.13 to 1.15)	1.14 (1.13 to 1.15)	1.14 (1.13 to 1.15)	1.15 (1.14 to 1.16)	1.15 (1.14 to 1.17)
	Age*	1.09 (1.09 to 1.10)	1.09 (1.09 to 1.09)	1.10 (1.09 to 1.10)	1.10 (1.09 to 1.10)	1.10 (1.09 to 1.10)	1.09 (1.09 to 1.10)
	Gender (F)	0.79 (0.77 to 0.81)	0.80 (0.78 to 0.82)	0.79 (0.77 to 0.81)	0.79 (0.77 to 0.81)	0.79 (0.77 to 0.82)	0.79 (0.77 to 0.82)
	Deprivation (Q5 vs. Q1)	1.48 (1.42 to 1.54)	1.47 (1.40 to 1.53)	1.47 (1.41 to 1.54)	1.47 (1.40 to 1.54)	1.48 (1.41 to 1.56)	1.48 (1.41 to 1.56)
	Ethnicity (Indian vs. White)	0.70 (0.60 to 0.80)	0.70 (0.61 to 0.80)	0.69 (0.60 to 0.81)	0.69 (0.60 to 0.81)	0.71 (0.61 to 0.83)	0.64 (0.54 to 0.76)
	AUROC	**0.7529**	**0.7555**	**0.7546**	**0.7547**	**0.7530**	**0.7516**
Model 3	Age*	1.10 (1.10 to 1.10)	1.10 (1.10 to 1.10)	1.10 (1.10 to 1.10)	1.10 (1.10 to 1.10)	1.10 (1.10 to 1.10)	1.10 (1.10 to 1.10)
	Gender (F)	0.77 (0.75 to 0.79)	0.77 (0.75 to 0.79)	0.77 (0.75 to 0.79)	0.77 (0.74 to 0.79)	0.77 (0.74 to 0.79)	0.77 (0.74 to 0.79)

Zghebi SS, *et al. BMJ Open Diab Res Care* 2020; 8:e000962. doi: 10.1136/bmjdrc-2019-000962

Supplementary data to "Development and validation of the DIabetes Severity SCOre (DISSCO)". Zghebi et al.

	Predictor(s)	Index date	Index+1 year	Index+2 years	Index+3 years	Index+4 years	Index+5 years
	Deprivation (Q5 vs. Q1)	1.52 (1.45 to 1.60)	1.52 (1.45 to 1.60)	1.52 (1.45 to 1.60)	1.52 (1.44 to 1.60)	1.54 (1.46 to 1.63)	1.54 (1.46 to 1.63)
	Ethnicity (Indian vs. White)	0.70 (0.60 to 0.82)	0.70 (0.60 to 0.82)	0.71 (0.60 to 0.83)	0.70 (0.59 to 0.82)	0.72 (0.60 to 0.85)	0.64 (0.53 to 0.77)
	HbA_{1c}[&]	1.04 (1.03 to 1.04)	1.04 (1.03 to 1.04)	1.03 (1.03 to 1.04)	1.03 (1.02 to 1.04)	1.03 (1.02 to 1.04)	1.03 (1.02 to 1.04)
	AUROC	**0.7570**	**0.7529**	**0.7524**	**0.7520**	**0.7505**	**0.7493**
Model 4 (unlimited)	CU score	1.15 (1.14 to 1.16)	1.15 (1.14 to 1.16)	1.15 (1.14 to 1.16)	1.15 (1.14 to 1.16)	1.16 (1.15 to 1.17)	1.16 (1.15 to 1.17)
	Age[*]	1.09 (1.09 to 1.09)	1.09 (1.09 to 1.09)	1.09 (1.09 to 1.09)	1.09 (1.09 to 1.09)	1.09 (1.09 to 1.09)	1.09 (1.09 to 1.09)
	Gender (F)	0.81 (0.78 to 0.83)	0.81 (0.78 to 0.83)	0.81 (0.78 to 0.83)	0.81 (0.78 to 0.83)	0.81 (0.79 to 0.84)	0.82 (0.79 to 0.85)
	Deprivation (Q5 vs. Q1)	1.46 (1.39 to 1.53)	1.45 (1.39 to 1.53)	1.45 (1.38 to 1.52)	1.44 (1.37 to 1.52)	1.46 (1.38 to 1.54)	1.45 (1.37 to 1.54)
	Ethnicity (Indian vs. White)	0.70 (0.59 to 0.81)	0.70 (0.60 to 0.82)	0.70 (0.60 to 0.83)	0.69 (0.59 to 0.82)	0.72 (0.61 to 0.86)	0.64 (0.53 to 0.78)
	HbA_{1c}[&]	1.05 (1.04 to 1.05)	1.05 (1.04 to 1.05)	1.04 (1.04 to 1.05)	1.04 (1.03 to 1.05)	1.04 (1.03 to 1.05)	1.04 (1.03 to 1.05)
	AUROC	**0.7563**	**0.7572**	**0.7571**	**0.7570**	**0.7559**	**0.7550**
Model 4 (10-year)	C10 score	1.14 (1.13 to 1.15)	1.14 (1.13 to 1.15)	1.15 (1.14 to 1.16)	1.15 (1.14 to 1.16)	1.16 (1.15 to 1.17)	1.16 (1.15 to 1.17)
	Age[*]	1.09 (1.09 to 1.10)	1.09 (1.09 to 1.10)	1.09 (1.09 to 1.10)	1.09 (1.09 to 1.10)	1.09 (1.09 to 1.10)	1.09 (1.09 to 1.10)
	Gender (F)	0.79 (0.77 to 0.82)	0.79 (0.77 to 0.82)	0.80 (0.77 to 0.82)	0.79 (0.77 to 0.82)	0.80 (0.77 to 0.82)	0.80 (0.77 to 0.83)
	Deprivation (Q5 vs. Q1)	1.46 (1.39 to 1.53)	1.46 (1.39 to 1.53)	1.46 (1.39 to 1.53)	1.45 (1.38 to 1.53)	1.47 (1.39 to 1.55)	1.47 (1.39 to 1.55)
	Ethnicity (Indian vs. White)	0.70 (0.59 to 0.81)	0.70 (0.60 to 0.82)	0.71 (0.60 to 0.83)	0.69 (0.59 to 0.82)	0.72 (0.60 to 0.86)	0.65 (0.53 to 0.78)
	HbA_{1c}[&]	1.05 (1.04 to 1.05)	1.05 (1.04 to 1.05)	1.04 (1.04 to 1.05)	1.04 (1.03 to 1.05)	1.04 (1.03 to 1.05)	1.04 (1.03 to 1.05)
	AUROC	**0.7563**	**0.7565**	**0.7562**	**0.7561**	**0.7548**	**0.7538**
Model 4 (5-year)	C5 score	1.13 (1.12 to 1.15)	1.14 (1.13 to 1.15)	1.14 (1.13 to 1.15)	1.14 (1.13 to 1.15)	1.15 (1.14 to 1.16)	1.16 (1.14 to 1.17)
	Age[*]	1.10 (1.09 to 1.10)	1.10 (1.09 to 1.10)	1.10 (1.09 to 1.10)	1.10 (1.09 to 1.10)	1.10 (1.09 to 1.10)	1.10 (1.09 to 1.10)
	Gender (F)	0.79 (0.76 to 0.81)	0.79 (0.76 to 0.81)	0.78 (0.76 to 0.81)	0.78 (0.76 to 0.81)	0.79 (0.76 to 0.81)	0.79 (0.76 to 0.82)
	Deprivation (Q5 vs. Q1)	1.47 (1.40 to 1.55)	1.47 (1.40 to 1.55)	1.47 (1.40 to 1.55)	1.47 (1.40 to 1.55)	1.49 (1.41 to 1.57)	1.49 (1.41 to 1.58)
	Ethnicity (Indian vs. White)	0.70 (0.60 to 0.82)	0.70 (0.60 to 0.82)	0.70 (0.59 to 0.83)	0.70 (0.59 to 0.83)	0.73 (0.62 to 0.87)	0.65 (0.54 to 0.79)
	HbA_{1c}[&]	1.04 (1.04 to 1.05)	1.04 (1.04 to 1.05)	1.04 (1.03 to 1.05)	1.04 (1.03 to 1.04)	1.04 (1.03 to 1.04)	1.03 (1.03 to 1.04)
	AUROC	**0.7553**	**0.7555**	**0.7546**	**0.7547**	**0.7534**	**0.7523**

Simple count (C) score measured using unlimited (CU), 10-year (C10), and 5-year (C5) look-back windows. *Age at index date. [&]baseline, most recent measure within 1year before or 6months after. **AUROC**: area under the Receiver Operating Characteristics curve**;** **HbA_{1c}**: glycated haemoglobin.

Zghebi SS, *et al. BMJ Open Diab Res Care* 2020; 8:e000962. doi: 10.1136/bmjdrc-2019-000962

Supplementary data to "Development and validation of the DIabetes Severity SCOre (DISSCO)". Zghebi et al.

Table S10 Adjusted hazard ratios (95% CI) for all-cause mortality associated with severity-weighted score estimated at baseline and 1-5 years moving post-index windows

	Predictor(s)	Index date	Index+1 year	Index+2 years	Index+3 years	Index+4 years	Index+5 years
Model 1	Age*	1.10 (1.10 to 1.10)	1.10 (1.10 to 1.10)	1.10 (1.10 to 1.10)	1.10 (1.10 to 1.10)	1.10 (1.10 to 1.10)	1.10 (1.10 to 1.10)
	Gender (F)	0.78 (0.76 to 0.80)	0.78 (0.76 to 0.80)	0.78 (0.76 to 0.80)	0.77 (0.75 to 0.80)	0.77 (0.75 to 0.80)	0.77 (0.75 to 0.80)
	Deprivation (Q5 vs. Q1)	1.52 (1.45 to 1.58)	1.52 (1.45 to 1.58)	1.52 (1.45 to 1.59)	1.52 (1.45 to 1.59)	1.53 (1.46 to 1.61)	1.53 (1.46 to 1.61)
	Ethnicity (Indian vs. White)	0.70 (0.61 to 0.80)	0.70 (0.61 to 0.80)	0.71 (0.61 to 0.81)	0.69 (0.60 to 0.80)	0.70 (0.60 to 0.82)	0.63 (0.53 to 0.75)
	AUROC	**0.7528**	**0.7528**	**0.7523**	**0.7518**	**0.7500**	**0.7484**
Model 2 (unlimited)	CU score	1.10 (1.10 to 1.11)	1.10 (1.10 to 1.11)	1.11 (1.10 to 1.11)	1.11 (1.10 to 1.11)	1.12 (1.11 to 1.13)	1.11 (1.11 to 1.13)
	Age*	1.09 (1.09 to 1.09)	1.09 (1.09 to 1.09)	1.09 (1.09 to 1.09)	1.09 (1.09 to 1.09)	1.09 (1.09 to 1.10)	1.09 (1.09 to 1.09)
	Gender (F)	0.83 (0.81 to 0.85)	0.83 (0.81 to 0.86)	0.84 (0.81 to 0.86)	0.84 (0.81 to 0.86)	0.80 (0.79 to 0.83)	0.85 (0.82 to 0.87)
	Deprivation (Q5 vs. Q1)	1.46 (1.39 to 1.52)	1.45 (1.39 to 1.52)	1.45 (1.38 to 1.51)	1.44 (1.37 to 1.51)	1.46 (1.39 to 1.54)	1.45 (1.38 to 1.52)
	Ethnicity (Indian vs. White)	0.70 (0.60 to 0.80)	0.70 (0.61 to 0.80)	0.70 (0.61 to 0.80)	0.69 (0.59 to 0.80)	0.70 (0.59 to 0.83)	0.64 (0.54 to 0.75)
	AUROC	**0.7556**	**0.7559**	**0.7558**	**0.7556**	**0.7542**	**0.7531**
Model 2 (10-year)	C10 score	1.11 (1.10 to 1.11)	1.11 (1.10 to 1.12)	1.11 (1.11 to 1.12)	1.11 (1.11 to 1.12)	1.12 (1.11 to 1.12)	1.12 (1.11 to 1.12)
	Age*	1.09 (1.09 to 1.09)	1.09 (1.09 to 1.09)	1.09 (1.09 to 1.09)	1.09 (1.09 to 1.09)	1.09 (1.09 to 1.09)	1.09 (1.09 to 1.09)
	Gender (F)	0.82 (0.79 to 0.84)	0.82 (0.80 to 0.84)	0.82 (0.80 to 0.85)	0.82 (0.80 to 0.84)	0.82 (0.80 to 0.85)	0.83 (0.80 to 0.85)
	Deprivation (Q5 vs. Q1)	1.46 (1.40 to 1.52)	1.46 (1.40 to 1.52)	1.45 (1.39 to 1.52)	1.44 (1.38 to 1.51)	1.46 (1.39 to 1.53)	1.46 (1.39 to 1.54)
	Ethnicity (Indian vs. White)	0.70 (0.60 to 0.80)	0.70 (0.61 to 0.80)	0.70 (0.61 to 0.81)	0.69 (0.59 to 0.80)	0.71 (0.61 to 0.82)	0.64 (0.54 to 0.75)
	AUROC	**0.7553**	**0.7556**	**0.7554**	**0.7552**	**0.7536**	**0.7525**
Model 2 (5-year)	C5 score	1.11 (1.11 to 1.12)	1.11 (1.11 to 1.12)	1.12 (1.11 to 1.12)	1.12 (1.11 to 1.13)	1.12 (1.12 to 1.13)	1.13 (1.12 to 1.13)
	Age*	1.09 (1.09 to 1.09)	1.09 (1.09 to 1.09)	1.09 (1.09 to 1.10)	1.09 (1.09 to 1.10)	1.09 (1.09 to 1.10)	1.09 (1.09 to 1.10)
	Gender (F)	0.81 (0.78 to 0.83)	0.81 (0.79 to 0.83)	0.81 (0.78 to 0.83)	0.81 (0.79 to 0.83)	0.81 (0.79 to 0.84)	0.81 (0.79 to 0.84)
	Deprivation (Q5 vs. Q1)	1.47 (1.41 to 1.54)	1.47 (1.41 to 1.53)	1.46 (1.40 to 1.53)	1.46 (1.39 to 1.53)	1.47 (1.40 to 1.55)	1.47 (1.40 to 1.55)
	Ethnicity (Indian vs. White)	0.70 (0.61 to 0.80)	0.70 (0.61 to 0.80)	0.69 (0.60 to 0.80)	0.69 (0.60 to 0.80)	0.71 (0.61 to 0.83)	0.64 (0.54 to 0.76)
	AUROC	**0.7548**	**0.7551**	**0.7542**	**0.7545**	**0.7527**	**0.7513**
Model 3	Age*	1.10 (1.10 to 1.10)	1.10 (1.10 to 1.10)	1.10 (1.10 to 1.10)	1.10 (1.10 to 1.10)	1.10 (1.10 to 1.10)	1.10 (1.10 to 1.10)
	Gender (F)	0.77 (0.75 to 0.79)	0.77 (0.75 to 0.79)	0.77 (0.75 to 0.79)	0.77 (0.74 to 0.79)	0.77 (0.74 to 0.79)	0.77 (0.74 to 0.79)

Supplementary data to "Development and validation of the DIabetes Severity SCOre (DISSCO)". Zghebi et al.

	Predictor(s)	Index date	Index+1 year	Index+2 years	Index+3 years	Index+4 years	Index+5 years
	Deprivation (Q5 vs. Q1)	1.52 (1.45 to 1.60)	1.52 (1.45 to 1.60)	1.52 (1.45 to 1.60)	1.52 (1.44 to 1.60)	1.54 (1.46 to 1.63)	1.54 (1.46 to 1.63)
	Ethnicity (Indian vs. White)	0.70 (0.60 to 0.82)	0.70 (0.60 to 0.82)	0.71 (0.60 to 0.83)	0.70 (0.59 to 0.82)	0.72 (0.60 to 0.85)	0.64 (0.53 to 0.77)
	HbA_{1c}[&]	1.04 (1.03 to 1.04)	1.04 (1.03 to 1.04)	1.03 (1.03 to 1.04)	1.03 (1.02 to 1.04)	1.03 (1.02 to 1.04)	1.03 (1.02 to 1.04)
	AUROC	**0.7529**	**0.7529**	**0.7524**	**0.7520**	**0.7505**	**0.7493**
Model 4 (unlimited)	CU score	1.10 (1.10 to 1.11)	1.11 (1.10 to 1.11)	1.11 (1.10 to 1.11)	1.11 (1.10 to 1.11)	1.11 (1.10 to 1.11)	1.11 (1.10 to 1.11)
	Age*	1.09 (1.09 to 1.09)	1.09 (1.09 to 1.09)	1.09 (1.09 to 1.09)	1.09 (1.09 to 1.09)	1.09 (1.09 to 1.09)	1.09 (1.09 to 1.09)
	Gender (F)	0.83 (0.80 to 0.85)	0.83 (0.80 to 0.85)	0.83 (0.81 to 0.86)	0.83 (0.81 to 0.86)	0.84 (0.81 to 0.87)	0.84 (0.81 to 0.87)
	Deprivation (Q5 vs. Q1)	1.45 (1.38 to 1.52)	1.45 (1.38 to 1.52)	1.44 (1.37 to 1.51)	1.44 (1.36 to 1.51)	1.45 (1.37 to 1.53)	1.45 (1.36 to 1.53)
	Ethnicity (Indian vs. White)	0.70 (0.59 to 0.81)	0.70 (0.60 to 0.82)	0.70 (0.60 to 0.82)	0.69 (0.59 to 0.82)	0.72 (0.61 to 0.86)	0.64 (0.53 to 0.78)
	HbA_{1c}[&]	1.05 (1.04 to 1.05)	1.05 (1.04 to 1.05)	1.04 (1.04 to 1.05)	1.04 (1.03 to 1.04)	1.04 (1.03 to 1.05)	1.04 (1.03 to 1.05)
	AUROC	**0.7554**	**0.7557**	**0.7555**	**0.7554**	**0.7543**	**0.7535**
Model 4 (10-year)	C10 score	1.11 (1.10 to 1.11)	1.10 (1.10 to 1.12)	1.11 (1.11 to 1.12)	1.11 (1.11 to 1.12)	1.12 (1.11 to 1.12)	1.12 (1.11 to 1.12)
	Age*	1.09 (1.09 to 1.09)	1.09 (1.09 to 1.09)	1.09 (1.09 to 1.09)	1.09 (1.09 to 1.09)	1.09 (1.09 to 1.09)	1.09 (1.09 to 1.09)
	Gender (F)	0.81 (0.79 to 0.84)	0.81 (0.79 to 0.84)	0.82 (0.79 to 0.84)	0.82 (0.79 to 0.84)	0.82 (0.79 to 0.85)	0.82 (0.79 to 0.85)
	Deprivation (Q5 vs. Q1)	1.45 (1.38 to 1.52)	1.45 (1.38 to 1.52)	1.45 (1.38 to 1.52)	1.44 (1.37 to 1.52)	1.46 (1.38 to 1.54)	1.46 (1.38 to 1.55)
	Ethnicity (Indian vs. White)	0.70 (0.59 to 0.82)	0.70 (0.60 to 0.82)	0.70 (0.60 to 0.83)	0.69 (0.59 to 0.82)	0.73 (0.61 to 0.86)	0.65 (0.53 to 0.78)
	HbA_{1c}[&]	1.04 (1.04 to 1.05)	1.04 (1.04 to 1.05)	1.04 (1.03 to 1.05)	1.04 (1.03 to 1.04)	1.04 (1.03 to 1.04)	1.04 (1.03 to 1.04)
	AUROC	**0.7552**	**0.7555**	**0.7553**	**0.7551**	**0.7539**	**0.7530**
Model 4 (5-year)	C5 score	1.11 (1.11 to 1.12)	1.11 (1.11 to 1.12)	1.12 (1.11 to 1.13)	1.12 (1.11 to 1.13)	1.12 (1.12 to 1.13)	1.13 (1.12 to 1.13)
	Age*	1.09 (1.09 to 1.10)	1.09 (1.09 to 1.10)	1.09 (1.09 to 1.10)	1.09 (1.09 to 1.10)	1.09 (1.09 to 1.10)	1.09 (1.09 to 1.10)
	Gender (F)	0.80 (0.78 to 0.82)	0.80 (0.78 to 0.83)	0.80 (0.78 to 0.83)	0.80 (0.78 to 0.83)	0.81 (0.78 to 0.84)	0.81 (0.78 to 0.84)
	Deprivation (Q5 vs. Q1)	1.47 (1.40 to 1.54)	1.47 (1.40 to 1.54)	1.47 (1.39 to 1.54)	1.46 (1.39 to 1.54)	1.48 (1.40 to 1.56)	1.48 (1.40 to 1.57)
	Ethnicity (Indian vs. White)	0.70 (0.60 to 0.82)	0.70 (0.60 to 0.82)	0.70 (0.59 to 0.83)	0.70 (0.59 to 0.83)	0.74 (0.62 to 0.87)	0.65 (0.54 to 0.79)
	HbA_{1c}[&]	1.04 (1.04 to 1.05)	1.04 (1.04 to 1.05)	1.04 (1.03 to 1.04)	1.03 (1.03 to 1.04)	1.03 (1.03 to 1.04)	1.03 (1.02 to 1.04)
	AUROC	**0.7547**	**0.7550**	**0.7542**	**0.7545**	**0.7532**	**0.7520**

Severity-weighted (SW) score measured using unlimited (SWU), 10-year (SW10), and 5-year (SW5) windows. *Age at index date. [&]baseline, most recent measure within 1year before or 6months after. **AUROC**: area under the Receiver Operating Characteristics curve**;** **HbA_{1c}**: glycated haemoglobin.

Zghebi SS, *et al. BMJ Open Diab Res Care* 2020; 8:e000962. doi: 10.1136/bmjdrc-2019-000962

Supplementary data to "Development and validation of the DIabetes Severity SCOre (DISSCO)". Zghebi et al.

Table S11 Hazard ratios (95% CI) for risk of all-cause hospitalisation associated with simple count and severity-weighted scores estimated at three windows - Training data

Model	Predictor(s)	Simple count (C) score		Severity-weighted (SW) score	
		**HR (95% CI)**	**AUROC**	**HR (95% CI)**	**AUROC**
Model 1	**Age**	1.02 (1.02 to 1.02)	0.6231	-	
	**Gender (F)**	1.01 (0.99 to 1.02)			
	**Deprivation (Q5 vs. Q1)**	1.21 (1.18 to 1.24)			
	**Ethnicity** (vs. White)				
	• Indian	1.00 (0.95 to 1.05)			
	• Unknown	0.20 (0.19 to 0.21)			
Model 2 (unlimited)	**Severity score** (CU / SWU)	1.09 (1.09 to 1.10)	0.6298	1.07 (1.06 to 1.07)	0.6299
	**Age**	1.02 (1.02 to 1.02)		1.02 (1.02 to 1.02)	
	**Gender (F)**	1.03 (1.02 to 1.05)		1.04 (1.03 to 1.06)	
	**Deprivation (Q5 vs. Q1)**	1.19 (1.16 to 1.22)		1.19 (1.16 to 1.22)	
	**Ethnicity** (vs. White)				
	• Indian	1.00 (0.95 to 1.05)		1.00 (0.95 to 1.06)	
	• Unknown	0.20 (0.19 to 0.21)		0.20 (0.19 to 0.21)	
Model 2 (10-year)	**Severity score**(C10/SW10)	1.09 (1.09 to 1.10)	0.6288	1.07 (1.07 to 1.07)	0.6291
	**Age**	1.02 (1.02 to 1.02)		1.02 (1.02 to 1.02)	
	**Gender (F)**	1.03 (1.01 to 1.04)		1.04 (1.02 to 1.05)	
	**Deprivation (Q5 vs. Q1)**	1.19 (1.16 to 1.22)		1.19 (1.16 to 1.22)	
	**Ethnicity** (vs. White)				
	• Indian	1.00 (0.95 to 1.05)		1.00 (0.95 to 1.05)	
	• Unknown	0.20 (0.19 to 0.21)		0.20 (0.19 to 0.21)	
Model 2 (5-year)	**Severity score**(C10/SW10)	1.09 (1.09 to 1.10)	0.6274	1.07 (1.06 to 1.08)	0.6280
	**Age**	1.02 (1.02 to 1.02)		1.03 (1.01 to 1.04)	
	**Gender (F)**	1.02 (1.01 to 1.03)		1.04 (1.03 to 1.06)	
	**Deprivation** (Q5 vs. Q1)	1.20 (1.17 to 1.22)		1.19 (1.17 to 1.22)	
	**Ethnicity** (vs. White)				
	• Indian	1.00 (0.96 to 1.06)		1.01 (0.96 to 1.06)	
	• Unknown	0.20 (0.19 to 0.21)		0.20 (0.19 to 0.21)	
Model 3	**Age**	1.02 (1.02 to 1.02)	0.6230	-	
	**Gender (F)**	1.00 (0.98 to 1.01)			
	**Deprivation** (Q5 vs. Q1)	1.21 (1.18 to 1.24)			
	**Ethnicity** (vs. White)				
	• Indian	1.00 (0.93 to 1.04)			
	• Unknown	0.19 (0.18 to 0.20)			
	**Baseline HbA_{1c}**	0.99 (0.99 to 1.00)			
Model 4 (unlimited)	**Severity score** (CU / SWU)	1.09 (1.08 to 1.09)	0.6293	1.06 (1.06 to 1.07)	0.6293
	**Age**	1.02 (1.02 to 1.02)		1.02 (1.02 to 1.02)	
	**Gender (F)**	1.02 (1.01 to 1.04)		1.03 (1.02 to 1.05)	
	**Deprivation** (Q5 vs. Q1)	1.19 (1.16 to 1.21)		1.18 (1.15 to 1.21)	
	**Ethnicity** (vs. White)				
	• Indian	0.99 (0.93 to 1.04)		0.99 (0.93 to 1.04)	
	• Unknown	0.19 (0.18 to 0.20)		0.19 (0.19 to 0.20)	
	**Baseline HbA_{1c}**	1.00 (1.00 to 1.00)		1.00 (0.99 to 1.00)	
Model 4 (10-year)	**Severity score**(C10/SW10)	1.09 (1.08 to 1.09)	0.6283	1.07 (1.06 to 1.07)	0.6286
	**Age**	1.02 (1.02 to 1.02)		1.02 (1.02 to 1.02)	
	**Gender (F)**	1.02 (1.00 to 1.03)		1.03 (1.01 to 1.04)	
	**Deprivation** (Q5 vs. Q1)	1.19 (1.16 to 1.21)		1.18 (1.16 to 1.21)	
	**Ethnicity** (vs. White)				
	• Indian	0.98 (0.93 to 1.04)		0.99 (0.93 to 1.04)	
	• Unknown	0.19 (0.18 to 0.20)		0.19 (0.19 to 0.20)	
	**Baseline HbA_{1c}**	1.00 (1.00 to 1.00)		1.00 (0.99 to 1.00)	
Model 4 (5-year)	**Severity score**(C5/SW5)	1.09 (1.08 to 1.10)	0.6270	1.07 (1.07 to 1.08)	0.6276
	**Age**	1.02 (1.02 to 1.02)		1.02 (1.02 to 1.02)	
	**Gender (F)**	1.00 (1.00 to 1.02)		1.02 (1.00 to 1.03)	
	**Deprivation** (Q5 vs. Q1)	1.19 (1.16 to 1.22)		1.19 (1.16 to 1.22)	
	**Ethnicity** (vs. White)				
	• Indian	0.99 (0.93 to 1.04)		0.99 (0.93 to 1.04)	
	• Unknown	0.19 (0.18 to 0.20)		0.19 (0.18 to 0.20)	
	**Baseline HbA_{1c}**	1.00 (0.99 to 1.00)		1.00 (0.99 to 1.00)	

AUROC: area under the Receiver Operating Characteristics curve; **HbA_{1c}**: glycated haemoglobin. Simple count (C) or severity-weighted (SW) score measured using unlimited (CU/SWU), 10-year (C10/ SW10), and 5-year (C5/SW5) look-back windows, respectively.

Supplementary data to "Development and validation of the DIabetes Severity SCOre (DISSCO)". Zghebi et al.

Table S12 Hazard ratios (95% CI) for risk of CV-related hospitalisation associated with simple count and severity-weighted scores estimated at three windows

Model	Predictors	Simple count (C) score		Severity-weighted (SW) score	
		HR (95% CI)	AUROC	HR (95% CI)	AUROC
Model 1	**Age**	1.06 (1.06 to 1.06)	0.7033	-	
	**Gender (F)**	0.71 (0.69 to 0.72)			
	**Deprivation (Q5 vs. Q1)**	1.42 (1.38 to 1.47)			
	**Ethnicity** (vs. White)				
	• Indian	0.96 (0.88 to 1.04)			
	• Unknown	0.19 (0.17 to 0.20)			
Model 2 (unlimited)	**Severity score** (CU / SWU)	1.42 (1.41 to 1.43)	0.7281	1.22 (1.22 to 1.22)	0.7169
	**Age**	1.04 (1.04 to 1.04)		1.04 (1.04 to 1.04)	
	**Gender (F)**	0.79 (0.76 to 0.81)		0.81 (0.79 to 0.82)	
	**Deprivation (Q5 vs. Q1)**	1.34 (1.29 to 1.38)		1.35 (1.30 to 1.39)	
	**Ethnicity** (vs. White)				
	• Indian	0.95 (0.88 to 1.03)		0.96 (0.88 to 1.04)	
	• Unknown	0.21 (0.20 to 0.23)		0.21 (0.20 to 0.23)	
Model 2 (10-year)	**Severity score**(C10/SW10)	1.44 (1.43 to 1.45)	0.7267	1.24 (1.23 to 1.24)	0.7162
	**Age**	1.04 (1.04 to 1.05)		1.05 (1.04 to 1.06)	
	**Gender (F)**	0.77 (0.75 to 0.79)		0.78 (0.77 to 0.80)	
	**Deprivation (Q5 vs. Q1)**	1.33 (1.29 to 1.37)		1.34 (1.30 to 1.39)	
	**Ethnicity** (vs. White)				
	• Indian	0.95 (0.87 to 1.03)		0.96 (0.88 to 1.04)	
	• Unknown	0.21 (0.20 to 0.23)		0.21 (0.20 to 0.23)	
Model 2 (5-year)	**Severity score**(C10/SW10)	1.45 (1.45 to 1.47)	0.7226	1.26 (1.25 to 1.26)	0.7138
	**Age**	1.05 (1.05 to 1.05)		1.05 (1.05 to 1.05)	
	**Gender (F)**	0.75 (0.73 to 0.77)		0.77 (0.75 to 0.78)	
	**Deprivation** (Q5 vs. Q1)	1.34 (1.30 to 1.39)		1.36 (1.31 to 1.40)	
	**Ethnicity** (vs. White)				
	• Indian	0.96 (0.88 to 1.04)		0.96 (0.89 to 1.05)	
	• Unknown	0.21 (0.19 to 0.22)		0.21 (0.19 to 0.23)	
Model 3	**Age**	1.06 (1.06 to 1.06)	0.7057	-	
	**Gender (F)**	0.70 (0.69 to 0.72)			
	**Deprivation** (Q5 vs. Q1)	1.42 (1.37 to 1.47)			
	**Ethnicity** (vs. White)				
	• Indian	0.93 (0.85 to 1.02)			
	• Unknown	0.18 (0.16 to 0.19)			
	**Baseline HbA_{1c}**	1.00 (0.99 to 1.00)			
Model 4 (unlimited)	**Severity score** (CU / SWU)	1.42 (1.42 to 1.43)	0.7298	1.22 (1.22 to 1.22)	0.7185
	**Age**	1.04 (1.04 to 1.04)		1.04 (1.04 to 1.05)	
	**Gender (F)**	0.79 (0.77 to 0.81)		0.80 (0.79 to 0.82)	
	**Deprivation** (Q5 vs. Q1)	1.32 (1.27 to 1.36)		1.32 (1.28 to 1.38)	
	**Ethnicity** (vs. White)				
	• Indian	0.91 (0.83 to 1.00)		0.92 (0.84 to 1.01)	
	• Unknown	0.20 (0.19 to 0.22)		0.20 (0.19 to 0.22)	
	**Baseline HbA_{1c}**	1.03 (1.02 to 1.03)		1.01 (1.01 to 1.01)	
Model 4 (10-year)	**Severity score**(C10/SW10)	1.45 (1.43 to 1.46)	0.7288	1.24 (1.23 to 1.24)	0.7179
	**Age**	1.05 (1.04 to 1.05)		1.05 (1.05 to 1.05)	
	**Gender (F)**	0.77 (0.75 to 0.79)		0.78 (0.76 to 0.80)	
	**Deprivation** (Q5 vs. Q1)	1.31 (1.26 to 1.35)		1.32 (1.28 to 1.37)	
	**Ethnicity** (vs. White)				
	• Indian	0.91 (0.83 to 1.00)		0.92 (0.84 to 1.01)	
	• Unknown	0.20 (0.18 to 0.22)		0.20 (0.18 to 0.22)	
	**Baseline HbA_{1c}**	1.02 (1.02 to 1.03)		1.01 (1.01 to 1.02)	
Model 4 (5-year)	**Severity score**(C5/SW5)	1.47 (1.46 to 1.48)	0.7247	1.26 (1.25 to 1.26)	0.7156
	**Age**	1.05 (1.05 to 1.05)		1.05 (1.05 to 1.05)	
	**Gender (F)**	0.75 (0.73 to 0.77)		0.76 (0.75 to 0.78)	
	**Deprivation** (Q5 vs. Q1)	1.32 (1.28 to 1.37)		1.34 (1.29 to 1.39)	
	**Ethnicity** (vs. White)				
	• Indian	0.92 (0.84 to 1.01)		0.93 (0.85 to 1.02)	
	• Unknown	0.20 (0.18 to 0.21)		0.20 (0.18 to 0.22)	
	**Baseline HbA_{1c}**	1.02 (1.02 to 1.03)		1.01 (1.01 to 1.02)	

AUROC: area under the Receiver Operating Characteristics curve; **HbA_{1c}**: glycated haemoglobin. Simple count (C) or severity-weighted (SW) score measured using unlimited (CU/SWU), 10-year (C10/ SW10), and 5-year (C5/SW5) look-back windows, respectively.

Zghebi SS, *et al. BMJ Open Diab Res Care* 2020; 8:e000962. doi: 10.1136/bmjdrc-2019-000962

Supplementary data to "Development and validation of the DIabetes Severity SCOre (DISSCO)". Zghebi et al.

Table S13 Hazard ratios for risk of diabetes-related hospitalisation associated with simple count and severity-weighted scores estimated at three baseline windows - Training dataset

Model	Predictor(s)	Simple count (C) score		Severity-weighted (SW) score	
		HR (95% CI)	AUROC	HR (95% CI)	AUROC
**Model 1**	**Age**	1.02 (1.02 to 1.02)	0.6249	-	
	**Gender (F)**	0.99 (0.97 to 1.00)			
	**Deprivation** (Q5 vs. Q1)	1.29 (1.26 to 1.32)			
	**Ethnicity (vs. White)**				
	• Indian	0.95 (0.90 to 1.01)			
	• Unknown	0.19 (0.18 to 0.20)			
**Model 2 (unlimited)**	**Severity score** (CU/SWU)	1.10 (1.09 to 1.10)	0.6324	1.07 (1.07 to 1.07)	0.6323
	**Age**	1.01 (1.02 to 1.02)		1.02 (1.02 to 1.02)	
	**Gender (F)**	1.01 (1.01 to 1.03)		1.02 (1.01 to 1.04)	
	**Deprivation** (Q5 vs. Q1)	1.27 (1.24 to 1.30)		1.26 (1.23 to 1.30)	
	**Ethnicity (vs. White)**				
	• Indian	0.95 (0.90 to 1.00)		0.95 (0.90 to 1.00)	
	• Unknown	0.20 (0.19 to 0.21)		0.20 (0.19 to 0.21)	
**Model 2 (10-year)**	**Severity score** (C10/SW10)	1.10 (1.09 to 1.11)	0.6312	1.07 (1.07 to 1.08)	0.6315
	**Age**	1.02 (1.02 to 1.02)		1.02 (1.02 to 1.02)	
	**Gender (F)**	1.00 (0.99 to 1.02)		1.01 (1.00 to 1.03)	
	**Deprivation** (Q5 vs. Q1)	1.27 (1.24 to 1.30)		1.27 (1.24 to 1.30)	
	**Ethnicity (vs. White)**				
	• Indian	0.95 (0.90 to 1.00)		0.95 (0.90 to 1.00)	
	• Unknown	0.20 (0.19 to 0.21)		0.20 (0.19 to 0.21)	
**Model 2 (5-year)**	**Severity score** (C5/SW5)	1.10 (1.09 to 1.11)	0.6297	1.08 (1.07 to 1.08)	0.6302
	**Age**	1.02 (1.02 to 1.02)		1.02 (1.02 to 1.02)	
	**Gender (F)**	1.00 (0.98 to 1.01)		1.01 (0.99 to 1.02)	
	**Deprivation** (Q5 vs. Q1)	1.27 (1.24 to 1.30)		1.27 (1.24 to 1.30)	
	**Ethnicity (vs. White)**				
	• Indian	0.95 (0.90 to 1.01)		0.95 (0.90 to 1.01)	
	• Unknown	0.19 (0.18 to 0.20)		0.20 (0.19 to 0.21)	
**Model 3**	**Age**	1.02 (1.02 to 1.02)	0.6254	-	
	**Gender (F)**	0.98 (0.97 to 1.00)			
	**Deprivation** (Q5 vs. Q1)	1.28 (1.25 to 1.32)			
	**Ethnicity (vs. White)**				
	• Indian	0.93 (0.88 to 0.99)			
	• Unknown	0.18 (0.17 to 0.19)			
	Baseline HbA_{1c}	1.01 (1.01 to 1.02)			
**Model 4 (unlimited)**	**Severity score** (CU/SWU)	1.10 (1.09 to 1.10)	0.6327	1.07 (1.06 to 1.07)	0.6325
	**Age**	1.02 (1.02 to 1.02)		1.02 (1.02 to 1.02)	
	**Gender (F)**	1.01 (0.99 to 1.03)		1.00 (1.00 to 1.04)	
	**Deprivation** (Q5 vs. Q1)	1.26 (1.23 to 1.29)		1.26 (1.22 to 1.29)	
	**Ethnicity (vs. White)**				
	• Indian	0.93 (0.87 to 0.98)		0.93 (0.87 to 0.98)	
	• Unknown	0.18 (0.17 to 0.20)		0.19 (0.18 to 0.20)	
	Baseline HbA_{1c}	1.02 (1.02 to 1.03)		1.02 (1.02 to 1.02)	
**Model 4 (10-year)**	**Severity score** (C10/SW10)	1.10 (1.09 to 1.10)	0.6316	1.07 (1.07 to 1.08)	0.6317
	**Age**	1.02 (1.02 to 1.02)		1.02 (1.02 to 1.02)	
	**Gender (F)**	1.00 (0.99 to 1.02)		1.01 (0.99 to 1.03)	
	**Deprivation** (Q5 vs. Q1)	1.26 (1.23 to 1.29)		1.26 (1.23 to 1.29)	
	**Ethnicity (vs. White)**				
	• Indian	0.92 (0.87 to 0.98)		0.92 (0.87 to 0.98)	
	• Unknown	0.20 (0.19 to 0.21)		0.19 (0.18 to 0.20)	
	Baseline HbA_{1c}	1.02 (1.02 to 1.02)		1.02 (1.02 to 1.02)	
**Model 4 (5-year)**	**Severity score** (C5/SW5)	1.10 (1.09 to 1.11)	0.6302	1.08 (1.07 to 1.08)	0.6306
	**Age**	1.02 (1.02 to 1.02)		1.02 (1.02 to 1.02)	
	**Gender (F)**	1.00 (0.98 to 1.01)		1.00 (0.99 to 1.02)	
	**Deprivation** (Q5 vs. Q1)	1.27 (1.23 to 1.30)		1.26 (1.23 to 1.30)	
	**Ethnicity (vs. White)**				
	• Indian	0.93 (0.87 to 0.99)		0.93 (0.87 to 0.98)	
	• Unknown	0.18 (0.17 to 0.19)		0.18 (0.17 to 0.19)	
	Baseline HbA_{1c}	1.02 (1.02 to 1.02)		1.02 (1.01 to 1.02)	

Zghebi SS, *et al. BMJ Open Diab Res Care* 2020; 8:e000962. doi: 10.1136/bmjdrc-2019-000962

Supplementary data to "Development and validation of the DIabetes Severity SCOre (DISSCO)". Zghebi et al.

Table S14 Hazard ratios (95% CI) for risk of hospitalisation due to hypoglycaemia associated with simple count and severity-weighted scores estimated at three baseline windows (secondary outcome) - Training dataset

Model	Predictor(s)	Simple count (C) score		Severity-weighted (SW) score	
		HR (95% CI)	AUROC	HR (95% CI)	AUROC
**Model 1**	**Age**	1.05 (1.05 to 1.06)	0.6997	-	
	**Gender (F)**	1.01 (0.94 to 1.08)			
	**Deprivation** (Q5 vs. Q1)	1.83 (1.64 to 2.06)			
	**Ethnicity (vs. White)**				
	• Indian	0.70 (0.52 to 0.95)			
	• Unknown	0.19 (0.14 to 0.26)			
**Model 2 (unlimited)**	**Severity score** (CU/SWU)	1.13 (1.10 to 1.15)	0.7052	1.10 (1.08 to 1.11)	0.7056
	**Age**	1.05 (1.05 to 1.05)		1.05 (1.04 to 1.05)	
	**Gender (F)**	1.04 (0.97 to 1.12)		1.06 (0.99 to 1.14)	
	**Deprivation** (Q5 vs. Q1)	1.78 (1.59 to 2.00)		1.77 (1.58 to 2.00)	
	**Ethnicity (vs. White)**				
	• Indian	0.70 (0.51 to 0.95)		0.70 (0.51 to 0.95)	
	• Unknown	0.20 (0.14 to 0.27)		0.20 (0.15 to 0.28)	
**Model 2 (10-year)**	**Severity score** (C10/SW10)	1.13 (1.10 to 1.15)	0.7042	1.10 (1.09 to 1.12)	0.7047
	**Age**	1.05 (1.05 to 1.05)		1.05 (1.05 to 1.05)	
	**Gender (F)**	1.03 (0.96 to 1.11)		1.05 (0.98 to 1.13)	
	**Deprivation** (Q5 vs. Q1)	1.78 (1.59 to 2.00)		1.77 (1.58 to 1.98)	
	**Ethnicity (vs. White)**				
	• Indian	0.70 (0.51 to 0.95)		0.70 (0.51 to 0.95)	
	• Unknown	0.20 (0.14 to 0.27)		0.20 (0.14 to 0.28)	
**Model 2 (5-year)**	**Severity score** (C5/SW5)	1.12 (1.09 to 1.15)	0.7026	1.11 (1.09 to 1.13)	0.7036
	**Age**	1.05 (1.05 to 1.05)		1.05 (1.05 to 1.05)	
	**Gender (F)**	1.02 (0.95 to 1.10)		1.04 (0.97 to 1.11)	
	**Deprivation** (Q5 vs. Q1)	1.79 (1.60 to 2.01)		1.78 (1.59 to 2.00)	
	**Ethnicity (vs. White)**				
	• Indian	0.70 (0.51 to 0.95)		0.70 (0.52 to 0.95)	
	• Unknown	0.19 (0.14 to 0.27)		0.20 (0.14 to 0.27)	
**Model 3**	**Age**	1.06 (1.05 to 1.06)	0.7104	-	
	**Gender (F)**	0.98 (0.90 to 1.06)			
	**Deprivation** (Q5 vs. Q1)	1.88 (1.65 to 2.14)			
	**Ethnicity (vs. White)**				
	• Indian	0.68 (0.47 to 0.97)			
	• Unknown	0.19 (0.14 to 0.27)			
	Baseline HbA_{1c}	1.17 (1.15 to 1.18)			
**Model 4 (unlimited)**	**Severity score** (CU/SWU)	1.15 (1.12 to 1.18)	0.7162	1.11 (1.09 to 1.13)	0.7163
	**Age**	1.05 (1.05 to 1.06)		1.05 (1.05 to 1.06)	
	**Gender (F)**	1.02 (0.94 to 1.10)		1.04 (0.96 to 1.13)	
	**Deprivation** (Q5 vs. Q1)	1.80 (1.58 to 2.05)		1.79 (1.57 to 2.04)	
	**Ethnicity (vs. White)**				
	• Indian	0.66 (0.47 to 0.95)		0.67 (0.47 to 0.95)	
	• Unknown	0.21 (0.15 to 0.29)		0.21 (0.15 to 0.30)	
	Baseline HbA_{1c}	1.18 (1.16 to 1.20)		1.18 (1.16 to 1.19)	
**Model 4 (10-year)**	**Severity score** (C10/SW10)	1.15 (1.12 to 1.18)	0.7151	1.12 (1.10 to 1.13)	0.7155
	**Age**	1.05 (1.05 to 1.06)		1.05 (1.05 to 1.06)	
	**Gender (F)**	1.01 (0.93 to 1.09)		1.03 (0.95 to 1.11)	
	**Deprivation** (Q5 vs. Q1)	1.81 (1.59 to 2.06)		1.79 (1.57 to 2.04)	
	**Ethnicity (vs. White)**				
	• Indian	0.66 (0.46 to 0.95)		0.66 (0.47 to 0.95)	
	• Unknown	0.21 (0.14 to 0.29)		0.21 (0.15 to 0.30)	
	Baseline HbA_{1c}	1.18 (1.16 to 1.19)		1.18 (1.16 to 1.19)	
**Model 4 (5-year)**	**Severity score** (C5/SW5)	1.14 (1.11 to 1.18)	0.7136	1.12 (1.10 to 1.15)	0.7144
	**Age**	1.06 (1.05 to 1.06)		1.05 (1.05 to 1.06)	
	**Gender (F)**	1.00 (0.92 to 1.08)		1.02 (0.94 to 1.10)	
	**Deprivation** (Q5 vs. Q1)	1.83 (1.60 to 2.08)		1.81 (1.59 to 2.06)	
	**Ethnicity (vs. White)**				
	• Indian	0.67 (0.47 to 0.95)		0.67 (0.47 to 1.00)	
	• Unknown	0.20 (0.14 to 0.29)		0.21 (0.14 to 0.29)	
	Baseline HbA_{1c}	1.18 (1.16 to 1.19)		1.17 (1.16 to 1.19)	

Zghebi SS, *et al. BMJ Open Diab Res Care* 2020; 8:e000962. doi: 10.1136/bmjdrc-2019-000962

Supplementary data to "Development and validation of the DIabetes Severity SCOre (DISSCO)". Zghebi et al.

Table S15 Hazard ratios (95% CI) for risk of clustered CV- and diabetes-related hospitalisation (without hypoglycemia) associated with simple count and severity-weighted scores estimated at three baseline windows - Training dataset

Model	Predictor(s)	Simple count (C) score		Severity-weighted (SW) score	
		HR (95% CI)	AUROC	HR (95% CI)	AUROC
**Model 1**	**Age**	1.03 (1.03 to 1.03)	0.6329	-	
	**Gender (F)**	0.96 (0.95 to 0.98)			
	**Deprivation** (Q5 vs. Q1)	1.29 (1.26 to 1.32)			
	**Ethnicity (vs. White)**				
	• Indian	0.97 (0.91 to 1.02)			
	• Unknown	0.19 (0.18 to 0.20)			
**Model 2 (unlimited)**	**Severity score** (CU/SWU)	1.14 (1.13 to 1.14)	0.6445	1.09 (1.09 to 1.10)	0.6433
	**Age**	1.02 (1.02 to 1.02)		1.02 (1.02 to 1.02)	
	**Gender (F)**	1.00 (0.98 to 1.01)		1.01 (1.00 to 1.03)	
	**Deprivation** (Q5 vs. Q1)	1.26 (1.23 to 1.29)		1.26 (1.23 to 1.29)	
	**Ethnicity (vs. White)**				
	• Indian	0.97 (0.92 to 1.02)		0.97 (0.91 to 1.02)	
	• Unknown	0.20 (0.19 to 0.21)		0.20 (0.19 to 0.21)	
**Model 2 (10-year)**	**Severity score** (C10/SW10)	1.14 (1.13 to 1.14)	0.6427	1.10 (1.09 to 1.10)	0.6422
	**Age**	1.02 (1.02 to 1.02)		1.02 (1.02 to 1.02)	
	**Gender (F)**	0.99 (0.97 to 1.00)		1.00 (0.99 to 1.02)	
	**Deprivation** (Q5 vs. Q1)	1.26 (1.23 to 1.29)		1.26 (1.23 to 1.29)	
	**Ethnicity (vs. White)**				
	• Indian	0.96 (0.91 to 1.02)		0.96 (0.91 to 1.02)	
	• Unknown	0.20 (0.19 to 0.21)		0.20 (0.19 to 0.21)	
**Model 2 (5-year)**	**Severity score** (C5/SW5)	1.14 (1.13 to 1.15)	0.6404	1.11 (1.10 to 1.11)	0.6404
	**Age**	1.02 (1.02 to 1.02)		1.02 (1.02 to 1.02)	
	**Gender (F)**	0.98 (0.97 to 1.00)		0.99 (0.98 to 1.01)	
	**Deprivation** (Q5 vs. Q1)	1.27 (1.24 to 1.30)		1.27 (1.24 to 1.30)	
	**Ethnicity (vs. White)**				
	• Indian	0.97 (0.92 to 1.02)		0.97 (0.92 to 1.02)	
	• Unknown	0.20 (0.19 to 0.21)		0.20 (0.19 to 0.21)	
**Model 3**	**Age**	1.03 (1.03 to 1.03)	0.6329	-	
	**Gender (F)**	0.96 (0.95 to 0.98)			
	**Deprivation** (Q5 vs. Q1)	1.29 (1.25 to 1.32)			
	**Ethnicity (vs. White)**				
	• Indian	0.94 (0.89 to 1.00)			
	• Unknown	0.18 (0.17 to 0.19)			
	Baseline HbA_{1c}	1.01 (1.00 to 1.01)			
**Model 4 (unlimited)**	**Severity score** (CU/SWU)	1.13 (1.13 to 1.14)	0.6442	1.09 (1.09 to 1.09)	0.6431
	**Age**	1.02 (1.02 to 1.02)		1.02 (1.02 to 1.02)	
	**Gender (F)**	1.00 (0.98 to 1.01)		1.01 (0.99 to 1.03)	
	**Deprivation** (Q5 vs. Q1)	1.25 (1.22 to 1.29)		1.25 (1.22 to 1.29)	
	**Ethnicity (vs. White)**				
	• Indian	0.94 (0.89 to 1.00)		0.94 (0.89 to 1.00)	
	• Unknown	0.19 (0.18 to 0.20)		0.19 (0.18 to 0.20)	
	Baseline HbA_{1c}	1.02 (1.01 to 1.02)		1.01 (1.01 to 1.02)	
**Model 4 (10-year)**	**Severity score** (C10/SW10)	1.14 (1.13 to 1.14)	0.6425	1.10 (1.09 to 1.10)	0.6420
	**Age**	1.02 (1.02 to 1.02)		1.02 (1.02 to 1.02)	
	**Gender (F)**	1.00 (0.97 to 1.00)		1.00 (0.98 to 1.02)	
	**Deprivation** (Q5 vs. Q1)	1.25 (1.22 to 1.29)		1.25 (1.22 to 1.29)	
	**Ethnicity (vs. White)**				
	• Indian	0.94 (0.88 to 1.00)		0.94 (0.89 to 1.00)	
	• Unknown	0.19 (0.18 to 0.20)		0.19 (0.18 to 0.20)	
	Baseline HbA_{1c}	1.02 (1.01 to 1.02)		1.01 (1.01 to 1.02)	
**Model 4 (5-year)**	**Severity score** (C5/SW5)	1.14 (1.13 to 1.15)	0.6404	1.10 (1.10 to 1.11)	0.6403
	**Age**	1.02 (1.02 to 1.02)		1.02 (1.02 to 1.02)	
	**Gender (F)**	0.98 (0.96 to 1.00)		0.99 (0.97 to 1.01)	
	**Deprivation** (Q5 vs. Q1)	1.26 (1.22 to 1.29)		1.26 (1.23 to 1.29)	
	**Ethnicity (vs. White)**				
	• Indian	0.94 (0.89 to 1.00)		0.94 (0.89 to 1.00)	
	• Unknown	0.19 (0.18 to 0.20)		0.19 (0.18 to 0.20)	
	Baseline HbA_{1c}	1.01 (1.01 to 1.02)		1.01 (1.01 to 1.02)	

Zghebi SS, *et al. BMJ Open Diab Res Care* 2020; 8:e000962. doi: 10.1136/bmjdrc-2019-000962

Supplementary data to "Development and validation of the DIabetes Severity SCOre (DISSCO)". Zghebi et al.

Table S16 Hazard ratios (95% CI) for risk of cardiovascular interventions (OPCS) associated with simple count and severity-weighted scores estimated at three baseline windows - Training dataset

Model	Predictor(s)	Simple count (C) score		Severity-weighted (SW) score	
		HR (95% CI)	AUROC	HR (95% CI)	AUROC
**Model 1**	**Age**	1.02 (1.02 to 1.02)	0.6665		
	**Gender (F)**	0.46 (0.44 to 0.49)			
	**Deprivation** (Q5 vs. Q1)	1.23 (1.13 to 1.34)		-	
	**Ethnicity (vs. White)**				
	• Indian	1.09 (0.91 to 1.31)			
	• Unknown	0.13 (0.10 to 0.16)			
**Model 2 (unlimited)**	**Severity score** (CU/SWU)	1.30 (1.28 to 1.32)	0.6858	1.16 (1.15 to 1.17)	0.6778
	**Age**	1.01 (1.01 to 1.01)		1.01 (1.01 to 1.01)	
	**Gender (F)**	0.51 (0.48 to 0.54)		0.51 (0.48 to 0.54)	
	**Deprivation** (Q5 vs. Q1)	1.15 (1.05 to 1.25)		1.15 (1.06 to 1.26)	
	**Ethnicity (vs. White)**				
	• Indian	1.07 (0.89 to 1.29)		1.08 (0.90 to 1.30)	
	• Unknown	0.15 (0.11 to 0.19)		0.14 (0.11 to 0.19)	
**Model 2 (10-year)**	**Severity score** (C10/SW10)	1.32 (1.30 to 1.34)	0.6850	1.17 (1.16 to 1.18)	0.6773
	**Age**	1.01 (1.01 to 1.01)		1.01 (1.01 to 1.01)	
	**Gender (F)**	0.50 (0.47 to 0.53)		0.50 (0.47 to 0.53)	
	**Deprivation** (Q5 vs. Q1)	1.14 (1.05 to 1.25)		1.15 (1.06 to 1.26)	
	**Ethnicity (vs. White)**				
	• Indian	1.07 (0.89 to 1.29)		1.08 (0.90 to 1.30)	
	• Unknown	0.14 (0.12 to 0.19)		0.14 (0.11 to 0.18)	
**Model 2 (5-year)**	**Severity score** (C5/SW5)	1.35 (1.33 to 1.38)	0.6833	1.19 (1.18 to 1.21)	0.6759
	**Age**	1.01 (1.01 to 1.02)		1.01 (1.01 to 1.01)	
	**Gender (F)**	0.49 (0.46 to 0.52)		0.49 (0.46 to 0.52)	
	**Deprivation** (Q5 vs. Q1)	1.15 (1.05 to 1.25)		1.16 (1.06 to 1.27)	
	**Ethnicity (vs. White)**				
	• Indian	1.08 (0.90 to 1.29)		1.08 (0.90 to 1.30)	
	• Unknown	0.14 (0.11 to 0.18)		0.14 (0.11 to 0.18)	
**Model 3**	**Age**	1.02 (1.02 to 1.02)	0.6723		
	**Gender (F)**	0.45 (0.42 to 0.48)			
	**Deprivation** (Q5 vs. Q1)	1.20 (1.09 to 1.32)			
	**Ethnicity (vs. White)**			-	
	• Indian	1.06 (0.86 to 1.21)			
	• Unknown	0.12 (0.10 to 0.16)			
	Baseline HbA_{1c}	1.02 (1.01 to 1.04)			
**Model 4 (unlimited)**	**Severity score** (CU/SWU)	1.31 (1.29 to 1.33)	0.6903	1.16 (1.15 to 1.17)	0.6825
	**Age**	1.01 (1.01 to 1.01)		1.01 (1.01 to 1.02)	
	**Gender (F)**	0.50 (0.47 to 0.54)		0.50 (0.47 to 0.54)	
	**Deprivation** (Q5 vs. Q1)	1.10 (1.00 to 1.22)		1.12 (1.01 to 1.23)	
	**Ethnicity (vs. White)**				
	• Indian	1.04 (0.85 to 1.29)		1.05 (0.85 to 1.30)	
	• Unknown	0.14 (0.11 to 0.19)		0.14 (0.10 to 0.18)	
	Baseline HbA_{1c}	1.05 (1.03 to 1.06)		1.04 (1.02 to 1.05)	
**Model 4 (10-year)**	**Severity score** (C10/SW10)	1.33 (1.31 to 1.35)	0.6901	1.17 (1.16 to 1.19)	0.6823
	**Age**	1.01 (1.01 to 1.02)		1.01 (1.01 to 1.02)	
	**Gender (F)**	0.49 (0.46 to 0.53)		0.49 (0.46 to 0.53)	
	**Deprivation** (Q5 vs. Q1)	1.10 (1.00 to 1.21)		1.12 (1.01 to 1.23)	
	**Ethnicity (vs. White)**				
	• Indian	1.04 (0.85 to 1.29)		1.05 (0.85 to 1.30)	
	• Unknown	0.14 (0.10 to 0.19)		0.14 (0.10 to 0.18)	
	Baseline HbA_{1c}	1.05 (1.03 to 1.06)		1.04 (1.02 to 1.05)	
**Model 4 (5-year)**	**Severity score** (C5/SW5)	1.36 (1.34 to 1.39)	0.6885	1.19 (1.18 to 1.21)	0.6809
	**Age**	1.02 (1.01 to 1.02)		1.02 (1.01 to 1.02)	
	**Gender (F)**	0.48 (0.45 to 0.52)		0.49 (0.46 to 0.52)	
	**Deprivation** (Q5 vs. Q1)	1.11 (1.01 to 1.22)		1.12 (1.02 to 1.24)	
	**Ethnicity (vs. White)**				
	• Indian	1.05 (0.85 to 1.30)		1.06 (0.86 to 1.31)	
	• Unknown	0.14 (0.10 to 0.18)		0.14 (0.10 to 0.18)	
	Baseline HbA_{1c}	1.04 (1.03 to 1.06)		1.03 (1.02 to 1.05)	

Supplementary data to "Development and validation of the DIabetes Severity SCOre (DISSCO)". Zghebi et al.

Table S17: Calibration test for simple count and severity-weighted scores in training and validation datasets using Somer's D

Somer's D	Training dataset						Validation dataset					
	**CU**	**SWU**	**C10**	**SW10**	**C5**	**SW5**	**CU**	**SWU**	**C10**	**SW10**	**C5**	**SW5**
All-cause mortality	0.2079	0.1770	0.1852	0.1598	0.1528	0.1350	0.2197	0.1841	0.1959	0.1677	0.1656	0.1427
Any cause hospitalisation	0.1229	0.1080	0.1128	0.0996	0.0957	0.0857	0.1279	0.1110	0.1172	0.1024	0.1019	0.0899
CV-related hospitalisation	0.3090	0.2349	0.2903	0.2175	0.2559	0.1888	0.3069	0.2322	0.2895	0.2162	0.2571	0.1858
Diabetes-related hospitalisation	0.1261	0.1104	0.1153	0.1017	0.0980	0.0876	0.1247	0.1093	0.1135	0.1012	0.0978	0.0888
CV/DM hosp	0.1534	0.1321	0.1402	0.1213	0.1201	0.1047	0.1554	0.1330	0.1423	0.1231	0.1238	0.1075
Hypoglycaemia-hospitalisation	0.1677	0.1518	0.1514	0.1375	0.1243	0.1168	0.1757	0.1569	0.1606	0.1463	0.1397	0.1282

Simple count (C) score measured using unlimited (**CU**), 10-yr (**C10**), and 5-yr (**C5**) look-back windows.
Severity-weighted (SW) score measured using unlimited (**SWU**), 10-yr (**SW10**), and 5-yr (**SW5**) look-back windows.

Zghebi SS, *et al. BMJ Open Diab Res Care* 2020; 8:e000962. doi: 10.1136/bmjdrc-2019-000962

Supplementary data to "Development and validation of the DIabetes Severity SCOre (DISSCO)". Zghebi et al.

Table S18 Hazard ratios (95% CI) for risk of all-cause mortality associated with simple count and severity-weighted scores estimated at three baseline windows - validation dataset

Model	Predictor(s)	Simple count (C) score		Severity-weighted (SW) score	
		HR (95% CI)	AUROC	HR (95% CI)	AUROC
**Model 1**	**Age**	1.10 (1.10 to 1.10)	0.7586	-	
	**Gender (F)**	0.76 (0.72 to 0.80)			
	**Deprivation** (Q5 vs. Q1)	1.13 (1.03 to 1.23)			
	**Ethnicity (vs. White)**				
	• Indian	0.72 (0.55 to 0.95)			
	• Unknown	0.93 (0.82 to 1.06)			
**Model 2 (unlimited)**	**Severity score** (CU/SWU)	1.16 (1.14 to 1.18)	0.7638	1.11 (1.10 to 1.12)	0.7622
	**Age**	1.09 (1.09 to 1.10)		1.09 (1.09 to 1.10)	
	**Gender (F)**	0.79 (0.75 to 0.84)		0.82 (0.78 to 0.87)	
	**Deprivation** (Q5 vs. Q1)	1.42 (1.29 to 1.55)		1.41 (1.29 to 1.54)	
	**Ethnicity (vs. White)**				
	• Indian	0.70 (0.53 to 0.92)		0.70 (0.53 to 0.92)	
	• Unknown	1.01 (0.89 to 1.14)		1.02 (0.90 to 1.15)	
**Model 2 (10-year)**	**Severity score** (C10/SW10)	1.16 (1.14 to 1.18)	0.7633	1.12 (1.11 to 1.13)	0.7622
	**Age**	1.10 (1.09 to 1.10)		1.09 (1.09 to 1.10)	
	**Gender (F)**	0.79 (0.74 to 0.83)		0.81 (0.77 to 0.85)	
	**Deprivation** (Q5 vs. Q1)	1.42 (1.30 to 1.55)		1.41 (1.29 to 1.55)	
	**Ethnicity (vs. White)**				
	• Indian	0.71 (0.54 to 0.93)		0.70 (0.54 to 0.93)	
	• Unknown	1.00 (0.88 to 1.13)		1.01 (0.90 to 1.15)	
**Model 2 (5-year)**	**Severity score** (C5/SW5)	1.16 (1.13 to 1.18)	0.7618	1.13 (1.11 to 1.14)	0.7611
	**Age**	1.10 (1.09 to 1.10)		1.10 (1.09 to 1.10)	
	**Gender (F)**	0.78 (0.73 to 0.82)		0.80 (0.75 to 0.84)	
	**Deprivation** (Q5 vs. Q1)	1.42 (1.30 to 1.55)		1.42 (1.30 to 1.55)	
	**Ethnicity (vs. White)**				
	• Indian	0.72 (0.55 to 0.95)		0.72 (0.55 to 0.95)	
	• Unknown	0.98 (0.86 to 1.11)		0.99 (0.88 to 1.13)	
**Model 3**	**Age**	1.10 (1.10 to 1.10)	0.7574	-	
	**Gender (F)**	0.74 (0.70 to 0.79)			
	**Deprivation** (Q5 vs. Q1)	1.47 (1.33 to 1.63)			
	**Ethnicity (vs. White)**				
	• Indian	0.64 (0.46 to 0.89)			
	• Unknown	0.86 (0.75 to 0.99)			
	Baseline HbA_{1c}	1.02 (1.01 to 1.04)			
**Model 4 (unlimited)**	**Severity score** (CU/SWU)	1.16 (1.14 to 1.18)	0.7620	1.11 (1.10 to 1.12)	0.7604
	**Age**	1.09 (1.09 to 1.10)		1.09 (1.09 to 1.10)	
	**Gender (F)**	0.78 (0.73 to 0.83)		0.80 (0.75 to 0.85)	
	**Deprivation** (Q5 vs. Q1)	1.43 (1.30 to 1.58)		1.42 (1.29 to 1.57)	
	**Ethnicity (vs. White)**				
	• Indian	0.62 (0.45 to 0.87)		0.62 (0.44 to 0.87)	
	• Unknown	0.93 (0.81 to 1.07)		0.94 (0.81 to 1.08)	
	Baseline HbA_{1c}	1.04 (1.02 to 1.05)		1.04 (1.02 to 1.05)	
**Model 4 (10-year)**	**Severity score** (C10/SW10)	1.16 (1.14 to 1.18)	0.7617	1.12 (1.11 to 1.13)	0.7606
	**Age**	1.10 (1.09 to 1.10)		1.09 (1.09 to 1.10)	
	**Gender (F)**	0.77 (0.73 to 0.82)		0.79 (0.74 to 0.84)	
	**Deprivation** (Q5 vs. Q1)	1.44 (1.30 to 1.58)		1.43 (1.30 to 1.58)	
	**Ethnicity (vs. White)**				
	• Indian	0.63 (0.45 to 0.88)		0.63 (0.45 to 0.87)	
	• Unknown	0.92 (0.80 to 1.06)		0.94 (0.82 to 1.08)	
	Baseline HbA_{1c}	1.04 (1.02 to 1.05)		1.04 (1.02 to 1.05)	
**Model 4 (5-year)**	**Severity score** (C5/SW5)	1.16 (1.13 to 1.18)	0.7604	1.12 (1.11 to 1.14)	0.7597
	**Age**	1.10 (1.09 to 1.10)		1.10 (1.09 to 1.10)	
	**Gender (F)**	0.76 (0.71 to 0.81)		0.77 (0.73 to 0.82)	
	**Deprivation** (Q5 vs. Q1)	1.43 (1.30 to 1.58)		1.43 (1.30 to 1.58)	
	**Ethnicity (vs. White)**				
	• Indian	0.64 (0.46 to 0.89)		0.64 (0.46 to 0.89)	
	• Unknown	0.91 (0.79 to 1.04)		0.92 (0.80 to 1.06)	
	Baseline HbA_{1c}	1.03 (1.02 to 1.05)		1.03 (1.02 to 1.05)	

Zghebi SS, *et al. BMJ Open Diab Res Care* 2020; 8:e000962. doi: 10.1136/bmjdrc-2019-000962

Supplementary data to "Development and validation of the DIabetes Severity SCOre (DISSCO)". Zghebi et al.

Table S19 Hazard ratios (95% CI) for risk of all-cause hospitalisation associated with simple count and severity-weighted scores estimated at three baseline windows - validation dataset

Model	Predictor(s)	Simple count (C) score		Severity-weighted (SW) score	
		HR (95% CI)	AUROC	HR (95% CI)	AUROC
**Model 1**	**Age**	1.02 (1.02 to 1.03)	0.6248	-	
	**Gender (F)**	1.00 (0.98 to 1.03)			
	**Deprivation** (Q5 vs. Q1)	1.17 (1.12 to 1.22)			
	**Ethnicity (vs. White)**				
	• Indian	1.05 (0.96 to 1.16)			
	• Unknown	0.21 (0.19 to 0.23)			
**Model 2 (unlimited)**	**Severity score** (CU/SWU)	1.10 (1.09 to 1.11)	0.6323	1.07 (1.06 to 1.08)	0.6323
	**Age**	1.02 (1.02 to 1.02)		1.02 (1.02 to 1.02)	
	**Gender (F)**	1.03 (1.00 to 1.06)		1.04 (1.01 to 1.07)	
	**Deprivation** (Q5 vs. Q1)	1.15 (1.10 to 1.20)		1.15 (1.10 to 1.20)	
	**Ethnicity (vs. White)**				
	• Indian	1.06 (0.96 to 1.16)		1.06 (0.96 to 1.16)	
	• Unknown	0.21 (0.20 to 0.23)		0.21 (0.20 to 0.23)	
**Model 2 (10-year)**	**Severity score** (C10/SW10)	1.10 (1.09 to 1.11)	0.6312	1.07 (1.07 to 1.08)	0.6315
	**Age**	1.02 (1.02 to 1.02)		1.02 (1.02 to 1.02)	
	**Gender (F)**	1.02 (0.99 to 1.05)		1.03 (1.01 to 1.06)	
	**Deprivation** (Q5 vs. Q1)	1.15 (1.10 to 1.21)		1.15 (1.10 to 1.20)	
	**Ethnicity (vs. White)**				
	• Indian	1.06 (0.96 to 1.16)		1.06 (0.96 to 1.16)	
	• Unknown	0.21 (0.20 to 0.23)		0.21 (0.20 to 0.23)	
**Model 2 (5-year)**	**Severity score** (C5/SW5)	1.10 (1.09 to 1.11)	0.6297	1.08 (1.07 to 1.09)	0.6305
	**Age**	1.02 (1.02 to 1.02)		1.02 (1.02 to 1.02)	
	**Gender (F)**	1.02 (0.99 to 1.05)		1.03 (1.00 to 1.06)	
	**Deprivation** (Q5 vs. Q1)	1.16 (1.10 to 1.21)		1.15 (1.10 to 1.21)	
	**Ethnicity (vs. White)**				
	• Indian	1.06 (0.96 to 1.16)		1.06 (0.96 to 1.16)	
	• Unknown	0.21 (0.19 to 0.23)		0.21 (0.20 to 0.23)	
**Model 3**	**Age**	1.02 (1.02 to 1.03)	0.6261	-	
	**Gender (F)**	1.01 (0.97 to 1.03)			
	**Deprivation** (Q5 vs. Q1)	1.19 (1.13 to 1.24)			
	**Ethnicity (vs. White)**				
	• Indian	1.07 (0.96 to 1.19)			
	• Unknown	0.20 (0.18 to 0.22)			
	Baseline HbA_{1c}	0.99 (0.99 to 1.00)			
**Model 4 (unlimited)**	**Severity score** (CU/SWU)	1.09 (1.08 to 1.10)	0.6330	1.07 (1.06 to 1.07)	0.6330
	**Age**	1.02 (1.02 to 1.02)		1.02 (1.02 to 1.02)	
	**Gender (F)**	1.03 (1.00 to 1.06)		1.04 (1.01 to 1.07)	
	**Deprivation** (Q5 vs. Q1)	1.16 (1.10 to 1.22)		1.16 (1.10 to 1.22)	
	**Ethnicity (vs. White)**				
	• Indian	1.07 (0.96 to 1.19)		1.06 (0.96 to 1.19)	
	• Unknown	0.21 (0.19 to 0.23)		0.21 (0.19 to 0.23)	
	Baseline HbA_{1c}	1.00 (0.99 to 1.01)		1.00 (0.99 to 1.00)	
**Model 4 (10-year)**	**Severity score** (C10/SW10)	1.09 (1.08 to 1.11)	0.6320	1.07 (1.06 to 1.08)	0.6323
	**Age**	1.02 (1.02 to 1.02)		1.02 (1.02 to 1.02)	
	**Gender (F)**	1.02 (0.99 to 1.05)		1.03 (1.00 to 1.06)	
	**Deprivation** (Q5 vs. Q1)	1.16 (1.10 to 1.22)		1.16 (1.10 to 1.22)	
	**Ethnicity (vs. White)**				
	• Indian	1.07 (0.96 to 1.19)		1.07 (0.96 to 1.19)	
	• Unknown	0.21 (0.19 to 0.22)		0.21 (0.19 to 0.23)	
	Baseline HbA_{1c}	1.00 (0.99 to 1.01)		1.00 (0.99 to 1.00)	
**Model 4 (5-year)**	**Severity score** (C5/SW5)	1.10 (1.08 to 1.11)	0.6307	1.08 (1.07 to 1.09)	0.6315
	**Age**	1.02 (1.02 to 1.02)		1.02 (1.02 to 1.02)	
	**Gender (F)**	1.01 (0.98 to 1.05)		1.02 (0.99 to 1.06)	
	**Deprivation** (Q5 vs. Q1)	1.16 (1.11 to 1.22)		1.16 (1.11 to 1.22)	
	**Ethnicity (vs. White)**				
	• Indian	1.06 (0.95 to 1.19)		1.06 (0.95 to 1.19)	
	• Unknown	0.20 (0.19 to 0.22)		0.21 (0.19 to 0.22)	
	Baseline HbA_{1c}	1.00 (0.99 to 1.00)		1.00 (0.99 to 1.03)	

Zghebi SS, *et al. BMJ Open Diab Res Care* 2020; 8:e000962. doi: 10.1136/bmjdrc-2019-000962

Supplementary data to "Development and validation of the DIabetes Severity SCOre (DISSCO)". Zghebi et al.

Table S20 Hazard ratios (95% CI) for risk of cardiovascular-related hospitalisation associated with simple count and severity-weighted scores estimated at three baseline windows- validation dataset

Model	Predictor(s)	Simple count (C) score		Severity-weighted (SW) score	
		HR (95% CI)	AUROC	HR (95% CI)	AUROC
**Model 1**	**Age**	1.06 (1.06 to 1.06)	0.7063	-	
	**Gender (F)**	0.70 (0.67 to 0.73)			
	**Deprivation** (Q5 vs. Q1)	1.45 (1.36 to 1.55)			
	**Ethnicity (vs. White)**				
	• Indian	1.02 (0.87 to 1.19)			
	• Unknown	0.19 (0.17 to 0.23)			
**Model 2 (unlimited)**	**Severity score** (CU/SWU)	1.41 (1.40 to 1.43)	0.7317	1.22 (1.21 to 1.22)	0.7212
	**Age**	1.04 (1.04 to 1.05)		1.05 (1.04 to 1.05)	
	**Gender (F)**	0.79 (0.75 to 0.82)		0.80 (0.77 to 0.84)	
	**Deprivation** (Q5 vs. Q1)	1.35 (1.27 to 1.45)		1.35 (1.27 to 1.45)	
	**Ethnicity (vs. White)**				
	• Indian	1.03 (0.88 to 1.20)		1.04 (0.89 to 1.21)	
	• Unknown	0.22 (0.19 to 0.26)		0.22 (0.18 to 0.26)	
**Model 2 (10-year)**	**Severity score** (C10/SW10)	1.44 (1.42 to 1.46)	0.7309	1.23 (1.23 to 1.24)	0.7207
	**Age**	1.05 (1.05 to 1.05)		1.05 (1.05 to 1.05)	
	**Gender (F)**	0.76 (0.73 to 0.80)		0.78 (0.75 to 0.82)	
	**Deprivation** (Q5 vs. Q1)	1.35 (1.26 to 1.44)		1.36 (1.27 to 1.45)	
	**Ethnicity (vs. White)**				
	• Indian	1.04 (0.89 to 1.21)		1.05 (0.90 to 1.23)	
	• Unknown	0.22 (0.19 to 0.26)		0.22 (0.19 to 0.26)	
**Model 2 (5-year)**	**Severity score** (C5/SW5)	1.46 (1.44 to 1.48)	0.7267	1.25 (1.24 to 1.26)	0.7177
	**Age**	1.05 (1.05 to 1.05)		1.05 (1.05 to 1.05)	
	**Gender (F)**	0.74 (0.71 to 0.77)		0.76 (0.73 to 0.79)	
	**Deprivation** (Q5 vs. Q1)	1.37 (1.28 to 1.46)		1.38 (1.29 to 1.48)	
	**Ethnicity (vs. White)**				
	• Indian	1.04 (0.89 to 1.22)		1.05 (0.90 to 1.22)	
	• Unknown	0.21 (0.18 to 0.25)		0.21 (0.18 to 0.25)	
**Model 3**	**Age**	1.06 (1.06 to 1.06)	0.7093	-	
	**Gender (F)**	0.70 (0.66 to 0.73)			
	**Deprivation** (Q5 vs. Q1)	1.47 (1.37 to 1.58)			
	**Ethnicity (vs. White)**				
	• Indian	1.05 (0.88 to 1.25)			
	• Unknown	0.18 (0.15 to 0.22)			
	Baseline HbA_{1c}	0.98 (0.97 to 0.99)			
**Model 4 (unlimited)**	**Severity score** (CU/SWU)	1.41 (1.39 to 1.43)	0.7335	1.21 (1.21 to 1.22)	0.7232
	**Age**	1.04 (1.04 to 1.05)		1.05 (1.04 to 1.05)	
	**Gender (F)**	0.79 (0.76 to 0.83)		0.80 (0.77 to 0.84)	
	**Deprivation** (Q5 vs. Q1)	1.36 (1.26 to 2.88)		1.36 (1.26 to 1.46)	
	**Ethnicity (vs. White)**				
	• Indian	1.06 (0.89 to 1.26)		1.07 (0.90 to 1.27)	
	• Unknown	0.21 (0.17 to 0.25)		0.20 (0.17 to 0.24)	
	Baseline HbA_{1c}	1.01 (1.00 to 1.02)		1.00 (0.99 to 1.01)	
**Model 4 (10-year)**	**Severity score** (C10/SW10)	1.44 (1.42 to 1.46)	0.7326	1.23 (1.22 to 1.24)	0.7227
	**Age**	1.05 (1.05 to 1.05)		1.05 (1.05 to 1.05)	
	**Gender (F)**	0.77 (0.73 to 0.80)		0.78 (0.75 to 0.82)	
	**Deprivation** (Q5 vs. Q1)	1.35 (1.26 to 1.46)		1.37 (1.27 to 1.47)	
	**Ethnicity (vs. White)**				
	• Indian	1.06 (0.89 to 1.26)		1.08 (0.90 to 1.28)	
	• Unknown	0.21 (0.17 to 0.25)		0.21 (0.17 to 0.25)	
	Baseline HbA_{1c}	1.01 (1.00 to 1.02)		1.00 (0.99 to 1.01)	
**Model 4 (5-year)**	**Severity score** (C5/SW5)	1.46 (1.44 to 1.48)	0.7290	1.25 (1.24 to 1.26)	0.7201
	**Age**	1.05 (1.05 to 1.05)		1.05 (1.05 to 1.05)	
	**Gender (F)**	0.74 (0.71 to 0.78)		0.76 (0.72 to 0.79)	
	**Deprivation** (Q5 vs. Q1)	1.37 (1.27 to 1.47)		1.39 (1.29 to 1.49)	
	**Ethnicity (vs. White)**				
	• Indian	1.06 (0.89 to 1.26)		1.08 (0.90 to 1.28)	
	• Unknown	0.20 (0.17 to 0.24)		0.20 (0.17 to 0.24)	
	Baseline HbA_{1c}	1.01 (1.00 to 1.02)		1.00 (0.99 to 1.01)	

Supplementary data to "Development and validation of the DIabetes Severity SCOre (DISSCO)". Zghebi et al.

Table S21 Hazard ratios (95% CI) for risk of diabetes-related hospitalisation associated with simple count and severity-weighted scores estimated at three baseline windows- validation dataset

Model	Predictor(s)	Simple count (C) score		Severity-weighted (SW) score	
		HR (95% CI)	AUROC	HR (95% CI)	AUROC
**Model 1**	**Age**	1.02 (1.02 to 1.03)	0.6244	-	
	**Gender (F)**	1.00 (0.97 to 1.03)			
	**Deprivation** (Q5 vs. Q1)	1.23 (1.17 to 1.29)			
	**Ethnicity (vs. White)**				
	• Indian	0.98 (0.89 to 1.09)			
	• Unknown	0.21 (0.19 to 0.23)			
**Model 2 (unlimited)**	**Severity score** (CU/SWU)	1.10 (1.09 to 1.11)	0.6316	1.07 (1.06 to 1.08)	0.6318
	**Age**	1.02 (1.02 to 1.03)		1.02 (1.02 to 1.02)	
	**Gender (F)**	1.02 (1.00 to 1.06)		1.04 (1.01 to 1.07)	
	**Deprivation** (Q5 vs. Q1)	1.20 (1.14 to 1.26)		1.20 (1.14 to 1.26)	
	**Ethnicity (vs. White)**				
	• Indian	0.98 (0.88 to 1.09)		0.98 (0.88 to 1.09)	
	• Unknown	0.21 (0.19 to 0.23)		0.21 (0.19 to 0.23)	
**Model 2 (10-year)**	**Severity score** (C10/SW10)	1.10 (1.08 to 1.11)	0.6305	1.07 (1.06 to 1.08)	0.6312
	**Age**	1.02 (1.02 to 1.02)		1.02 (1.02 to 1.02)	
	**Gender (F)**	1.02 (0.99 to 1.05)		1.03 (1.00 to 1.07)	
	**Deprivation** (Q5 vs. Q1)	1.20 (1.14 to 1.26)		1.20 (1.14 to 1.26)	
	**Ethnicity (vs. White)**				
	• Indian	0.98 (0.88 to 1.09)		0.98 (0.88 to 1.09)	
	• Unknown	0.21 (0.19 to 0.23)		0.21 (0.19 to 0.23)	
**Model 2 (5-year)**	**Severity score** (C5/SW5)	1.10 (1.08 to 1.11)	0.6290	1.08 (1.07 to 1.09)	0.6300
	**Age**	1.02 (1.02 to 1.02)		1.02 (1.02 to 1.02)	
	**Gender (F)**	1.02 (0.99 to 1.05)		1.03 (1.00 to 1.06)	
	**Deprivation** (Q5 vs. Q1)	1.21 (1.15 to 1.27)		1.20 (1.15 to 1.27)	
	**Ethnicity (vs. White)**				
	• Indian	0.98 (0.89 to 1.09)		0.99 (0.89 to 1.10)	
	• Unknown	0.21 (0.19 to 0.23)		0.21 (0.19 to 0.23)	
**Model 3**	**Age**	1.02 (1.02 to 1.03)	0.6251	-	
	**Gender (F)**	1.01 (0.97 to 1.04)			
	**Deprivation** (Q5 vs. Q1)	1.25 (1.18 to 1.32)			
	**Ethnicity (vs. White)**				
	• Indian	1.03 (0.92 to 1.16)			
	• Unknown	0.20 (0.18 to 0.22)			
	Baseline HbA_{1c}	1.01 (1.01 to 1.02)			
**Model 4 (unlimited)**	**Severity score** (CU/SWU)	1.09 (1.08 to 1.11)	0.6320	1.07 (1.06 to 1.07)	0.6322
	**Age**	1.02 (1.02 to 1.02)		1.02 (1.02 to 1.02)	
	**Gender (F)**	1.03 (1.00 to 1.07)		1.04 (1.01 to 1.08)	
	**Deprivation** (Q5 vs. Q1)	1.22 (1.16 to 1.28)		1.22 (1.15 to 1.28)	
	**Ethnicity (vs. White)**				
	• Indian	1.03 (0.91 to 1.16)		1.03 (0.91 to 1.15)	
	• Unknown	0.20 (0.18 to 0.22)		0.20 (0.18 to 0.22)	
	Baseline HbA_{1c}	1.02 (1.01 to 1.03)		1.02 (1.01 to 1.02)	
**Model 4 (10-year)**	**Severity score** (C10/SW10)	1.10 (1.08 to 1.11)	0.6311	1.07 (1.06 to 1.08)	0.6316
	**Age**	1.02 (1.02 to 1.02)		1.02 (1.02 to 1.02)	
	**Gender (F)**	1.03 (0.99 to 1.06)		1.04 (1.00 to 1.07)	
	**Deprivation** (Q5 vs. Q1)	1.22 (1.16 to 1.29)		1.22 (1.16 to 1.29)	
	**Ethnicity (vs. White)**				
	• Indian	1.03 (0.91 to 1.16)		1.03 (0.91 to 1.15)	
	• Unknown	0.20 (0.18 to 0.22)		0.20 (0.18 to 0.22)	
	Baseline HbA_{1c}	1.02 (1.01 to 1.03)		1.02 (1.01 to 1.02)	
**Model 4 (5-year)**	**Severity score** (C5/SW5)	1.10 (1.08 to 1.11)	0.6297	1.08 (1.07 to 1.09)	0.6306
	**Age**	1.02 (1.02 to 1.02)		1.02 (1.02 to 1.02)	
	**Gender (F)**	1.02 (0.99 to 1.05)		1.03 (1.00 to 1.06)	
	**Deprivation** (Q5 vs. Q1)	1.22 (1.16 to 1.29)		1.22 (1.16 to 1.29)	
	**Ethnicity (vs. White)**				
	• Indian	1.03 (0.91 to 1.16)		1.03 (0.92 to 1.16)	
	• Unknown	0.20 (0.18 to 0.22)		0.20 (0.18 to 0.22)	
	Baseline HbA_{1c}	1.02 (1.01 to 1.02)		1.02 (1.01 to 1.02)	

Zghebi SS, *et al. BMJ Open Diab Res Care* 2020; 8:e000962. doi: 10.1136/bmjdrc-2019-000962

Supplementary data to "Development and validation of the DIabetes Severity SCOre (DISSCO)". Zghebi et al.

Table S22 Hazard ratios (95% CI) for risk of hospitalisation due to hypoglycaemia associated with simple count and severity-weighted scores estimated at three baseline windows (secondary outcome) - validation dataset

Model	Predictor(s)	Simple count (C) score		Severity-weighted (SW) score	
		HR (95% CI)	AUROC	HR (95% CI)	AUROC
**Model 1**	**Age**	1.05 (1.05 to 1.06)	0.7038	-	
	**Gender (F)**	1.03 (0.90 to 1.19)			
	**Deprivation** (Q5 vs. Q1)	1.86 (1.48 to 2.33)			
	**Ethnicity (vs. White)**				
	• Indian	1.15 (0.72 to 1.84)			
	• Unknown	0.18 (0.09 to 0.35)			
**Model 2 (unlimited)**	**Severity score** (CU/SWU)	1.14 (1.09 to 1.19)	0.7106	1.11 (1.08 to 1.14)	0.7113
	**Age**	1.05 (1.04 to 1.05)		1.05 (1.04 to 1.05)	
	**Gender (F)**	1.07 (0.93 to 1.23)		1.10 (0.96 to 1.27)	
	**Deprivation** (Q5 vs. Q1)	1.81 (1.45 to 2.27)		1.80 (1.43 to 2.25)	
	**Ethnicity (vs. White)**				
	• Indian	1.12 (0.70 to 1.79)		1.12 (0.70 to 1.79)	
	• Unknown	0.19 (0.10 to 0.37)		0.19 (0.10 to 0.37)	
**Model 2 (10-year)**	**Severity score** (C10/SW10)	1.15 (1.10 to 1.20)	0.7098	1.12 (1.09 to 1.15)	0.7109
	**Age**	1.05 (1.04 to 1.06)		1.05 (1.04 to 1.05)	
	**Gender (F)**	1.06 (0.93 to 1.22)		1.09 (0.95 to 1.26)	
	**Deprivation** (Q5 vs. Q1)	1.81 (1.45 to 2.27)		1.79 (1.43 to 2.25)	
	**Ethnicity (vs. White)**				
	• Indian	1.12 (0.70 to 1.80)		1.12 (0.70 to 1.79)	
	• Unknown	0.19 (0.10 to 0.37)		0.19 (0.10 to 0.37)	
**Model 2 (5-year)**	**Severity score** (C5/SW5)	1.15 (1.10 to 1.21)	0.7080	1.13 (1.10 to 1.17)	0.7093
	**Age**	1.05 (1.04 to 1.06)		1.05 (1.04 to 1.06)	
	**Gender (F)**	1.05 (0.92 to 1.21)		1.08 (0.93 to 1.24)	
	**Deprivation** (Q5 vs. Q1)	1.81 (1.45 to 2.27)		1.80 (1.44 to 2.25)	
	**Ethnicity (vs. White)**				
	• Indian	1.15 (0.72 to 1.83)		1.14 (0.71 to 1.83)	
	• Unknown	0.19 (0.10 to 0.36)		0.19 (0.10 to 0.37)	
**Model 3**	**Age**	1.06 (1.05 to 1.06)	0.7159	-	
	**Gender (F)**	1.02 (0.87 to 1.19)			
	**Deprivation** (Q5 vs. Q1)	1.95 (1.51 to 2.52)			
	**Ethnicity (vs. White)**				
	• Indian	1.38 (0.81 to 2.36)			
	• Unknown	0.16 (0.08 to 0.35)			
	Baseline HbA_{1c}	1.16 (1.13 to 1.19)			
**Model 4 (unlimited)**	**Severity score** (CU/SWU)	1.17 (1.12 to 1.22)	0.7232	1.12 (1.09 to 1.15)	0.7236
	**Age**	1.05 (1.04 to 1.06)		1.05 (1.04 to 1.06)	
	**Gender (F)**	1.07 (0.91 to 1.24)		1.10 (0.94 to 1.29)	
	**Deprivation** (Q5 vs. Q1)	1.88 (1.45 to 2.43)		1.85 (1.43 to 2.40)	
	**Ethnicity (vs. White)**				
	• Indian	1.33 (0.78 to 2.26)		1.33 (0.78 to 2.26)	
	• Unknown	0.18 (0.08 to 0.37)		0.18 (0.08 to 0.37)	
	Baseline HbA_{1c}	1.18 (1.14 to 1.21)		1.17 (1.14 to 1.21)	
**Model 4 (10-year)**	**Severity score** (C10/SW5)	1.18 (1.12 to 1.24)	0.7225	1.13 (1.10 to 1.17)	0.7233
	**Age**	1.05 (1.04 to 1.06)		1.05 (1.04 to 1.06)	
	**Gender (F)**	1.06 (0.91 to 1.24)		1.09 (0.93 to 1.27)	
	**Deprivation** (Q5 vs. Q1)	1.88 (1.45 to 2.43)		1.86 (1.44 to 2.41)	
	**Ethnicity (vs. White)**				
	• Indian	1.34 (0.78 to 2.28)		1.34 (0.78 to 2.28)	
	• Unknown	0.17 (0.08 to 0.37)		0.18 (0.08 to 0.37)	
	Baseline HbA_{1c}	1.17 (1.14 to 1.21)		1.17 (1.14 to 1.21)	
**Model 4 (5-year)**	**Severity score** (C5/SW5)	1.19 (1.12 to 1.26)	0.7209	1.15 (1.11 to 1.19)	0.7218
	**Age**	1.05 (1.04 to 1.06)		1.05 (1.04 to 1.06)	
	**Gender (F)**	1.05 (0.90 to 1.23)		1.08 (0.92 to 1.25)	
	**Deprivation** (Q5 vs. Q1)	1.88 (1.45 to 2.43)		1.87 (1.44 to 2.42)	
	**Ethnicity (vs. White)**				
	• Indian	1.36 (0.80 to 2.31)		1.36 (0.80 to 2.31)	
	• Unknown	0.17 (0.08 to 0.36)		0.18 (0.08 to 0.37)	
	Baseline HbA_{1c}	1.17 (1.14 to 1.21)		1.17 (1.14 to 1.20)	

Zghebi SS, *et al. BMJ Open Diab Res Care* 2020; 8:e000962. doi: 10.1136/bmjdrc-2019-000962

Supplementary data to "Development and validation of the DIabetes Severity SCOre (DISSCO)". Zghebi et al.

Table S23: Illustrative paper-based version of simple count DISSCO calculation

Patient number	Index date (T2DM diagnosis)	Severity domain	Domain eligible for baseline look-back window?			Baseline severity score at			Domain eligible for 3-year post-index window?			Post-index severity score at 3-year post-index +		
			5-yr window	10-yr window	Unlimited window	5-yr look-back	10-yr look-back	Unlimited window	+5-yr look-back window*	+10-yr look-back window[&]	+Unlimited look-back window[$]	5- yr look-back window	10- yr look-back window	Unlimited look-back window
01	01/03/2008	MI 03/09/1994	No	No	**Yes**				No	No	**Yes**			
		TIA 17/08/1995	No	No	**Yes**				No	No	**Yes**			
		Stroke 02/05/2002	No	**Yes**	**Yes**	1	2	4	No	**Yes**	**Yes**	2	3	5
		PCI 22/11/2006	**Yes**	**Yes**	**Yes**				**Yes**	**Yes**	**Yes**			
		CABG 26/07/2010	-	-	-				**Yes**	**Yes**	**Yes**			
02	15/07/2010	PVD 17/04/2013	-	-	-	0	0	0	**Yes**	**Yes**	**Yes**	1	1	1
03	21/10/2011	AF 31/05/2000	No	No	**Yes**	0	0	1	No	No	**Yes**			
		Stroke 06/12/2012	-	-	-				**Yes**	**Yes**	**Yes**	1	1	2

AF: Atrial fibrillation**; CABG:** coronary artery bypass graft; **MI**: myocardial infarction**; PCI:** percutaneous coronary intervention**; PVD**: peripheral vascular disease; **T2DM**: type 2 diabetes mellitus; **TIA:** transient ischemic attack.
*the presented data are for illustrative purpose only based on fictional patient IDs

Supplementary data to "Development and validation of the DIabetes Severity SCOre (DISSCO)". Zghebi et al.

Figure S1 Diagram of the look-back windows of pre-index and moving post-index time points

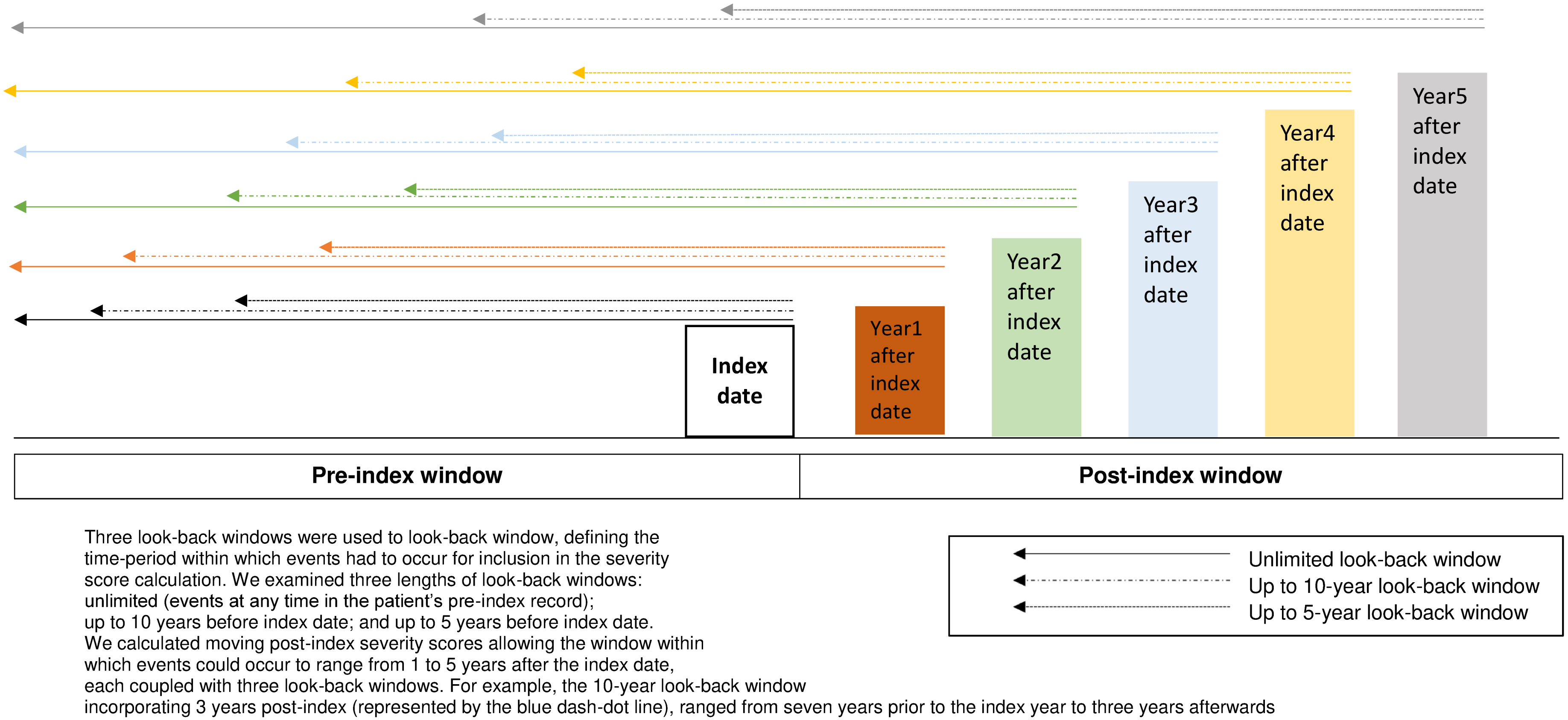

Three look-back windows were used to look-back window, defining the time-period within which events had to occur for inclusion in the severity score calculation. We examined three lengths of look-back windows: unlimited (events at any time in the patient's pre-index record); up to 10 years before index date; and up to 5 years before index date. We calculated moving post-index severity scores allowing the window within which events could occur to range from 1 to 5 years after the index date, each coupled with three look-back windows. For example, the 10-year look-back window incorporating 3 years post-index (represented by the blue dash-dot line), ranged from seven years prior to the index year to three years afterwards

Supplementary data to "Development and validation of the DIabetes Severity SCOre (DISSCO)". Zghebi et al.

Figure S2. Patients count by (A) simple count and (B) severity-weighted scores at three look-back time windows (training dataset)

(A)Simple count scores

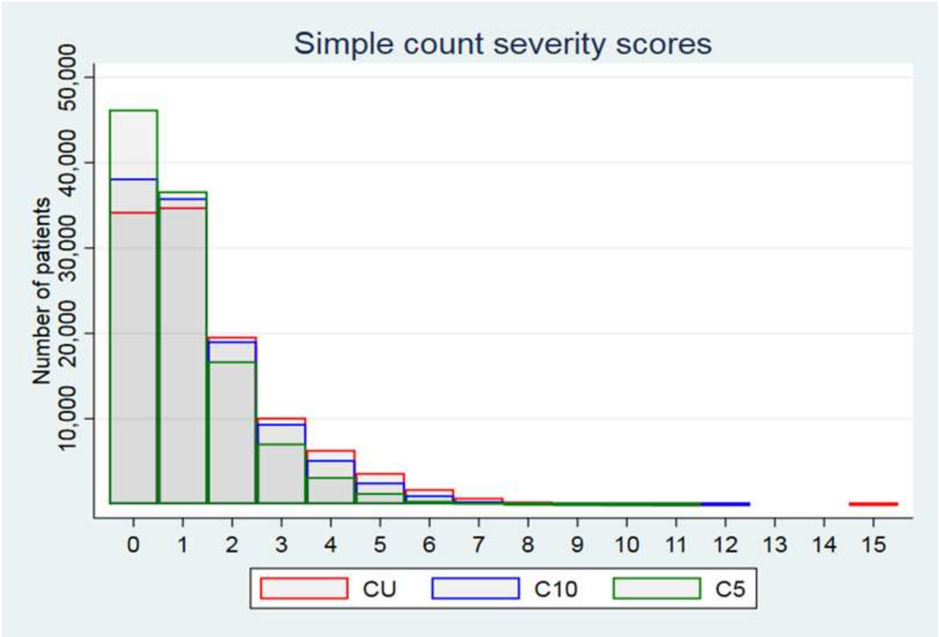

(B) Severity-weighted scores

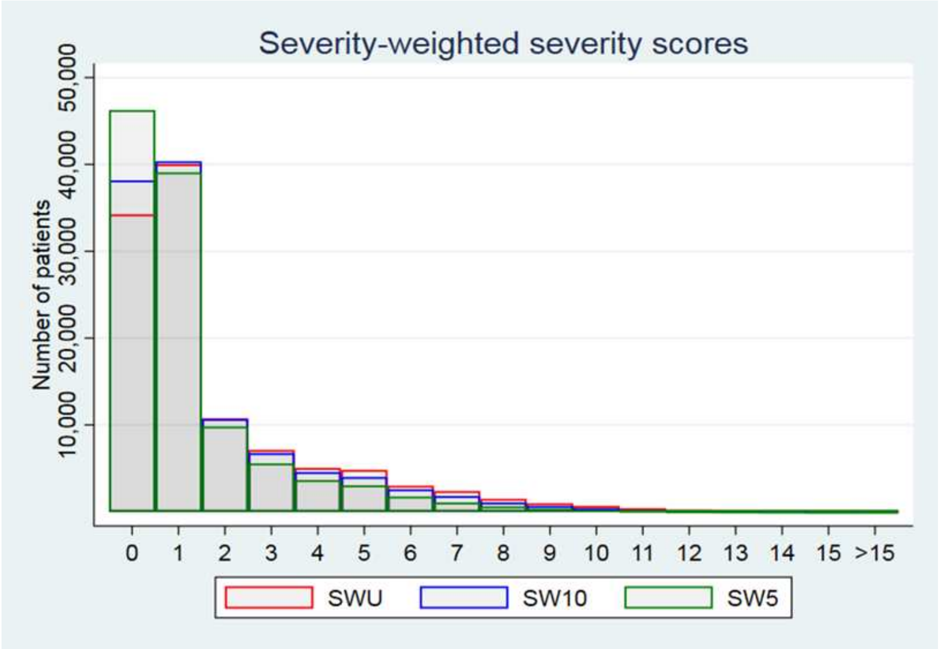

Simple count (C) or severity-weighted (SW) score measured using unlimited (CU/SWU), 10-year (C10/SW10), and 5-year (C5/SW5) look-back pre-index windows, respectively. Simple count: equals the total severity domains by assigning equal weighting (weight=1) to all domains in the overall score. Severity-weighted score: by assigning a weight to each of the severity domains according to a hierarchy of increased severity based on clinical judgement.

Supplementary data to "Development and validation of the DIabetes Severity SCOre (DISSCO)". Zghebi et al.

Figure S3 Count of patients by <u>simple count</u> severity score at moving post-index windows (training dataset)

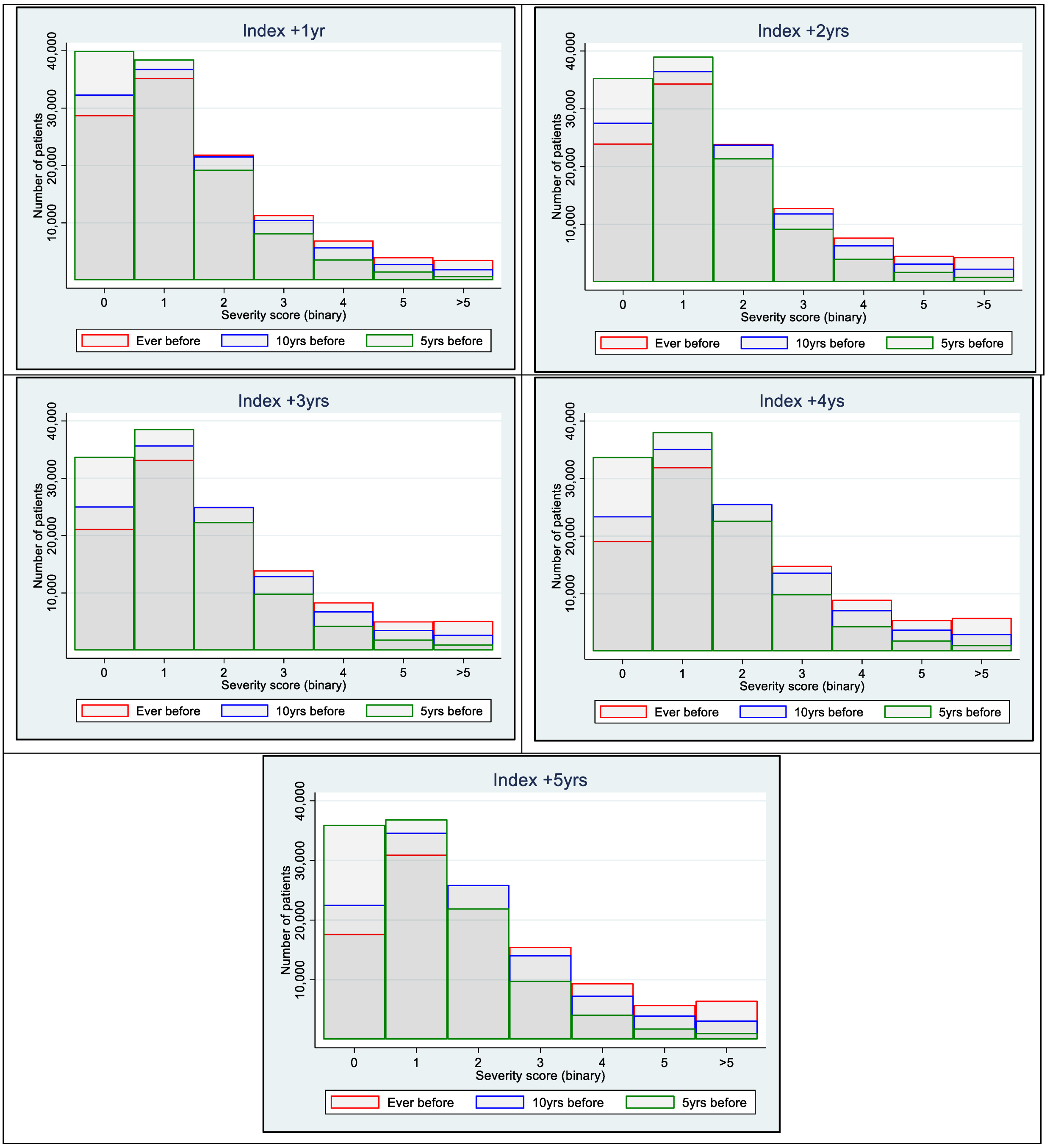

Supplementary data to "Development and validation of the DIabetes Severity SCOre (DISSCO)". Zghebi et al.

Figure S4 Count of patients by severity-weighted severity score at moving post-index windows (training dataset)

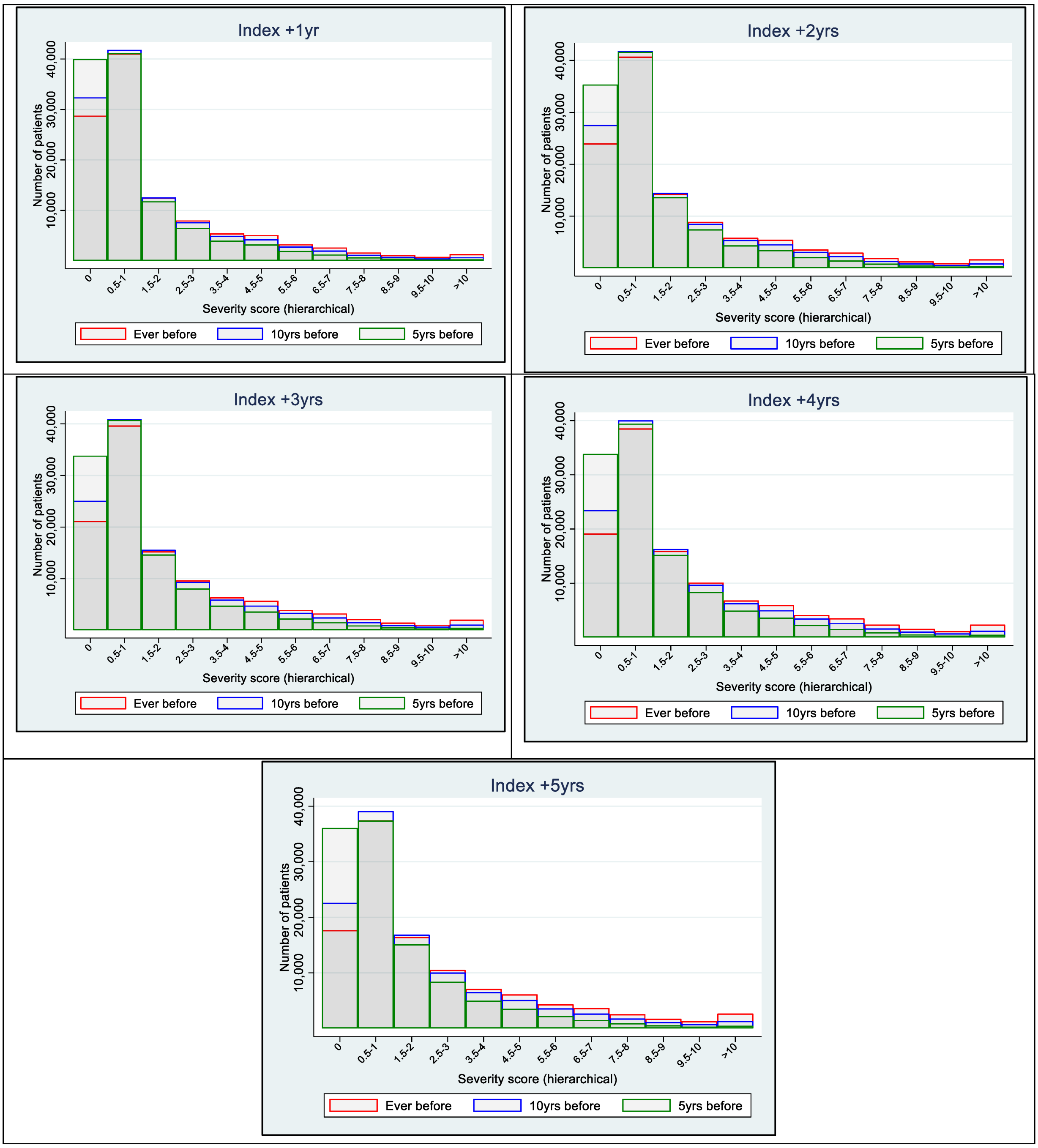

Supplementary data to "Development and validation of the DIabetes Severity SCOre (DISSCO)". Zghebi et al.

Figure S5 Schoenfeld residuals for testing proportional hazards of fitted survival models using C10 severity score (by gender) – training dataset

Panel A) All-cause mortality

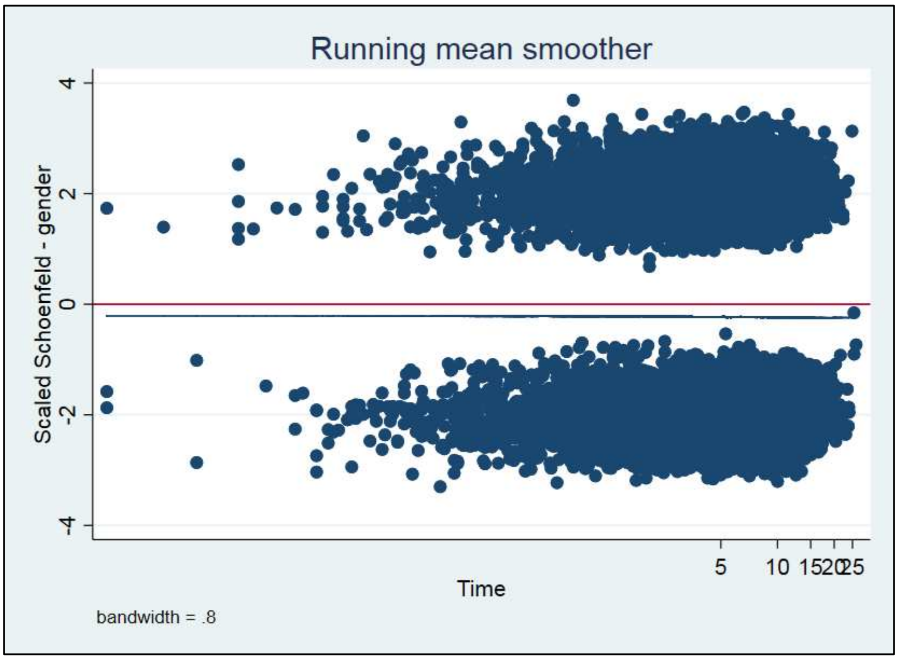

Panel B) All-cause hospitalisation

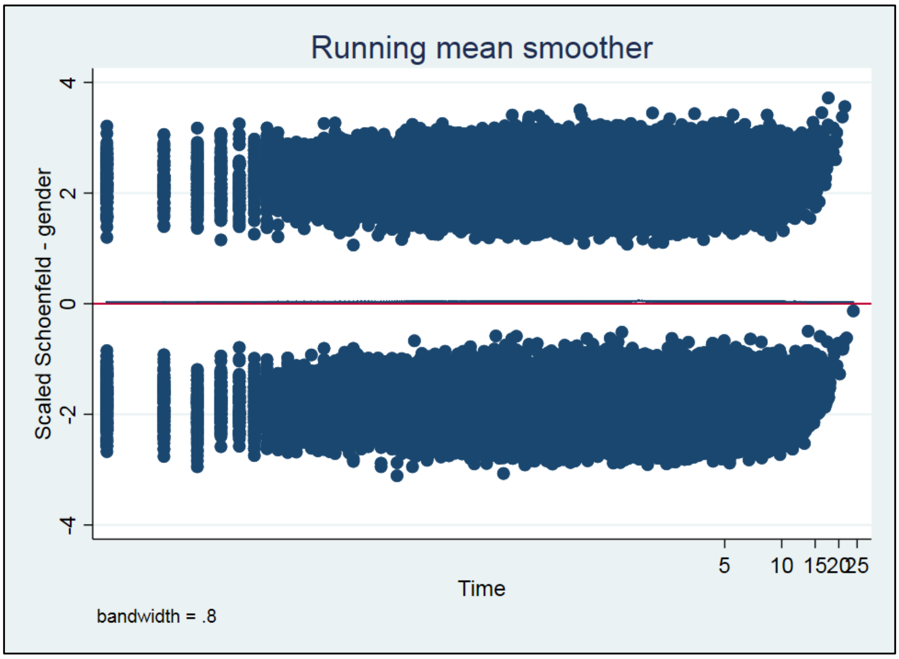

Supplementary data to "Development and validation of the DIabetes Severity SCOre (DISSCO)". Zghebi et al.

Panel C) CV hospitalisation

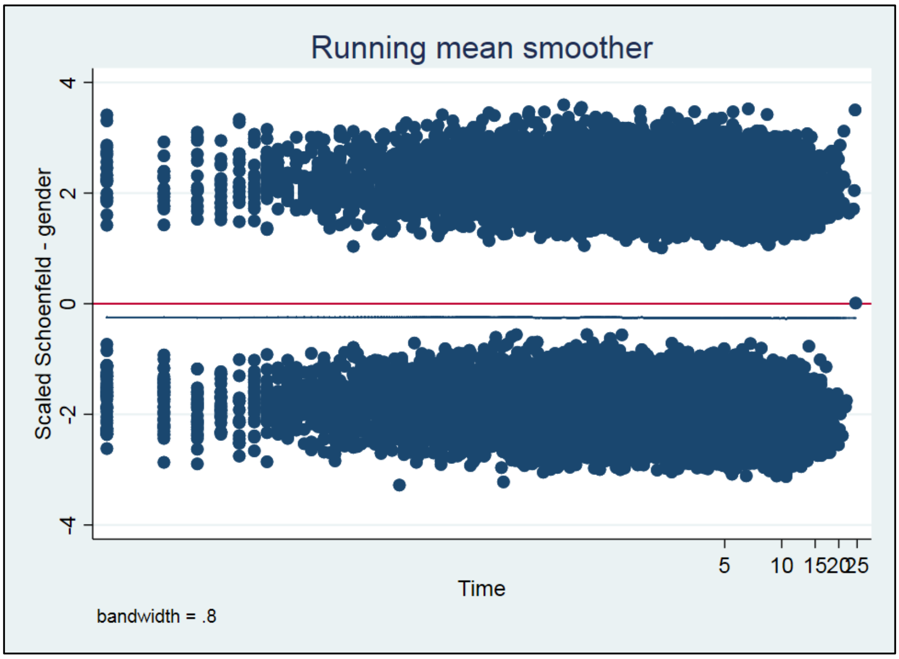

Panel D) Diabetes-related hospitalisation

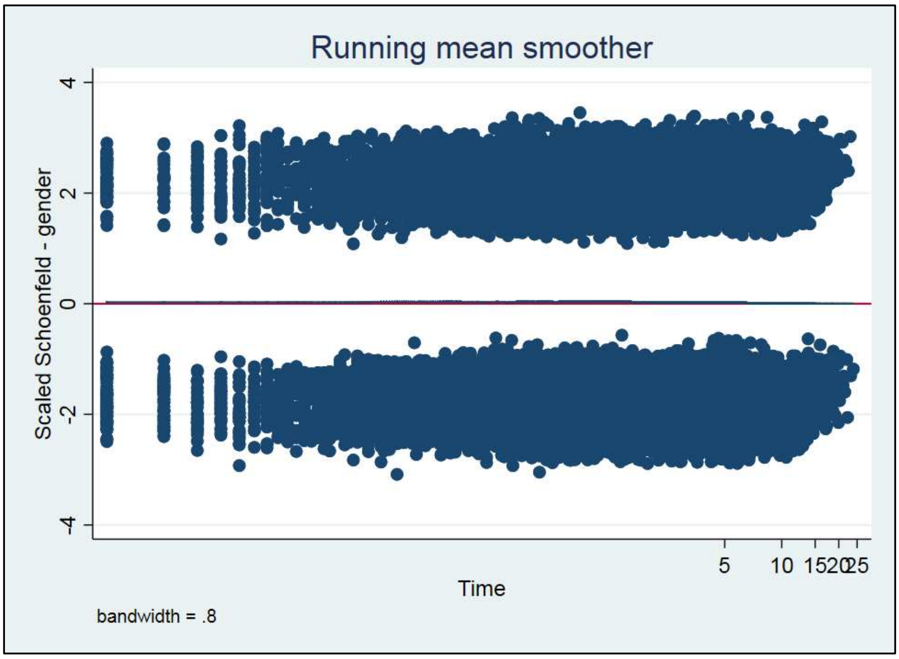

Zghebi SS, *et al. BMJ Open Diab Res Care* 2020; 8:e000962. doi: 10.1136/bmjdrc-2019-000962

Supplementary data to "Development and validation of the DIabetes Severity SCOre (DISSCO)". Zghebi et al.

Panel E) Hypoglycemia hospitalisation

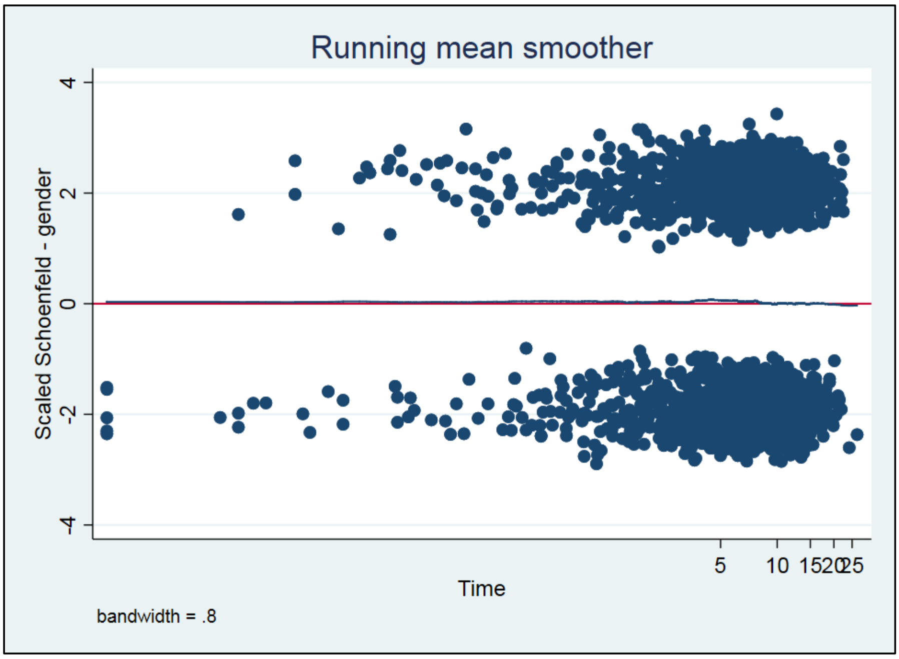

Panel F) Clustered CV/diabetes hospitalisation

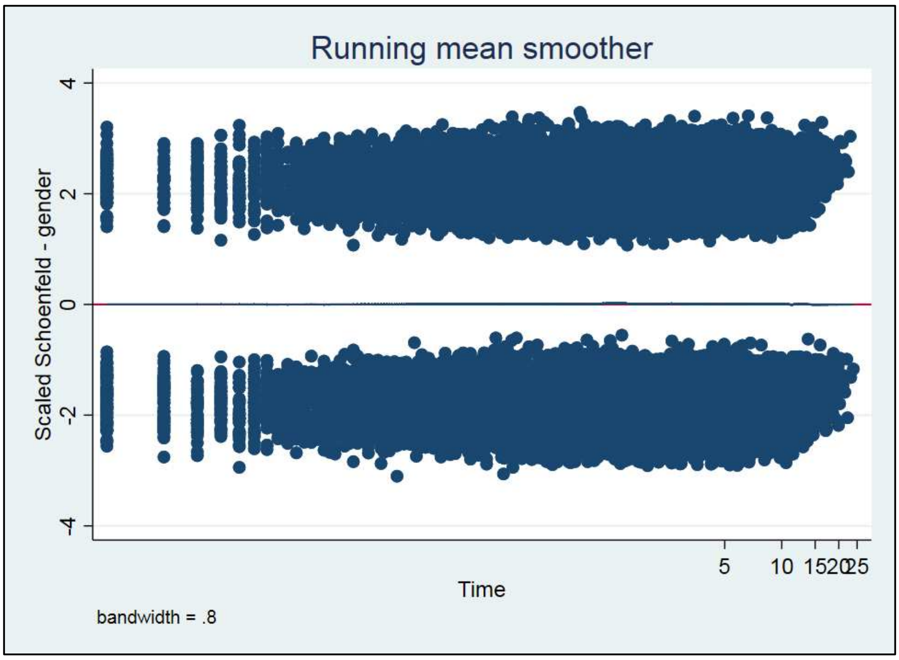

Supplementary data to "Development and validation of the DIabetes Severity SCOre (DISSCO)". Zghebi et al.

Panel G) CV procedures

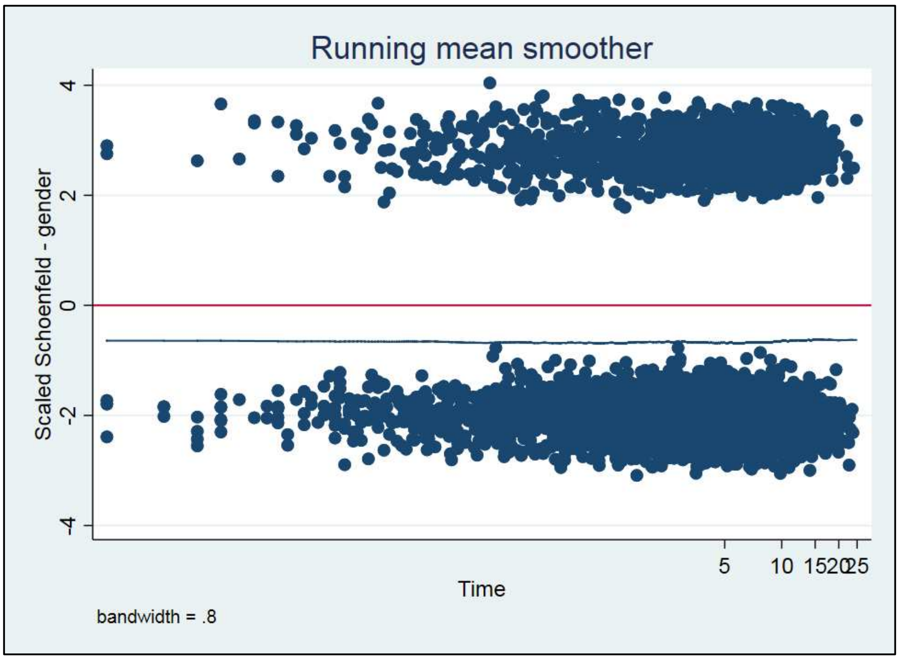

Zghebi SS, *et al. BMJ Open Diab Res Care* 2020; 8:e000962. doi: 10.1136/bmjdrc-2019-000962

Supplementary data to "Development and validation of the DIabetes Severity SCOre (DISSCO)". Zghebi et al.

Figure S6 Schoenfeld residuals for testing proportional hazards of fitted survival models using C10 severity score (by gender) – validation dataset

Panel A) All-cause mortality

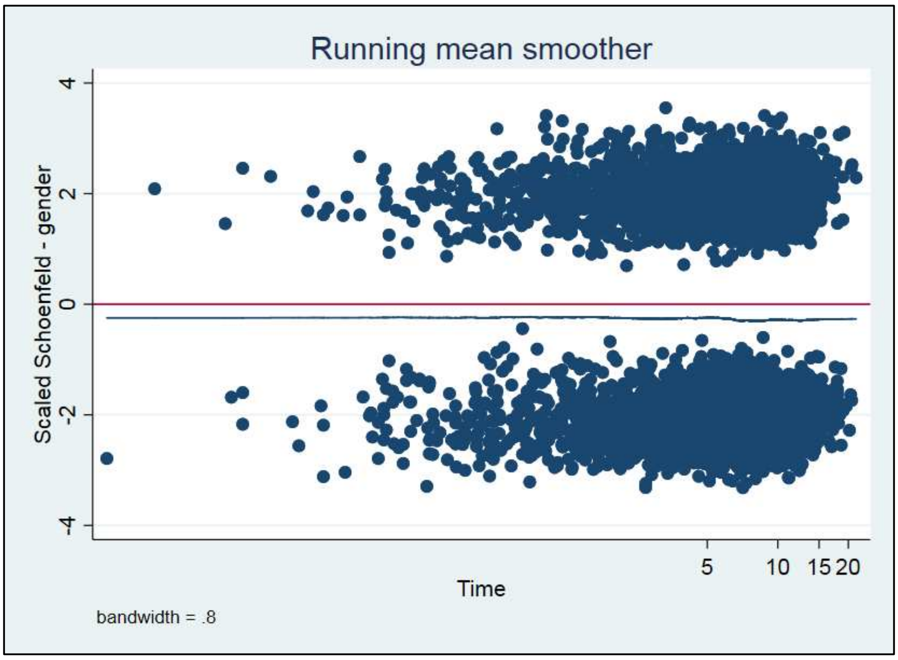

Panel B) All-cause hospitalisation

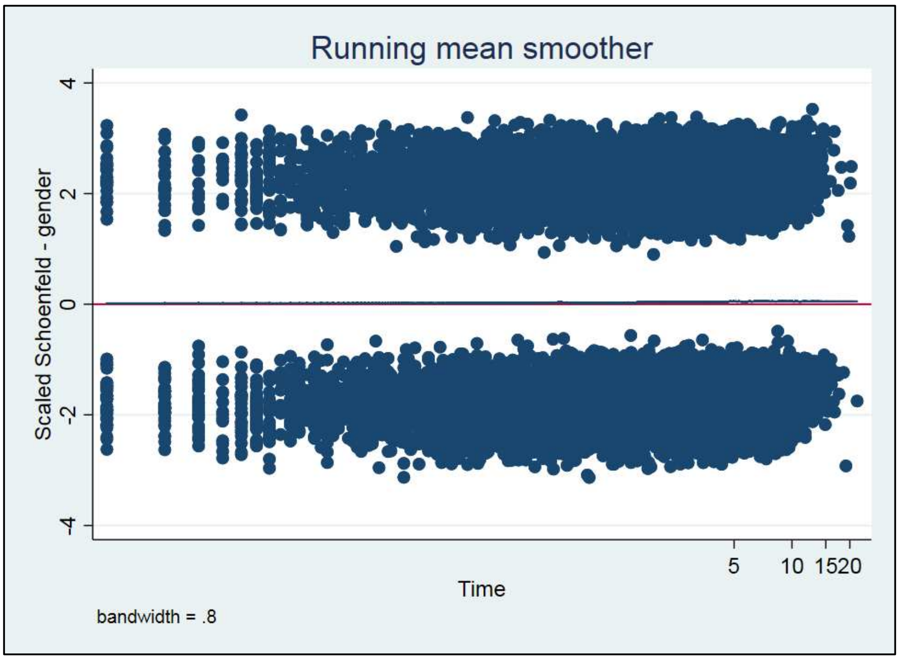

Zghebi SS, *et al. BMJ Open Diab Res Care* 2020; 8:e000962. doi: 10.1136/bmjdrc-2019-000962

Supplementary data to "Development and validation of the DIabetes Severity SCOre (DISSCO)". Zghebi et al.

Panel C) CV hospitalisation

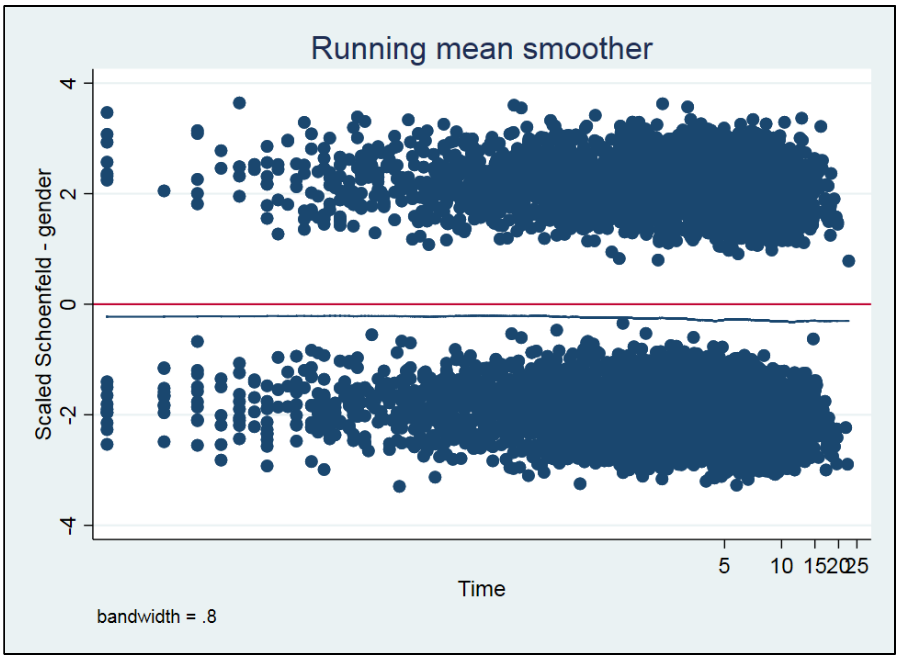

Panel D) Diabetes-related hospitalisation

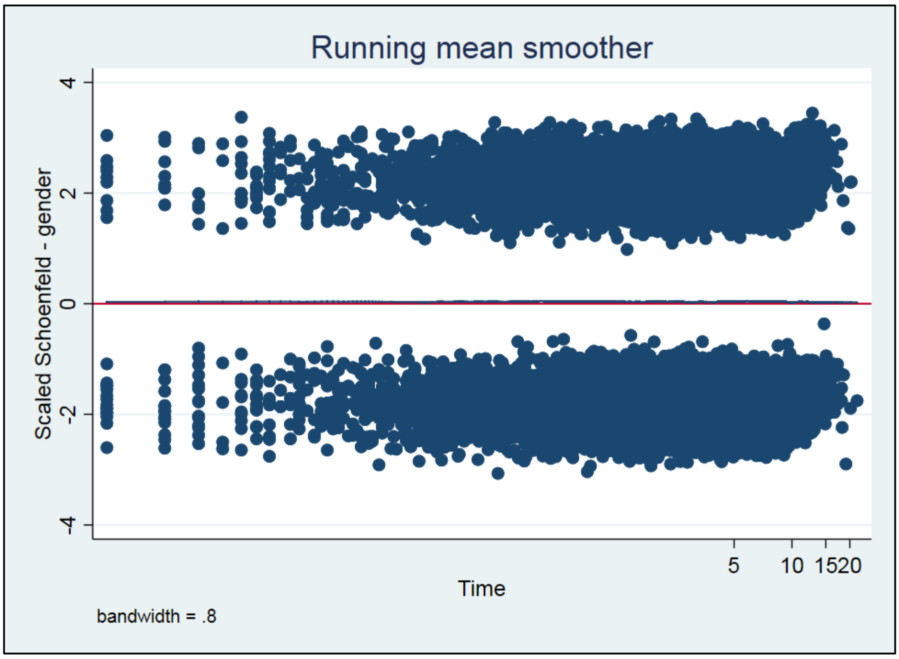

Zghebi SS, *et al. BMJ Open Diab Res Care* 2020; 8:e000962. doi: 10.1136/bmjdrc-2019-000962

Supplementary data to "Development and validation of the DIabetes Severity SCOre (DISSCO)". Zghebi et al.

Panel E) Hypoglycemia hospitalisation

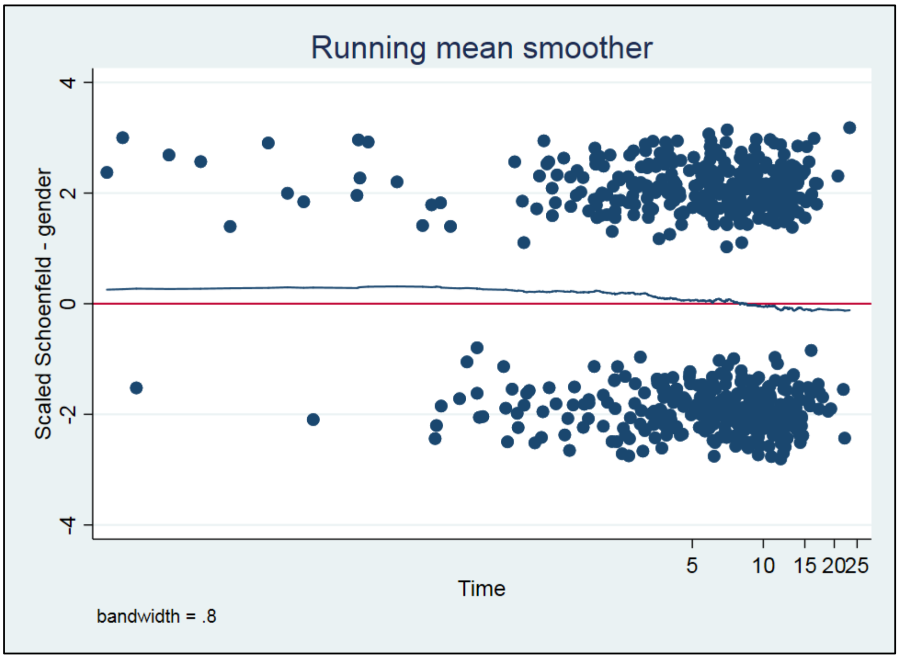

Panel F) Clustered CV/diabetes hospitalisation

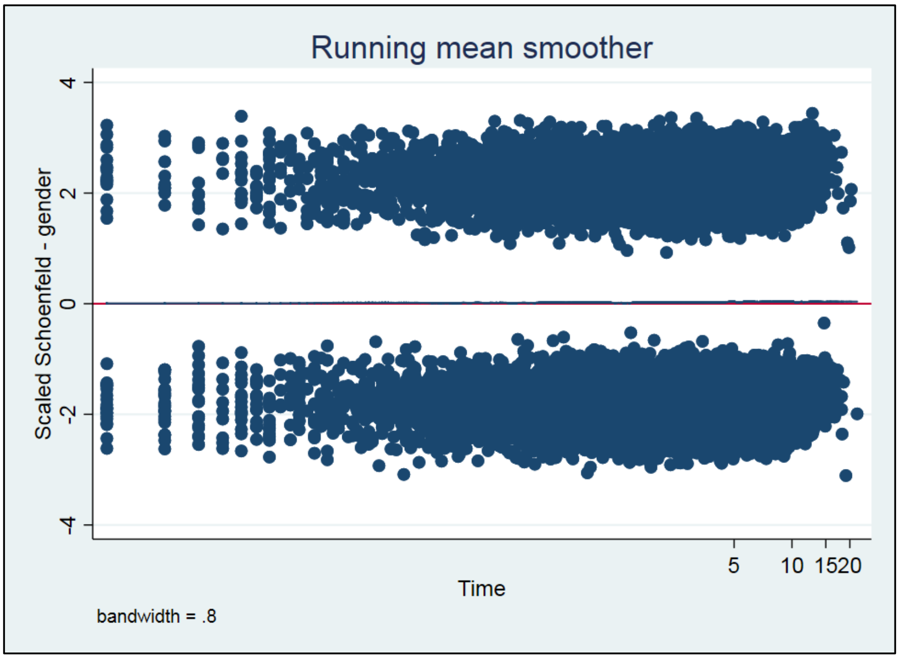

Supplementary data to "Development and validation of the DIabetes Severity SCOre (DISSCO)". Zghebi et al.

Figure S7 Bar charts of estimated AUROC for survival models including HbA_{1c} and sociodemographics variables with and without DISSCO

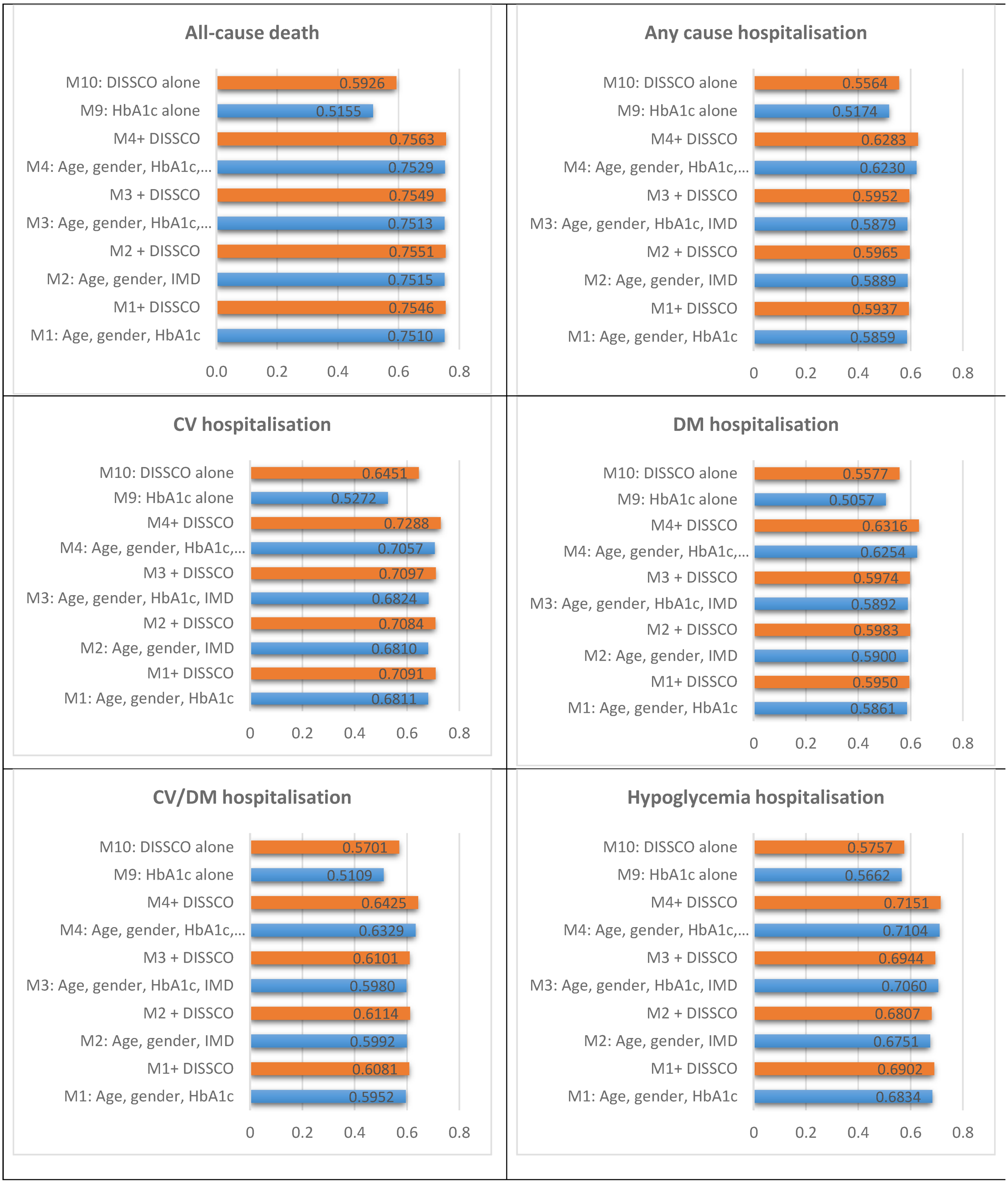

Zghebi SS, *et al*. *BMJ Open Diab Res Care* 2020; 8:e000962. doi: 10.1136/bmjdrc-2019-000962

Supplementary data to "Development and validation of the DIabetes Severity SCOre (DISSCO)". Zghebi et al.

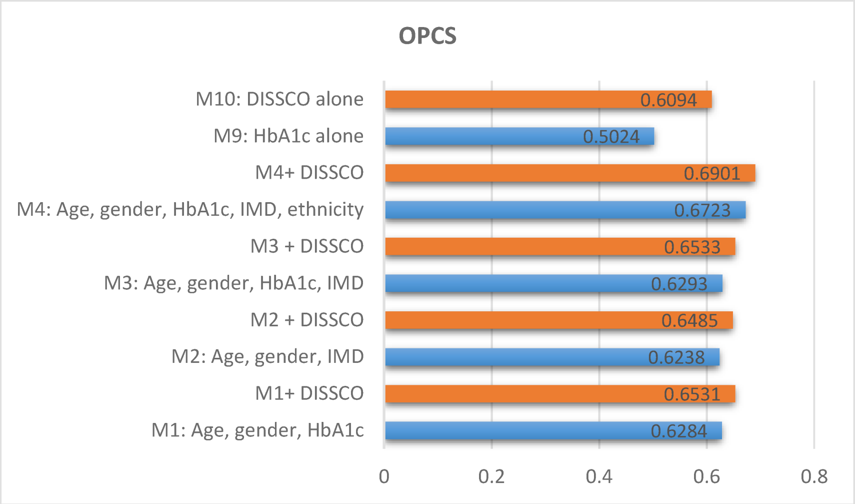

Zghebi SS, *et al. BMJ Open Diab Res Care* 2020; 8:e000962. doi: 10.1136/bmjdrc-2019-000962

Supplementary data to "Development and validation of the DIabetes Severity SCOre (DISSCO)". Zghebi et al.

Figure S8 100% Stacked bar charts of estimated AUROC for survival models including HbA$_{1c}$ and sociodemographics variables with and without DISSCO

Panel A) Stacked bar charts comparing AUROC for univariable survival models of HbA$_{1c}$ and DISCCO for adverse outcomes

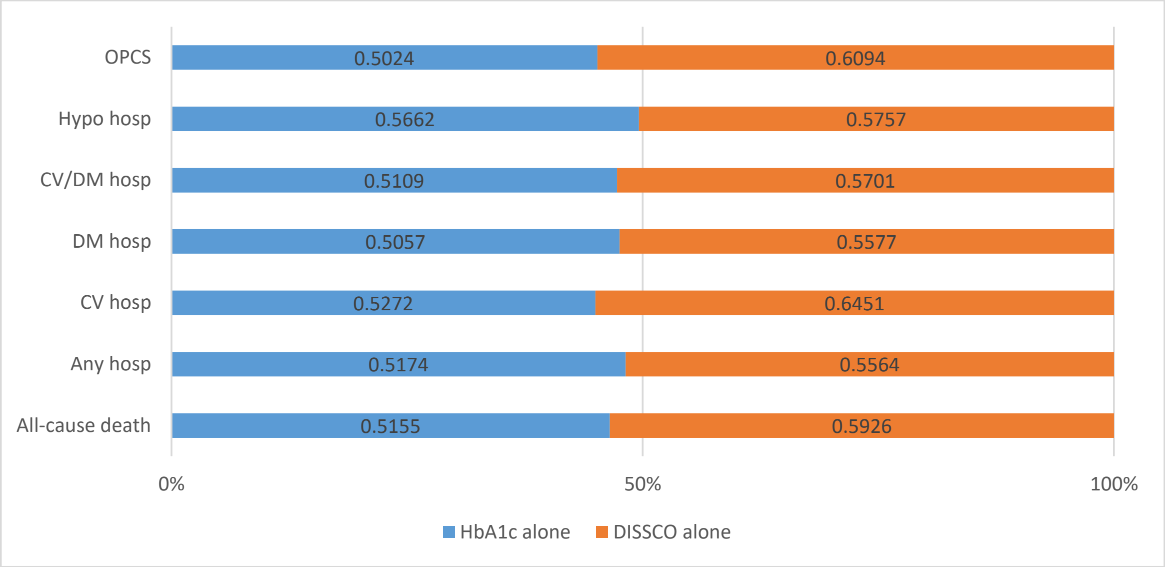

Panel B) Stacked bar charts comparing AUROC for survival models of sociodemographics variables and HbA$_{1c}$ with and without DISSCO for adverse outcomes

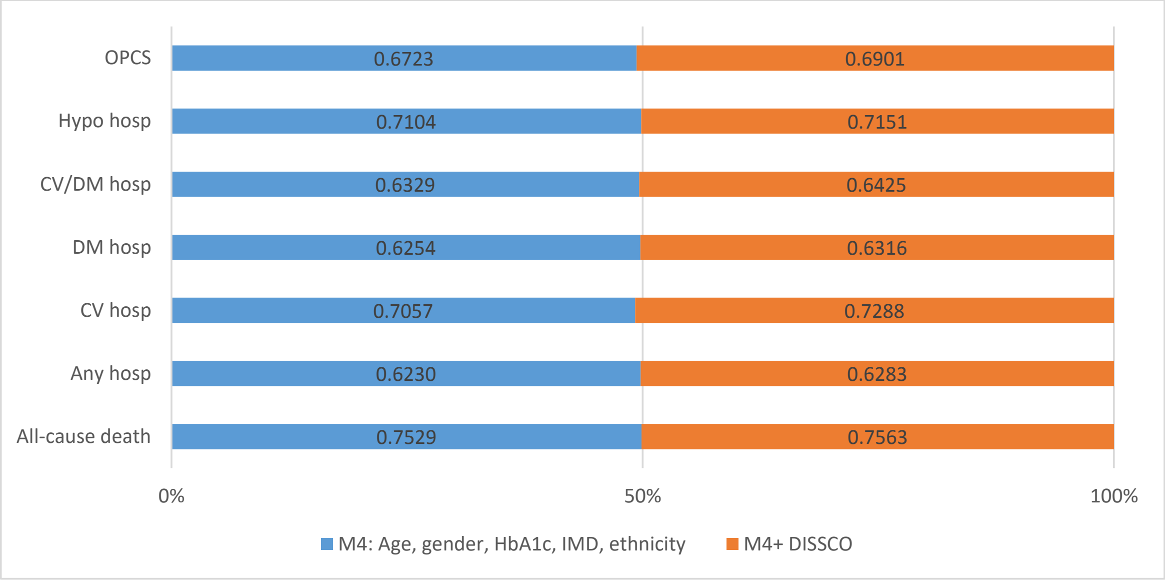

Supplementary data to "Development and validation of the DIabetes Severity SCOre (DISSCO)". Zghebi et al.

Figure S9 Kaplan-Meier plots for risk of adverse outcomes associated with 10-year (C10) severity score categories - Validation dataset

(A) All-cause mortality

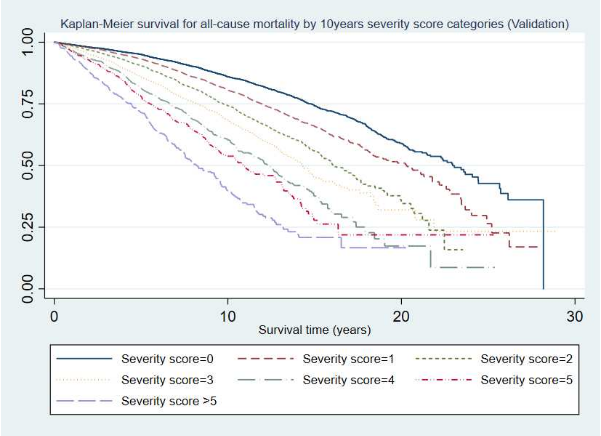

(B) Any cause hospitalisation

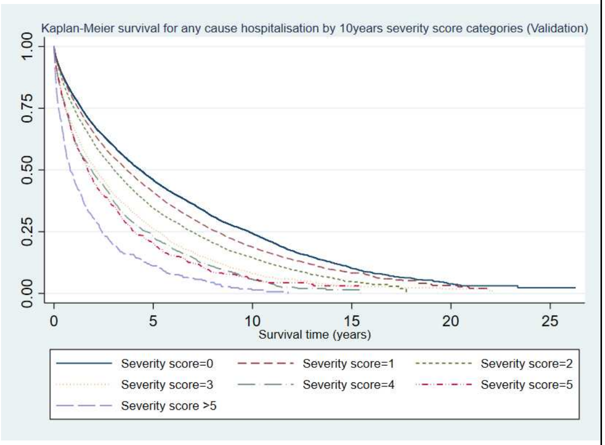

(C) CV-related hospitalisation

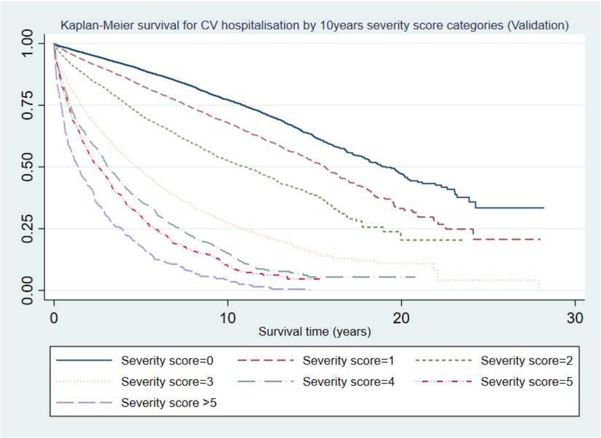

(D) Diabetes related hospitalisation

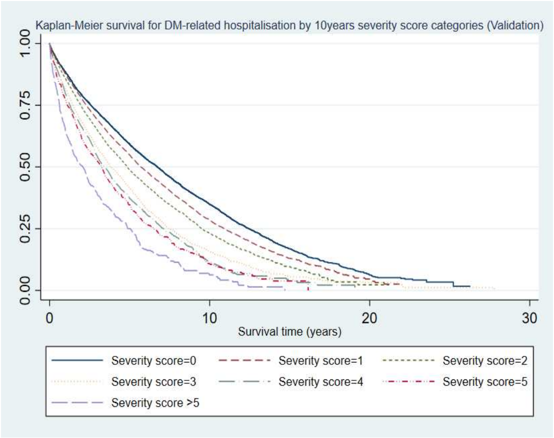

(E) Hypoglycaemia-related hospitalisation

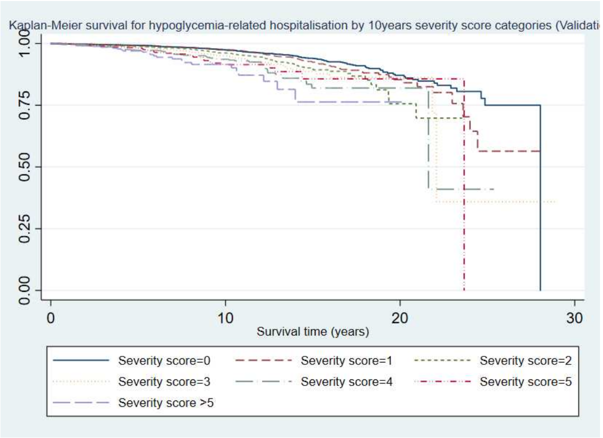

(F) Clustered CV-diabetes related hospitalisation

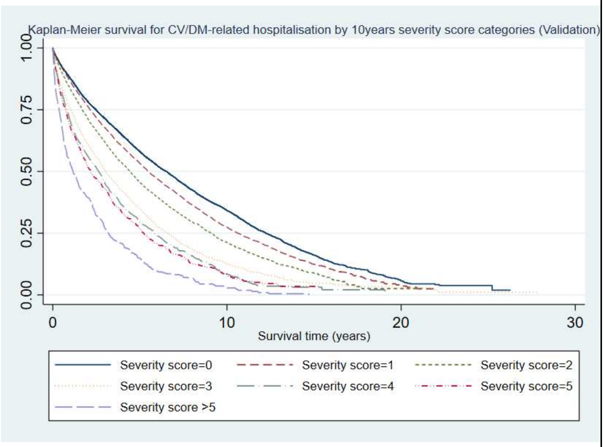

Supplementary data to "Development and validation of the DIabetes Severity SCOre (DISSCO)". Zghebi et al.

Figure S10 Calibration test by prediction of population-averaged survival probabilities for simple count scores for all-cause mortality

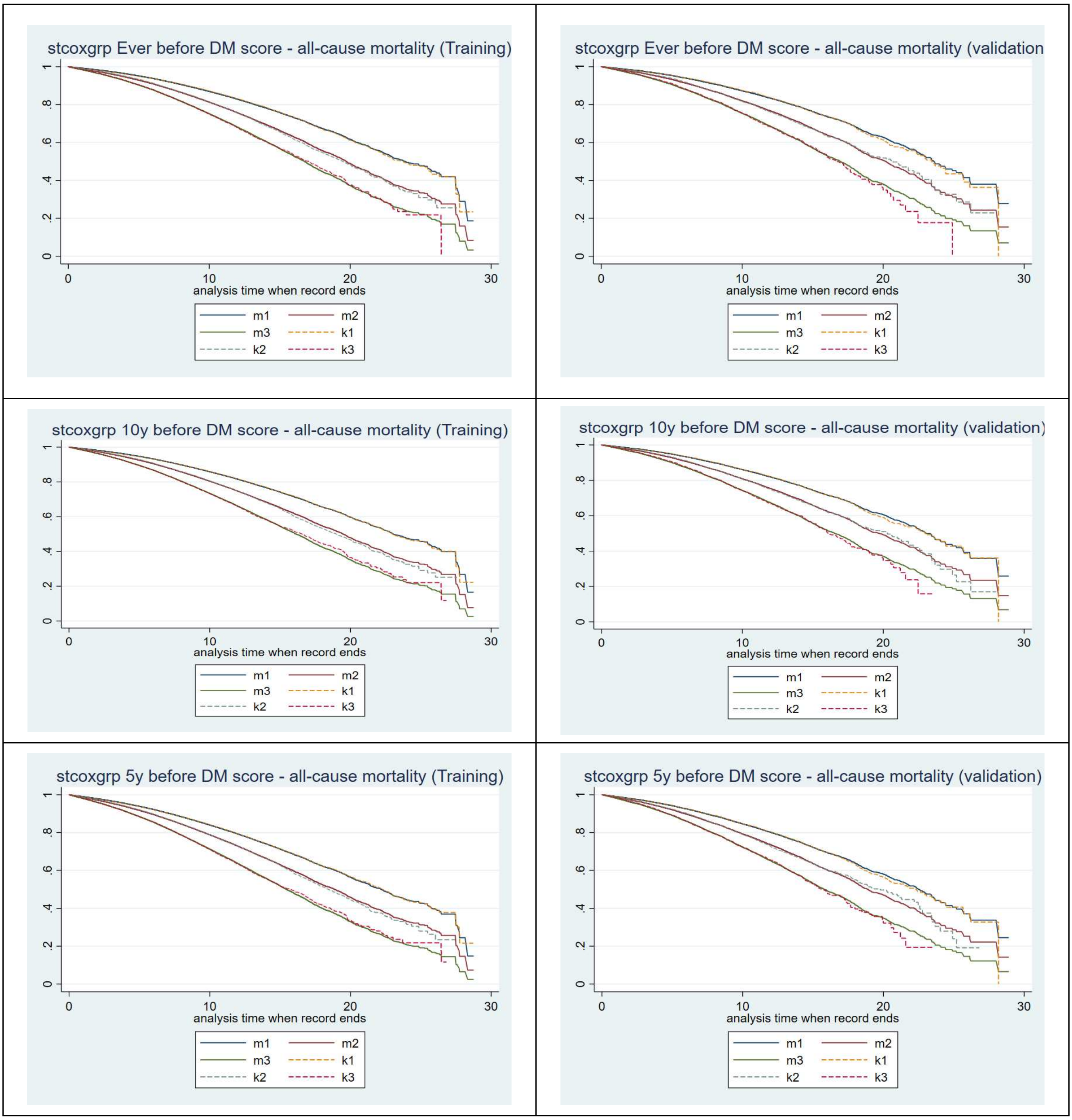

Supplement: Supplementary data [file bmjdrc-2019-000962supp001.pdf]
